# A global analysis of the rise, reign, and retreat of topics in research toward sustainable platform chemicals

**DOI:** 10.1039/d5gc02863a

**Published:** 2025-10-02

**Authors:** Paul Tautorat, Benedetta Tremolada, Antonio J. Martín, Lucas F. Santos, Gonzalo Guillén-Gosálbez, Javier Pérez-Ramírez, Bjarne Steffen

**Affiliations:** a Climate Finance and Policy Group, Department of Humanities, Social and Political Sciences, ETH Zürich Clausiusstrasse 37 8092 Zurich Switzerland bjarne.steffen@gess.ethz.ch; b Energy and Technology Policy Group, Department of Humanities, Social and Political Sciences, ETH Zürich Clausiusstrasse 37 8092 Zurich Switzerland; c Institute for Chemical and Bioengineering, Department of Chemistry and Applied Biosciences, ETH Zürich Vladimir Prelog Weg 1 Zürich 8093 Switzerland; d NCCR Catalysis Switzerland

## Abstract

Platform chemicals—such as olefins, ammonia, aromatics, and methanol—serve as fundamental building blocks for the chemical industry. At the same time, they account for 4% of global CO_2_ emissions, highlighting the need for renewable feedstocks, renewable energy, or other alternative approaches to develop more sustainable production routes toward carbon neutrality. Despite substantial research output, we lack a holistic understanding of where innovation is heading. This gap leaves research planning and policy decisions without a quantitative basis for understanding the directions of innovation. A better understanding of how they differ between platform chemicals is critical for delineating policies and strategic plans toward a net-zero future. Our study addresses this gap by providing unprecedented clarity on global research trends in platform chemicals spanning the last three decades since the establishment of the Green Chemistry principles and other subsequent sustainability approaches for chemical systems, showing the rise, reign, and retreat of topics in this area. For this purpose, we develop a novel approach by integrating topic modelling, generative AI, and expert judgment to analyse >90 000 research articles from Scopus, identifying 62 distinct research topics and tracking their temporal and geographical trends. Our results reveal different innovation patterns across the four platform chemicals. Driven by the concepts of an ammonia or methanol economy, research output has increased for these platform chemicals by a factor of 17 and 6 between 2000 and 2024, respectively. This growth has been led by new strategies like photo- and electrochemical routes, which now account for approximately 65% of ammonia-related research. For olefins and aromatics, innovation patterns show less momentum as research has rather focused on optimising available technologies. Reliance on existing alternative routes (based on renewable methanol) and olefins and aromatics’ molecular complexity could explain this lower momentum. Our quantitative findings can help define research priorities for green chemistry and derive the implications of emerging technological trends on industrial systems regarding future electricity, biomass, and feedstock demand.

## Abstract 2

We analyse global research trends in the sustainable production of ammonia, methanol, olefins, and aromatics by integrating topic modelling, generative AI, and expert judgment to analyse over 90 000 research articles spanning three decades.

## Graphical abstract

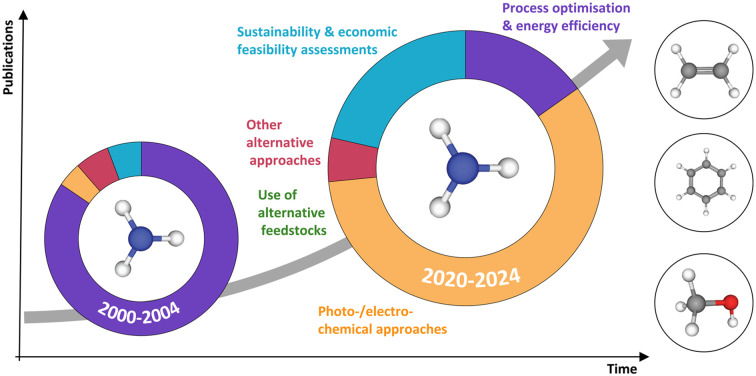

**Green foundation**

1. This work presents the first quantitative analysis of global research trends in the sustainable production of platform chemicals—olefins, ammonia, aromatics, and methanol—by integrating topic modelling, generative AI, and expert judgment to analyse over 90 000 research articles spanning three decades since the emergence of Green Chemistry principles.

2. The study reveals divergent innovation patterns since 2000: ammonia and methanol research output has surged by factors of 17 and 6, respectively, mainly driven by emerging photo- and electrochemical routes. In contrast, olefins and aromatics show less momentum and more incremental innovation focused on optimizing existing technologies, constrained by molecular complexity and methanol-based routes.

3. Future work should assess emerging sustainable technologies using quantitative metrics from early stages to establish hierarchies, explore system-level impacts on demands of renewable energy and feedstocks, and guide research and policy priorities to accelerate the transition toward carbon-neutral platform chemical production.

## Introduction

The chemical industry is a success story and a pillar of modern society.^1–3^ However, its fossil dependence is also partly responsible for the current climate crisis,^4,5^ making it necessary to transform the chemical industry towards sustainability, allowing us to limit and hopefully reverse the negative consequences.^3,6,7^ Prioritisation of efforts based on sustainability metrics will pave the way towards this objective.^8^ Most of the environmental footprint of the chemical sector can be traced back to a set of *platform chemicals*, namely olefins, ammonia, aromatics, and methanol, which are indispensable for manufacturing a wide range of products used in daily life, from fertilisers and fuels to plastics and paints.^9,10^ These chemicals are produced at large scales—290, 185, 116, and 102 Mt per year, respectively—with production expected to increase even further.^5,9^ Given their large production volumes, they are responsible for 2.3 (cradle-to-gate) of the global 59 Gt CO_2_-eq emissions emitted annually, making their sustainable production essential to achieving a carbon-neutral chemical industry.^4,11^ The importance of a more sustainable platform chemical production expands beyond the chemical industry, as these products could also support the decarbonisation of other sectors. Ammonia could serve as a carrier to transport and store hydrogen.^12–17^ Methanol-based fuels could help to defossilise shipping or aviation.^15,18–20^ Olefins, if produced from non-fossil carbon,^21–27^ could reduce the full lifecycle carbon footprint of incinerated plastics.^24,28–30^

The incumbent production routes for these platform chemicals, like the Haber–Bosch process for ammonia, have been researched and refined over decades and are deeply embedded in industrial systems.^9,31^ The increasing awareness of environmental sustainability crystallised *ca.* three decades ago in the Green Chemistry principles,^32^ leading shortly afterwards to the establishment of journals like Green Chemistry to foster a rising research community. This conceptual framework was complemented with other approaches aiming to improve the sustainability level of industrial systems, including life cycle assessment.^33^ Alternative routes discussed to improve sustainability expanded beyond increasing efficiency into other areas such as switching to renewable feedstocks or fuels^34^ (incl. low carbon electricity), and applying carbon capture, and recycling.^4,35,36^ However, the technological maturity of these alternative routes is sometimes low and feedstock prices are not competitive with fossil alternatives, making that in the absence of incentive schemes, their economics are seldom competitive. Current abatement costs using these routes are often high, up to several hundred Euro per ton of CO_2_ abated.^37^ However, hopes are high that abatement costs can be reduced through technological advancements and policymaking, including through academic and industrial research (dubbed “learning-by-searching” in innovation theory). Policymakers aim to support such efforts through R&D funding, though selecting the right research topics is often not straightforward.

While there is a wealth of research on producing platform chemicals, it is challenging for researchers in academia and industry, managers, and policymakers to stay updated on recent trends across all chemicals, routes, and approaches, likely resulting in a fragmented understanding of their potential. However, a holistic understanding is important as fuels, feedstocks, and infrastructure needed for the competing routes differ fundamentally—from low-carbon electricity and renewable heating to defossilised feedstocks and chemical recycling.^38^ Accordingly, there is a gap in the literature for a quantitative basis for objectively capturing research on producing platform chemicals. Quantifying relevant literature can enable further analysis of the different routes in the context of technological change and innovation.^39–44^ When combined with quantitative life cycle assessment^5,45–47^ of nascent production routes and an understanding of how technologies learn and scale,^48–50^ such an analysis is valuable for helping grant offices in identifying gaps, informing demand policies like EU's sustainable aviation fuel mandate,^51,52^ improving the representation of nascent technologies in energy system models,^53,54^ and guiding researchers working on the next generation of sustainable chemical processes.

In this work, we present the first quantitative review of the directions toward sustainability of olefins, ammonia, aromatics, and methanol production. Furthermore, we offer an interpretation of which topics seem to be on the rise, which seem to have the potential for long-term reign in industry, and which—despite showing potential initially or in the past—seem to be on the retreat, offering the base to establish a technological roadmap. For this purpose, we develop a novel approach by integrating topic modelling, generative AI, and expert judgment. Our results reveal different innovation patterns across the four platform chemicals. Driven by the concepts of an ammonia or methanol economy, research output has surged for these chemicals, led by new strategies like shifting from thermochemical to photo- and electrochemical routes, promising great sustainability gains. For olefins and aromatics, innovation patterns show less momentum as research has rather focused on optimising available technologies, promising only incremental sustainability gains. Reliance on existing alternative routes (based on renewable methanol) and olefins and aromatics’ molecular complexity could explain this lower momentum. For ammonia, methanol, and olefins, we also see a trend toward research assessing different approaches through metrics-based quantification. Overall, this article should serve as a basis for further discussions around trajectories for more sustainable platform chemical production.

## Methods

**Fig. 1:**
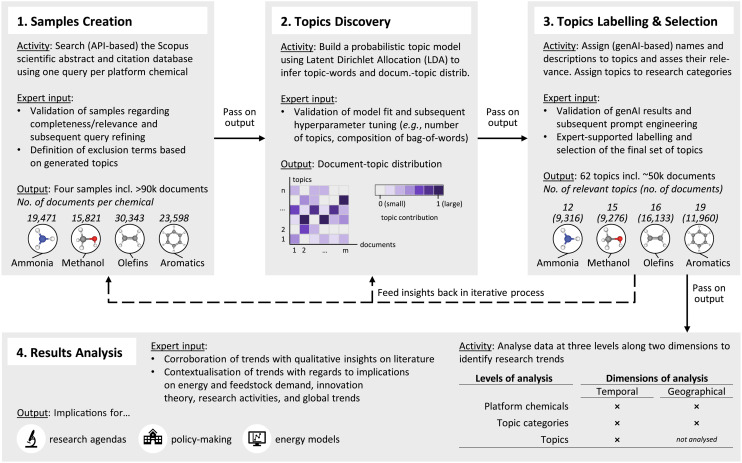
Framework of the iterative process of this study: 1. samples creation, 2. topics discovery, 3. topics labelling & selection, and 4. results analysis.

This study presents an approach that allows for the identification and quantitative analysis of global academic research related to the four groups of platform chemicals. To this end, the study uses the number of publications per year and topic, the term used in natural language processing, as a metric to quantify trends, aligning with the research community's larger push towards metrics-based orientation of research efforts.^8^ Confronted with more than 90 000 relevant publications, we implemented a highly automatic approach that uniquely integrates probabilistic topic modelling,^35,55–59^ generative AI (genAI), and expert knowledge to extract meaningful patterns and directions of innovation. Compared to traditional narrative and semi-structured reviews, our quantitative approach allows for analysing a much larger volume of publications than would be possible in a manual way, in a more reliable and replicable way.^60,61^ At the same time, relying on algorithmic analyses only would ignore highly valuable contextual expert knowledge, thereby limiting the relevance of purely quantitative results. Hence, we include human expertise from the author team at several stages in the research process. Our iterative process, as presented in Fig. 1, allows the enhancement of topic quality by refining model inputs at different stages of the process based on expert input. Please refer to the SI for more details on the four steps.

### Step 1: Samples creation with search queries

We begin by constructing tailored literature samples for each group of platform chemicals based on the academic literature database Scopus, utilising four separate search queries that we develop, iteratively tune, and test (in sections 3.2, 3.3, and 3.4 of the SI we present the queries and the test results, respectively). Our samples encompass 19 930 articles for ammonia, 16 156 for methanol, 31 582 for light olefins (in the following only referred to as *olefins*), and 24 352 for aromatics. We iteratively refine the search queries based on expert input to improve completeness (*i.e.*, maximise the coverage of our samples of the total literature) while maintaining high relevance (*i.e.*, minimise the number of falsely included articles).

### Step 2: Topics discovery with probabilistic topic modelling

We tune and apply a Latent Dirichlet Allocation (LDA) model to the samples to infer topic-word and article-topic distributions, which enables us to group articles.^35,55–59^ We assign all articles in the samples to the discovered topics, providing the starting point for our further analysis (*cf.* section 4.2 of the SI for the assignment). We iteratively refine the model's hyperparameters based on its model performance (*cf.* section 4.3 of the SI for the details on the tuning process and the model's final features).

### Step 3: Topics labelling and selection with generative AI

We use genAI to automatically label topics and assess their relevance (*cf.* section 5.1 of the SI for details on the prompting process). This accelerates the iterative process, yielding an improved set of topics after every query or model tuning. Iteration by iteration, the use of genAI reduces and the expert involvement increases. Using this methodology, we finally arrive at 62 topics and 46 685 articles, which we then analyse to reveal innovation patterns (12 topics (9316 articles) for ammonia, 15 topics (9276 articles) for methanol, 16 topics (16 133 articles) for olefins, and 19 topics (11 960 articles) for aromatics). Then we assign the 62 topics to five research categories representing different approaches towards sustainability (*cf.* section 2.3 of the SI for details on the categories).

### Step 4: Results analysis

We analyse the resulting dataset on three levels. First, we contrast high-level trends for the four platform chemicals. Second, we analyse trends for five research categories per group of platform chemicals. Third, we scrutinise trends for the 62 discovered topics. In addition to the temporal dimension, we examine the data along the geographical dimension at the chemical and category levels, allowing us to gain a comprehensive and nuanced understanding of the evolving trends over time and across different world regions. Combining these factors enables us to derive insights for research agendas, energy models, and policymaking.

### Expert input

Throughout this process, keeping the “human in the loop” *via* expert input is critical in ensuring high-quality results (*cf.* sections 3.4, 5.2, and 5.3 of the SI). Subject matter experts from the author team refine search queries to optimise sample quality, tune the probabilistic model's hyperparameters for optimal performance, and improve the generative AI's topic labelling. Expert involvement also extends to the manual refinement of topic names, assigning topics to categories, and the corroboration of quantitative trends with qualitative insights from the domain. In the discussion of results, we contextualise the identified trends with respect to energy and feedstock demand, innovation theory, research activities, and broader global trends. Finally, we complement our quantitative analysis with an expert discussion of limitations identified in recent (narrative and semi-structured) review articles, to incorporate a broader, multidimensional perspective.

## Results and discussion

### Research on platform chemicals outpaces average scientific production growth

**Fig. 2:**
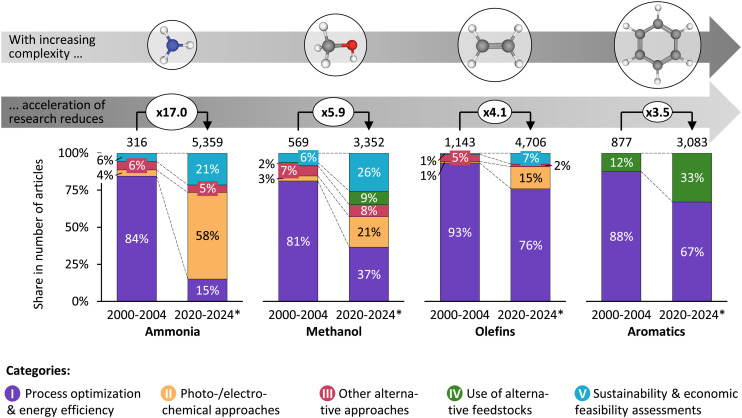
Shift in research focus per group of platform chemicals measured in the number of articles. *For 2024, articles are only considered until a mid-year cut-off date (*cf.* Table S4 of the SI). The percentage values indicate the share per category and five-year period. The values on the arrows indicate the factor increase between the two five-year periods. The chemical structures of ethylene and benzene are shown as examples for olefins and aromatics, respectively.

Over the last 25 years, the research output, here defined as the number of articles per year, on the production of platform chemicals has increased substantially. The trends, however, differ across the four platform chemicals. In the five years from 2000 to 2004, research on light olefins and aromatics predominated—likely because of the high heterogeneity of and many routes to produce olefins and aromatic compounds. By the period from 2020 to 2024, ammonia research prevailed. The acceleration of research, as illustrated by the increase between the two five-year periods, is the strongest for ammonia, with the number of articles increasing from 316 to 5359 (factor 17). The increase for the other three platform chemicals ranges from ×6 for methanol to ×4 for olefins to ×3.5 for aromatics (*cf.* top part of Fig. 2). In comparison, overall research tripled during the same period (*cf.* Table S4 of the SI), indicating that research on all four groups outpaced the overall growth of scientific research. This pattern can be interpreted to build on a general increase in climate awareness (exemplified by, *e.g.*, the Kyoto protocol 1997) as well as the establishment of green chemistry principles by Anastas and Warner in 1998,^32^ which emphasise atom economy, waste reduction, and the use of renewable feedstocks. As a result, researchers seem to have intensified their focus on developing environmentally benign synthesis routes, catalysis strategies, and bio-based alternatives for traditional petrochemical-derived platform chemicals.^32,62,63^

Several factors could explain the differences in the acceleration of research between the four platform chemicals. First, they differ in molecular complexity, which we define as the number of atoms forming the molecule, and the bonds to be established during synthesis. Assuming nitrogen and hydrogen are input materials, ammonia is the least complex to synthesise. Methanol is slightly more complex, followed by light olefins, which require more elaborate processing. The most complex are aromatic compounds, representing a heterogeneous group with significantly greater synthetic demands. As shown below, developing new synthesis routes based on small and abundant molecules such as CO_2_, H_2_O, or N_2_ has focused considerable efforts.^64^ A lower molecular complexity thus makes the design of such strategies easier by reducing the number of steps and enabling enhanced rationalisation of the investigated process, which could lead to more research focus and output for ammonia. Second, they differ in importance for the mitigation of climate change. Ammonia causes the highest greenhouse gas emissions, followed by olefins, methanol, and aromatics.^65^ This fact raises the urgency for sustainability-focused research for ammonia and (to a slightly lesser extent) for olefins and methanol in accordance with the potential derived from their huge markets.

Third, they differ with respect to product market dynamics. Ammonia and methanol production volumes are likely to grow faster than those for olefins and aromatics, supported by anticipated schemes where these products are central to the operation of society.^13,66^ Among others, the methanol economy paradigm foresees olefins and aromatics to be produced from methanol *via* the technologically advanced methanol-to-olefins (MTO; TRL 8–9) and methanol-to-aromatics (MTA; TRL 7) processes.^67,68^ The vision of defossilised methanol being used as a drop-in feedstock replacement to produce sustainable olefins and aromatics^67^ might have been a driver for the extra research effort in methanol, reducing to some extent research efforts on finding new routes for olefins and aromatics. Likewise, methanol could be used as an energy vector, expanding its use beyond the chemical sector. Furthermore, ammonia has no real alternative in fertiliser production, and its foreseeable application as fuel or as a hydrogen storage material could make it a cornerstone molecule in the future.^69^ On the other hand, fossil-based plastics production—although still predominant—could be replaced by more circular approaches or biogenic materials,^70,71^ leading to greater uncertainty in future olefins demand. The observed increase in ammonia research is particularly noteworthy. It may represent a conservative estimation, since our search excluded research focused exclusively on hydrogen (the key feedstock to ammonia production) in the query (*cf.* section 3.1 of the SI for the reasons).

### Research on platform chemicals focuses more on sustainability aspects

Overall, there seems to be a paradigm shift from improving economics to (also) considering environmental aspects in platform chemicals research.^8^ Beyond the differences in the bare amount of research output, deeper insights emanate from the analysis and comparison of the *direction* of research, which is defined here as the relative share of specific categories or topics in total research (*cf.* the stacked bar charts in Fig. 2). During the first period under analysis (*cf.* 2000–2004 in Fig. 2) and before (*cf.* Fig. S9 of the SI for an analysis from 1980 on), research across all groups primarily focused on improving the economics and energy efficiency of established (mostly thermocatalytic) production routes (category I: process optimisation & energy efficiency). This contrasts with predominant topics in more recent years (*cf.* 2020–2024 in Fig. 2), where there has been a notable increase in research focused on emerging routes such as photo- and electrochemical ones for ammonia, methanol, and, to a certain extent, also for olefins and aromatics.^72–75^ While for ammonia, methanol, and olefins the direction of research has changed towards photo- and electrochemical approaches (as opposed to thermocatalytic approaches; category II), the direction for aromatics research has rather shifted towards the use of alternative feedstocks (category IV). The complex structures of the plethora of aromatic compounds present challenges for alternative production routes and make cracking of longer hydrocarbons the main route in the foreseeable future. In the case of aromatics, this lack of viable, more sustainable alternatives has driven research into using alternative, often biogenic, feedstocks (category IV) to improve the sustainability of existing routes.

### Ammonia and methanol research accelerated after the Paris Agreement

**Fig. 3:**
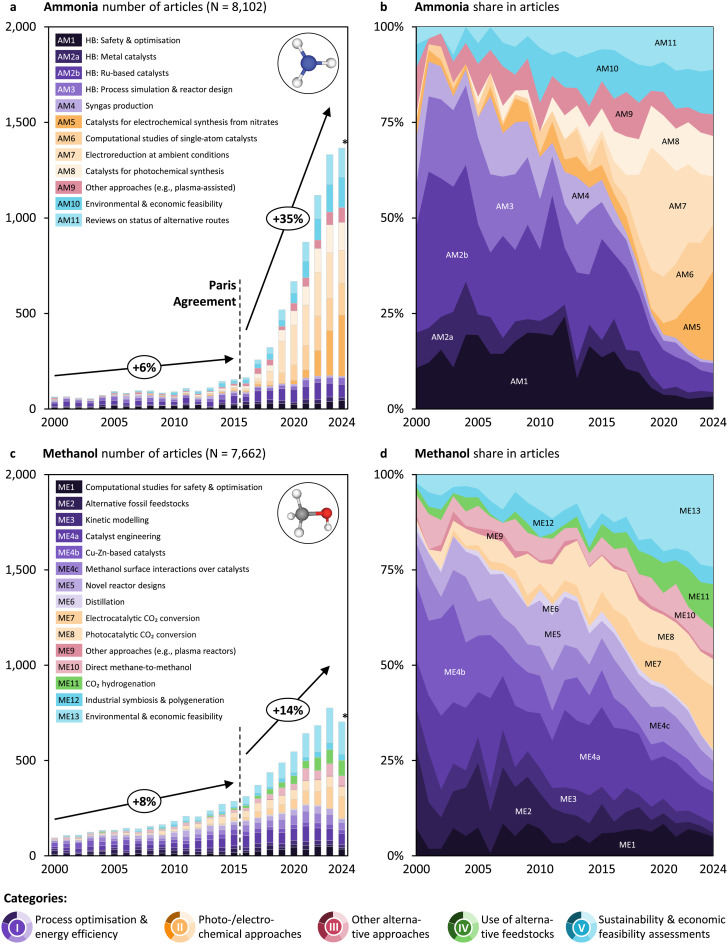
(a) and (c) Number of articles per topic and year for ammonia and methanol, respectively. The *N* values indicate the subset of articles published from 2000 on. The percentage values on the arrows indicate the compound annual growth rate (CAGR) of publications before (2000–2015) and after the Paris Agreement (2015–2023). *For 2024, articles are only considered until a mid-year cut-off date (*cf.* Table S4 of the SI). (b) and (d) Relative share in articles per topic and year for ammonia and methanol, respectively. Abbreviations: HB = Haber–Bosch.

**Fig. 4:**
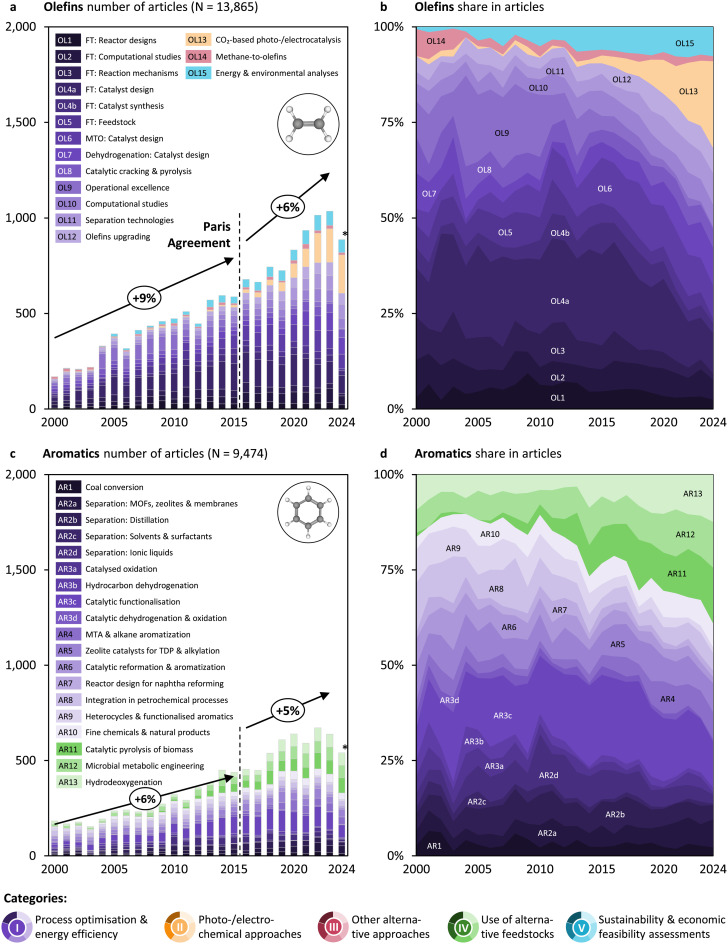
(a) and (c) Number of articles per topic and year for olefins and aromatics, respectively. The *N* values indicate the subset of articles published from 2000 on. The percentage values on the arrows indicate the compound annual growth rate (CAGR) of publications before (2000–2015) and after the Paris Agreement (2015–2023). *For 2024, articles are only considered until a mid-year cut-off date (*cf.* Table S4 of the SI). (b) and (d) Relative share in articles per topic and year for olefins and aromatics, respectively. Abbreviations: FT = Fischer–Tropsch; MOF = metal–organic-framework; MTA = methanol-to-aromatics; MTO = methanol-to-olefins; TDP = toluene disproportionation.

These heterogeneous trends warrant a detailed analysis in yearly resolution to better understand the underlying dynamics and factors driving shifts in research output and direction (*cf.*Fig. 3 and 4). Since about 2015, research output for ammonia and methanol has increased faster than before, suggesting that recent climate commitments like the Paris Agreement in 2015 translate into research. Ammonia research experienced the most significant acceleration, with the compound annual growth rate (CAGR) rising from 6% to 35% (*cf.*Fig. 3a). That for methanol nearly doubled (*cf.*Fig. 3c). Still, olefins and aromatics research seem not to witness an acceleration around 2015 (*cf.*Fig. 4a and c). To put these figures into perspective, overall research in the field grew by 5% before (2000–2015) and also 5% after the Paris Agreement (2016–2023), with ammonia and methanol research expanding above the average, while olefins and aromatics research grew in line with the broader research trends. For all four groups of platform chemicals, research focused on process optimisation and energy efficiency (category I, violet topics) has continued to grow steadily since 2000. However, it is the remarkable growth in other categories that has driven the acceleration of research. These categories, and the topics within them, differ among the four platform chemicals as follows.

### Ammonia research shifts towards photo- and electrochemical approaches

Ammonia has been produced industrially for over a century through the high-pressure (150–350 bar) and high-temperature (400–500 °C) Haber–Bosch (HB) process, which reacts nitrogen separated from the air with hydrogen typically derived from natural gas *via* steam methane reforming.^76^ At the beginning of the 21^st^ century, ammonia research was primarily focused on improving the efficiency of HB, *e.g.*, to reduce pressure and temperature^77^ (*cf.* the violet topics in category I, Fig. 3a and b). Studies concentrated on plant-level optimisation (AM1), catalyst development (AM2a and AM2b), process simulation and reactor design (AM3), and improving syngas production (AM4). While research in these areas is still ongoing, particularly with regard to ruthenium-based catalysts (AM2b), the substantial increase in publications has been primarily driven by research in new photo- and electrochemical approaches (category II, yellow topics), sustainability and economics (category V, blue topics), and yet alternative approaches such as plasma (category III, red topic).

This case illustrates the key role of reproducibility in establishing new routes. Research into electroreduction of nitrogen at ambient conditions and mostly under aqueous electrolytes (AM7) saw an initial surge after 2012, after theoretical^78^ and practical^79,80^ studies suggesting its feasibility, reaching *ca.* one third of the scientific literature on this topic in 2020. The demanding activation of the triple bond in the N_2_ and the kinetically easier reduction of water into H_2_ imposed a considerable challenge in catalyst design, leading to reports on new materials yielding very low ammonia yields, resulting in troublesome product quantification. The milestone article by Andersen *et al*. (2019) almost universally attributed previous ammonia production results to contamination,^81^ and resulted in some retractions,^82,83^ triggering a decrease in relevance that continues today. Research efforts quickly transferred to electrochemical ammonia synthesis from nitrates^84^ (AM5), which aims to synthesise ammonia from, for instance, wastewater under milder conditions compared to HB, and where less formidable kinetic barriers enable high ammonia yields and thus reliable identification of its origin. Additionally, computational studies of single-atom catalysts^85^ (AM6) have grown steadily since 2017, seeking to enhance selectivity and activity in electrocatalytic ammonia synthesis, also progressively shifting from N_2_ to nitrates as the nitrogen source. Photochemical ammonia synthesis^86,87^ (AM8), which promises to replace energy-intensive thermocatalytic HB, also emerged as a new research direction. Despite increased research on plasma^88,89^ and alternative synthesis approaches (*e.g.*, concentrated solar process heat-driven synthesis^90^) (AM9), no single approach has yet gained widespread traction.^91^

The growing interest in the sustainability of ammonia production^5,69,92–97^ (AM10) highlights the increasing recognition of ammonia's potential as a key platform chemical and fuel in a more sustainable economy and, more generally, the need to integrate systems engineering studies to reinforce and guide the development of new catalysts.^98^ Finally, this rapid increase in the scientific production and diversity has naturally led to a rising number of studies reviewing the progress and feasibility of various alternative production routes^99,100^ (AM11). Notably, today's energy models seem to disregard trends around electro- and photochemical approaches and typically only compare fossil and renewable HB, arguing that electrochemical routes are neither technologically ready nor cost-competitive.^4,11,53^

The expert discussion of recent reviews and reference articles on sustainable ammonia production suggests common challenges. Chief among these is the heavy energy demand and CO_2_ emissions of the traditional HB process, which requires major advances in cost-effective, large-scale green hydrogen *via* renewables and water electrolysis to achieve decarbonisation (category II).^101,102^ The community agrees about the fact that high costs, limited scalability, and the need for significant infrastructure overhaul remain barriers to immediate industrial adoption (category V).^103,104^ Reliable access to pure water and integration with intermittent renewable power are further technical and logistical challenges. Recent reviews highlight that emerging alternatives, such as electrochemical and plasma-based synthesis, currently face low efficiency and poor catalyst stability (categories II and III).^105,106^

Overall, to transition toward sustainable ammonia production, the identified tendencies suggest two key directions to be prioritized: the design of advanced catalysts for alternative feedstocks (category IV) and the full electrification of the synthesis process (category II). Future efforts could focus on developing catalytic systems capable of activating unconventional nitrogen (*e.g.*, nitrates) and hydrogen sources (*e.g.*, water or biogas) under electrocatalytic or mild or plasma-assisted thermocatalytic conditions, reducing energy intensity and enhancing process flexibility. Simultaneously, using renewable energy not only in the core ammonia synthesis reaction but also in other parts of the plant, *e.g.*, gas compression, could reduce carbon emissions further across the entire value chain. These directions align with long-term technological roadmaps and life-cycle analyses that emphasize renewable hydrogen, electrochemical conversion routes, and modular plant design as the foundation of a sustainable ammonia economy.^13,107,108^

### Methanol research diversifies increasingly

Methanol production has been industrialised for over a century through the catalytic hydrogenation of fossil carbon monoxide and hydrogen (syngas),^109^ which is commonly derived from natural gas *via* steam reforming or coal gasification. Despite improvements with the introduction of modern copper-based catalysts in the late 20^th^ century, the process still demands high-pressure (50–100 bar) and relatively high temperatures (200–300 °C). Notably, even though small amounts of CO_2_ are added to the syngas feed stream to enhance productivity,^110^ this process stays among the largest net CO_2_ emitters in the chemical industry.^46^ In the early 2000s, methanol research was predominantly focused on improving the efficiency of catalytic hydrogenation of fossil feedstocks (*cf.* the violet topics in category I in Fig. 3c and d). Studies focused on computational modelling (ME1), the exploration of alternative fossil feedstocks (ME2), kinetic modelling (ME3), catalyst engineering (ME4a), and innovations in reactor design, such as membrane reactors^111,112^ (ME5). Research on distillation processes (ME6) also continued to play a significant role. While research in these areas persists and often forms the basis for advances in other categories, the increase in publications since 2015 is primarily attributed to other research categories, including system-level studies on sustainability and economics (category V, blue topics), photo- and electrochemical approaches (category II, yellow topics), and the investigation of CO_2_ as an alternative feedstock (category IV, green topics).

The methanol economy concept, coined by Olah in 2005,^66^ methanol's liquid, transport-friendly nature, making it convenient for transportation, and its versatility to be transformed into olefins^68^ and aromatics,^113^ resonated with the increasing awareness in sustainability. It may have triggered the ongoing search for defossilised routes. Research on electrocatalytic^114–116^ (ME7) and photocatalytic CO_2_ conversion^117–120^ (ME8) has emerged as a key area of interest, as these routes offer the potential to synthesise methanol exclusively from CO_2_ and under ambient conditions. The thermocatalytic arena has also undergone significant advancement due to the insufficient selectivity and stability of the traditional Cu–Zn catalysts^121,122^ under CO_2_-rich streams. Novel catalysts such as indium oxide-based^123–125^ or zinc–zirconia-based^126,127^ (ME11) now offer very high selectivity to methanol with promising stability. The challenging one-step partial oxidation of methane into methanol^128–132^ (ME10) aims to eliminate the need for the production of the precursor syngas and has also generated considerable attention, but still faces severe selectivity issues to avoid overoxidation into CO_*x*_ or formaldehyde.

Research into alternative approaches, such as plasma^133,134^ or slurry-phase reactors^135^ (ME9), has also been explored, albeit on a smaller scale. Another consistent area of research has been the optimisation of methanol production within the context of industrial symbiosis and polygeneration^136–138^ (ME12), which also includes research on carbon capture and utilisation (CCU). Methanol production's economic and environmental feasibility, including its system-level implications, has gained increasing attention over the years, again driven by George Olah's idea of the methanol economy.^66^ This research stream, focused on the broader environmental and economic feasibility of methanol^5,6,139–146^ (ME13), has been gaining momentum until today, representing the largest share of publications in 2024 in anticipation of the number of industrial projects announced developing sustainable methanol production over the coming years.^147^

Our analysis of recent review articles reveals that the literature agrees on certain challenges that need to be tackled. A prominent one is the high capital and operational costs for green hydrogen and CO_2_-based routes.^148,149^ Catalyst activity and stability remain important factors for efficient and selective conversion across different strategies, with thermocatalytic ones displaying the more advantageous performance (categories II, III, and IV).^148,150^ Solving integration issues among renewable electricity, hydrogen generation, and methanol synthesis is thought to reduce overall process efficiency and scale-up potential (category V).^149,151^ Lastly, regulatory uncertainty and high cost of alternative routes hamper investment and commercialisation.^148^

Overall, methanol (similarly to ammonia) witnesses a stark shift in research from incremental towards more transformative pathways. In particular, the increase in research on photo- and electrochemical research appears promising as it could eventually lead to a true shift in industrial production down the road (category II). Building on this momentum, future research could prioritize developing advanced catalytic materials tailored for efficient conversion of alternative feedstocks such as CO_2_, biogas, and methane, with a focus on enhancing selectivity, activity, and long-term stability under mild conditions (category IV). Among these, we think additional research efforts on CO_2_ hydrogenation pathways are likely to bring benefit in the short- and mid-term. Additionally, emphasis on novel electrocatalysts and photocatalysts capable of integration with renewable electricity are likely crucial to enable fully electrified “power-to-methanol” processes in the mid- to long term.

### New routes emerge in the mature field of olefin production

Light olefins have a long-standing industrial history and have become central to plastics production. A turning point in their industrialisation marked the discovery of transition metal catalysts (based on titanium and aluminium compounds) by Ziegler and Natta in the 1950s. Another 50 years later, the society is plastics-centred, with polyolefins (polypropylene and polyethylene) accounting for *ca.* 60% of total plastic production.^152^ Today, olefins are produced *via* steam cracking or fluid catalytic cracking of long-chain hydrocarbons like naphtha or natural gas liquids at high temperatures (750–900 °C) but moderate pressure levels (1–5 bar). In the early 2000s, olefins research was predominantly focused on improving the efficiency of the incumbent production routes (*ca.* 90% of research contributed to the violet topics in category I in Fig. 4a and b). Many studies focused on improving the Fischer–Tropsch process—they focus on reactor designs (OL1), computational studies (OL2), reaction mechanisms (OL3), catalyst design (OL4a), catalyst synthesis (OL4b), and feedstocks (OL5). Beyond Fischer–Tropsch, studies focused on catalyst design for methanol-to-olefins (MTO; OL6), catalyst design for dehydrogenation (OL7), catalytic cracking and pyrolysis (OL8), operational excellence (OL9), computational studies (OL10), separation technologies (OL11), and olefins upgrading (OL12).

Only three topics were identified for pursuing a new ecosystem. After attracting considerable interest between the 1980s^153^ and early 2000s (up to 20%, *cf.* Fig. S9), studies on methane-to-olefins^154–156^ (OL14) contribute only a small share of overall research today, as decades of research have failed to solve challenges in catalyst design. Research on photo- and electrocatalytic synthesis based on CO_2_, however, increased after ∼2015 above average^157–160^ (OL13), enabled through research by Hori *et al*. (1985), first showing the electrochemical reduction of CO_2_ to ethylene over copper catalysts.^161^ It has not been commercialised yet, among other reasons, due to stability and selectivity not matching the stringent requirements to compete against incumbent processes.^162^ The number of published energy and environmental analyses^163^ (OL15) has been growing in line with the overall research on olefins production, pointing to the general trend of increased awareness about the need for sustainability assessment to inform decision-making and guide research.^8^

Our expert analysis of recent review and influential articles reveals several persistent barriers in sustainable olefin production. Catalyst selectivity and stability remain major issues, especially in CO_2_ hydrogenation and tandem systems, where undesired by-products and deactivation limit performance (categories II, III, and IV).^164,165^ Economic viability is constrained by high energy demand and capital costs, particularly in multistep routes like MTO, which must increase its TRL.^164,166^ Environmental trade-offs, including indirect emissions and resource burdens, arise unless powered by low-carbon energy (category V).^167,168^ In addition, feedstock variability and the lack of scalable supply chains for CO_2_, biomass, or plastic waste may hinder consistent operation.^165,166^

Building on the ongoing shift toward transformative approaches in olefins production, future research could emphasize the design of innovative catalytic materials that enhance selectivity and stability for abundant feedstocks such as CO_2_ and methane (categories II and IV). Given the persistent challenges in methane-to-olefins conversion, breakthroughs in catalyst development appear crucial to overcome issues related to activity and product distribution. Likewise, advancing electrocatalytic and photocatalytic systems for CO_2_ reduction to olefins holds promise but requires focused and maintained efforts to improve catalyst durability and selectivity under operating conditions. Combining these catalyst innovations with process electrification and reactor design enhancements will likely be key to integrating renewable energy and enabling low-temperature, low-pressure olefin synthesis.

### The structure of research in aromatics seems to endure

Aromatics have been produced industrially for decades, primarily through catalytic reforming of petroleum naphtha at high temperatures (450–520 °C) and moderate pressures (10–35 bar). A secondary source of aromatics is steam cracking, where aromatics are co-produced alongside olefins. A major advancement in aromatics production came in the latter half of the 20^th^ century with the development of toluene disproportionation (TDP), which operates at 400–500 °C and 20–30 bar and enables the conversion of toluene into higher-value benzene and xylenes. Today, aromatics such as benzene, toluene, and xylenes (BTX) remain essential building blocks for the chemical industry, feeding into the production of polymers, solvents, and synthetic fibres. In the early 2000s, aromatics research was largely focused on improving the efficiency of the incumbent production routes (*ca.* 75% of research contributed to the violet topics in category I in Fig. 4c and d). Studies focused on coal conversion (AR1), new separation approaches *via* metal organic frameworks (MOFs), zeolites, and membranes (AR2a), separation *via* distillation (AR2b), solvents and surfactants for separation processes (AR2c), and ionic liquids used for separation (AR2d).

Another set of topics focused on catalysis, studying catalysed oxidation (AR3a), hydrocarbon dehydrogenation (AR3b), catalytic functionalisation (AR3c), and catalytic dehydrogenation and oxidation (AR3d). Articles in yet other topics study methanol-to-aromatics (MTA) and alkane aromatisation (AR4), zeolite catalysts for toluene disproportionation (TDP) and alkylation (AR5), catalytic reformation and aromatisation (AR6), reactor design for naphtha reforming (AR7), and integration in the petrochemical processes (AR8). Yet other studies focused on the production of heterocycles and functionalised aromatics (AR9) and fine chemicals and natural products (AR10). These topics were included despite them not studying exclusively the production of aromatic building blocks but also of downstream products, as drawing the lines for aromatic compounds proved difficult. However, studies on the use of alternative feedstocks increased over the study period—from *ca.* 12% for 2000–2004 to 33% for 2020–2024. Within this category, studies on catalytic pyrolysis of biomass^25,152,169–175^ (AR11), microbial metabolic engineering^176–179^ (AR12), and hydrodeoxygenation^180–184^ (AR13) all grow, with AR11 growing the fastest. While articles included in AR12 do not focus on the production of aromatic building blocks, they study the production of specific aromatic compounds through pathways that would make the production of the building blocks unnecessary.

Notably, for aromatics, the number of identified topics is largest and the amount of research on separation (AR2a–d, *ca.* 20%) is significantly greater than for the other three chemicals (<5% for ME6 and <10% for OL11), which is likely due to the variety of compounds produced. Furthermore, no topics in category II (photo-/electrochemical approaches) were identified for aromatics. This is likely due to the high molecular complexity of aromatic molecules, making it extraordinarily difficult to design catalytic processes. Overall, research still targeting efficiency and process improvements for olefins and aromatics could be explained in two ways. Either research agendas are short-sighted and industry-driven, or they simply rely on “clean drop-in feedstocks” being available in the long term to make production more sustainable. Research and industry could rightfully challenge research on complicated and potentially not particularly promising routes for aromatics production, given that MTA is already being demonstrated.^113^ Surprisingly, although growing, research on MTA (AR4) today still makes up less than 10% of the analysed aromatics research.

Our analysis of recent review articles, finally, suggests several important challenges of renewable aromatic production. First, the complexity and variability of biomass and waste feedstocks make consistent processing difficult, requiring effective pretreatment and purification to ensure reliable product yields (category IV).^185,186^ Second, catalyst development remains critical, as catalysts must combine high activity, selectivity, and durability under harsh reaction conditions such as depolymerization or pyrolysis while dealing with complex feedstocks.^187,188^ Third, the complexity of controlling reaction networks limits efficient scale-up and integration, posing hurdles to process optimization and energy use (category V).^188^ Fourth, environmental sustainability must be thoroughly evaluated, including feedstock sourcing, life cycle emissions, and handling of by-products to ensure actual ecological benefits.^186,188^ Finally, the economic viability of renewable aromatic technologies is challenged by operational costs and early technology readiness.^186^

Building on current research trends, future directions for sustainable aromatics production could focus on the design of novel catalytic materials capable of efficiently converting alternative feedstocks such as biomass-derived intermediates through catalytic pyrolysis and hydrodeoxygenation, which currently show the fastest growth in research attention (category IV). Given the high molecular complexity of aromatics, catalyst development should aim to improve selectivity and stability while enabling functionalization under milder conditions to circumvent the energy-intensive traditional reforming processes. Although photo- and electrochemical approaches remain underexplored due to these challenges, integrating advanced catalysis with emerging reactor designs and process intensification could facilitate more sustainable pathways (category II). Additionally, optimizing separation technologies remains critical given the diverse aromatic product mix (category V). Overall, advancing MTA technologies holds promise and warrants intensified focus to accelerate commercialization and reduce reliance on fossil-based routes.

### Research directions differ geographically

**Fig. 5:**
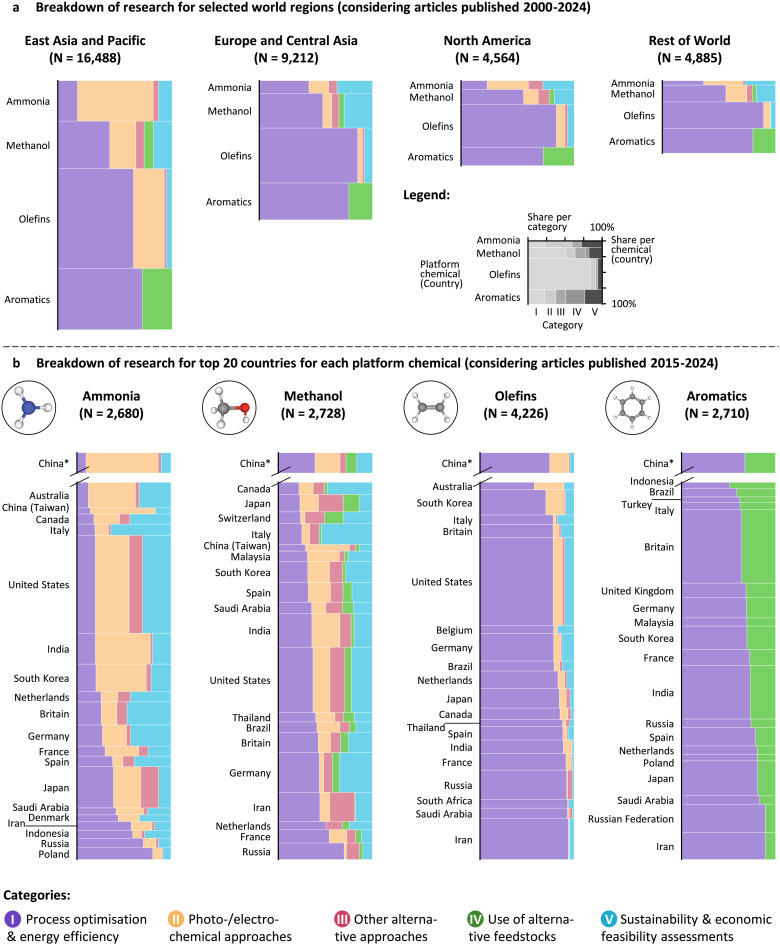
(a) Breakdown of research for selected world regions (showing articles published between 2000 and 2024). (b) Breakdown of research for the top 20 countries for each platform chemical, sorted by contribution of category I (measured in the absolute number of articles between 2015 and 2024). *Articles published in China are shown on separate *y*-axes as they exceed those published in the United States (the country with the second most articles for all platform chemicals) by a factor of 3 to 5.

When examining research trends across major geographical regions, several key observations emerge. As shown in Fig. 5a, there is a noticeable discrepancy in the number of articles published in different world regions. Specifically, researchers in East Asia and the Pacific region published nearly twice as many articles as those in Europe and Central Asia (the second most contributing region) and nearly four times the number of articles in North America (the third most contributing region). This trend is primarily driven by China, which contributed over one-third of all publications in this period (in line with general shifts in the global research landscape). Thus, the East Asia and Pacific region accounts for roughly half of the global research output. Across all regions, research on olefins and aromatics still represents the largest share of publications. This is likely due to the heterogeneous nature of these two chemical groups, in contrast to ammonia and methanol, which are represented by a single chemical compound each. Research on olefins and aromatics involves diverse processes, feedstocks, and product streams, contributing to the high volume of publications in these categories. The discussed acceleration of research on ammonia and methanol happened too recently to change the overall picture in any of the regions.

In East Asia and the Pacific, there is a marked increase in research outside of mainstream^189^ process optimisation and energy efficiency (category I, violet). Notably, this region's research on ammonia and olefins has focused significantly on more risky^189^ photo- and electrochemical approaches (category II, yellow), while methanol research spans all four categories. For aromatics, however, there is a strong emphasis on the use of alternative feedstocks (category IV, green). In contrast, Europe and Central Asia have a stronger focus on sustainability and economic feasibility assessments (category V, blue) for ammonia, methanol, and aromatics, which could be a symptom of the urgency to quantify and prove green technologies through economic and environmental metrics. Overall, however, Europe and Central Asia still show a greater focus on mainstream process optimisation and energy efficiency, with risky photo- and electrochemical research being less pronounced. This trend is also observed in North America and the Rest of the World, where research on photo- and electrochemical methods is slightly more prominent than in Europe but still follows a pattern similar to that of Europe and Central Asia. These differences in the type of research—mainstream or risky—could potentially go back to different strategies in allocating research grants.^189–191^

Looking at trends on a country level provides a more nuanced perspective. Fig. 5b reveals that differences across countries are more pronounced for ammonia and methanol than for olefins and aromatics. In line with expectations, countries like Poland, Russia, Indonesia, Iran, and Saudi Arabia—nations with a history of cheap and abundant natural gas—show high shares of research in category I, focusing primarily on improving processes and energy efficiency. On the other hand, countries that have historically relied on importing natural gas, such as South Korea, India, and China, exhibit lower shares of category I research. This indicates a shift in the focus of research efforts, particularly in regions aiming to reduce their dependency on fossil fuel imports. Countries with a high biomass potential like Indonesia or Brazil seem to also focus on alternative (potentially biogenic) feedstocks (category IV, *cf.* aromatics), while those with a high solar potential like Australia or India seem to focus more on photo- and electrochemical approaches (category II, *cf.* ammonia and methanol). Overall, funding agencies seem to generally align research agendas with such political priorities.

### China is a prime example of the shift away from fossil feedstocks

One of the most striking shifts in research trends comes from China, which is working towards reducing emissions from often coal-based ammonia production^192^ and reducing its reliance on fossil fuel imports. As a case in point, China prohibited fertiliser exports to safeguard its domestic supply of ammonia.^193^ We find a clear shift in the research in China towards more transformative pathways. For ammonia, three-quarters of China's research is now dedicated to photo- and electrochemical approaches, while for methanol, more than a quarter is devoted to these methods. This shift indicates China's push toward electricity-based production of platform chemicals, positioning the country as the leader in research output on green chemical production. Our dataset allows us to trace much of this research to specific grants from the National Natural Science Foundation of China (NSFC), suggesting a national effort to support and accelerate the risky development^189^ of alternative, sustainable methods for platform chemical production. The rapid advancements in China's research and development of electricity-based platform chemicals have far-reaching implications. Compared to China, research in other countries seems to shift more slowly.

### Academic trends *versus* industrial reality in chemical innovation

We believe that academic publication trends provide a valuable window into where scientific inquiry is currently focused, but like to emphasize that they do not necessarily translate to immediate changes in industrial practice. Frequently, a surge of academic work signals an early, exploratory stage for a technology, after which the spotlight tends to shift toward practical engineering and scale-up, often resulting in less visibility in the academic literature. A classic example is the HB process: it was once at the forefront of scientific research, but today, its advancements largely take place behind the scenes in applied engineering rather than in scholarly journals, as reflected by trends in Fig. 3b.

In contrast, areas like electrocatalytic reduction of nitrogen-containing compounds and carbon dioxide are now the subject of significant academic attention, despite still being a long way from full technical maturity. At the same time, established thermocatalytic methods such as the conversion of CO_2_ to methanol or the transformation of methanol to olefins and aromatics are poised to play a more immediate role in defossilisation, given their comparatively advanced state of development and alignment with existing industrial infrastructure.

It should also be recognized that published academic research only represents the visible fraction of the overall landscape of innovation. It is often the case that industrial development occurs through proprietary research and is typically only revealed to the public *via* patents. Accordingly, a comprehensive patent analysis could serve as a complement to our analysis of academic literature, offering further insights into industrial goals and directions. The familiar image of the iceberg—where most of its mass lies unseen below the surface—aptly describes this dynamic. For instance, DuPont's decade-long work on nylon remained largely secret until the relevant patent was filed,^194^ underscoring how transformative developments can occur outside the public academic record.

## Conclusions and outlook

Overall, research on sustainable platform chemicals has grown at a faster rate than the overall scientific literature. Over the last 25 years, during which the concept of Green Chemistry has brought environmental concerns as a primary focus, there has been a significant evolution in research, shifting from process optimisation and energy efficiency towards other routes enabling larger quantitative gains in the sustainability of producing these compounds. This shift is driven by the increasing need to transition the industry from fossil to renewable feedstocks and clean electricity to attain net-zero emissions. Notably, ammonia synthesis has seen the most significant growth in research, particularly after the Paris Agreement of 2015, reflecting its central role in the global push for more sustainable chemical production. The transition from efficiency-driven research to novel production routes is more pronounced for ammonia and methanol, which evolve towards processes that are based on electricity and alternative feedstocks. In contrast, olefins and aromatics production may achieve sustainability through clean drop-in feedstocks, making the shift less pronounced in these sectors. China is pivotal in this transformation, particularly its shift from thermocatalytic towards photo- and electrocatalytic approaches. This development signals an important trend in the field and merits attention from researchers, industry stakeholders, and policymakers. Given that researchers in Europe and the United States have a stronger focus on techno-economic and life-cycle assessments (*cf.* category V in Fig. 5b), it seems important to include this shift towards photo- and electrochemical research in these assessments.

The combination of global research trends (*e.g.*, towards photo- and electrochemical approaches) and geographical focal points allows researchers and funding agencies to identify promising research areas and (locally) underexplored topics. At the same time, “acknowledging the current unsustainability of the chemical industry”^8^ allows to identify and highlight research not fully aligned with long-term policy targets but rather serving locked-in, short-term industry agendas.^49,195^ This opens the debate of how to optimally allocate resources, considering that certain research topics could be financed through industry initiatives or jointly with public funds, freeing up public grant money for more long-term oriented research. Moreover, the broad potential socio-economic and environmental repercussions of the identified selected trends should be carefully evaluated by governments and industry when delineating policies and strategic plans. This would, in turn, require further research on quantitative assessments of such technologies. For example, the shift towards alternative feedstocks and electricity for platform chemicals could significantly increase demand for sustainable biomass and electricity. Platform chemicals could become a major driver of surplus electricity demand, requiring careful consideration by policymakers. This will necessitate the establishment of clear renewables targets, which consider the electricity demand from sustainable platform chemical production,^5,11,196^ and an investment environment that facilitates capacity expansion. In the case of biomass, competition between platform chemical production and other industries, such as cement, biofuels, and bioenergy with carbon capture and storage (BECCS),^197^ underscores the need for national and supranational biomass prioritisation strategies. Policymakers must act to prevent misallocation of resources due to a lack of coordination across sectors. For large petrochemical companies, the long-term viability of platform chemical production will require rethinking localisation strategies. While cheap fossil feedstocks have historically been a key factor in location decisions, the growing importance of clean electricity may soon become a central criterion for future investments.^11,196^ At the same time, transformative pathways could lead to a stranding of traditional production assets, demanding a rethinking of plant setups and integration strategies,^198,199^ including repurposing fossil infrastructure. Accordingly, industry could also use our results to assess how transformative changes can be integrated into the current production infrastructure to reduce entrance barriers and boost their performance.

Deriving these insights and implications is only possible through an advanced methodology, like the one presented, that integrates probabilistic topic modelling, generative AI, and expert input to uncover latent structures in text data and quantify research trends. Notably, we believe that our approach – combining generative AI with expert input – could be applied for uncovering research trends in other fields as well, improving the holistic understanding of innovation patterns despite exploding publication volumes. While such methods are increasingly applied in the climate policy sphere,^200–202^ this appears to be less the case in some engineering and hard science fields. Complementing our analysis with an analysis of the quality of research would even improve our understanding of the identified research trends. For instance, one could use citation data to uncover knowledge networks, article centrality, and spillovers^203^ to gain further insights into the importance of topics and global research dynamics, acknowledging the limitations of using any quantitative metric as a proxy of quality. Looking ahead, aligning decision-making in research, industry, and policy innovation theory can effectively steer innovation for platform chemical production, leading to a more sustainable chemicals industry.

## Author contributions

PT: conceptualization, methodology, software, validation, formal analysis, investigation, data curation, writing – original draft, writing – review & editing, visualization, project administration, funding acquisition. BT: methodology, software, validation, formal analysis, investigation, data curation, writing – original draft, writing – review & editing. AJM & LFS: conceptualization, methodology, formal analysis, investigation, writing – original draft, writing – review & editing, visualization. GGG & JPR: conceptualization, writing – review & editing, supervision, funding acquisition. BS: conceptualization, writing – review & editing, supervision, funding acquisition.

## Conflicts of interest

There are no conflicts of interest to declare.

## Supplementary Material

GC-027-D5GC02863A-s001

## Data Availability

The data supporting this article have been included as part of the supplementary information (SI). See DOI: https://doi.org/10.1039/d5gc02863a.

## References

[cit1] Smith J. K. , in Chemical Sciences in the Modern World, ed. S. H. Mauskopf, University of Pennsylvania Press, Philadelphia, 1994, pp. 137–157

[cit2] Lancaster M. , in Green Chemistry: An Introductory Text, The Royal Society of Chemistry, 2016

[cit3] Meng F., Wagner A., Kremer A. B., Kanazawa D., Leung J. J., Goult P., Guan M., Herrmann S., Speelman E., Sauter P., Lingeswaran S., Stuchtey M. M., Hansen K., Masanet E., Serrenho A. C., Ishii N., Kikuchi Y., Cullen J. M. (2023). Proc. Natl. Acad. Sci. U. S. A..

[cit4] Gabrielli P., Rosa L., Gazzani M., Meys R., Bardow A., Mazzotti M., Sansavini G. (2023). One Earth.

[cit5] Nabera A., Martín A. J., Istrate R., Pérez-Ramírez J., Guillén-Gosálbez G. (2024). Green Chem..

[cit6] Gabrielli P., Gazzani M., Mazzotti M. (2020). Ind. Eng. Chem. Res..

[cit7] Cespi D. , Jourdan L., Jonckheere F., Geffarth U. and Busson J., in Ullmann's Encyclopedia of Industrial Chemistry, Wiley, 2024, pp. 1–33

[cit8] Mitchell S., Martín A. J., Guillén-Gosálbez G., Pérez-Ramírez J. (2024). Angew. Chem., Int. Ed..

[cit9] Levi P. G., Cullen J. M. (2018). Environ. Sci. Technol..

[cit10] Torrente-Murciano L., Dunn J. B., Christofides P. D., Keasling J. D., Glotzer S. C., Lee S. Y., Van Geem K. M., Tom J., He G. (2024). Nat. Chem. Eng..

[cit11] SYSTEMIQ, 2022, https://www.systemiq.earth/wp-content/uploads/2022/09/Main-report-v1.20-2.pdf

[cit12] The Royal Society , Green Ammonia: Policy briefing, The Royal Society, London, United Kingdom, 2020, https://royalsociety.org/-/media/policy/projects/green-ammonia/green-ammonia-policy-briefing.pdf

[cit13] MacFarlane D. R., Cherepanov P. V., Choi J., Suryanto B. H. R., Hodgetts R. Y., Bakker J. M., Ferrero Vallana F. M., Simonov A. N. (2020). Joule.

[cit14] Valera-Medina A., Xiao H., Owen-Jones M., David W. I. F., Bowen P. J. (2018). Prog. Energy Combust. Sci..

[cit15] Johnson N., Liebreich M., Kammen D. M., Ekins P., McKenna R., Staffell I. (2025). Nat. Rev. Clean Technol..

[cit16] Bell T. E., Torrente-Murciano L. (2016). Top. Catal..

[cit17] Tabanelli T., Cespi D., Cucciniello R. (2021). Catalysts.

[cit18] Schäppi R., Rutz D., Dähler F., Muroyama A., Haueter P., Lilliestam J., Patt A., Furler P., Steinfeld A. (2022). Nature.

[cit19] Gray N., McDonagh S., O'Shea R., Smyth B., Murphy J. D. (2021). Adv. Appl. Energy.

[cit20] McKinlay C. J., Turnock S. R., Hudson D. A. (2021). Int. J. Hydrogen Energy.

[cit21] Lamichhane P., Pourali N., Scott L., Tran N. N., Lin L., Gelonch M. E., Rebrov E. V., Hessel V. (2024). Renewable Sustainable Energy Rev..

[cit22] Chauhan R., Sartape R., Minocha N., Goyal I., Singh M. R. (2023). Energy Fuels.

[cit23] Cui Y., Deng C., Fan L., Qiu Y., Zhao L. (2023). Green Chem..

[cit24] Morschbacker A. (2009). Polym. Rev..

[cit25] Carlson T. R., Cheng Y. T., Jae J., Huber G. W. (2011). Energy Environ. Sci..

[cit26] Hillmyer M. A. (2017). Science.

[cit27] Bozell J. J., Petersen G. R. (2010). Green Chem..

[cit28] Ritzen L., Sprecher B., Bakker C., Balkenende R. (2023). Resour., Conserv. Recycl..

[cit29] Bakker C. and Balkenende R., Mater. Exp. 2 Expand. Territ. Mater. Des., 2021, pp. 193–206

[cit30] Meys R., Kätelhön A., Bachmann M., Winter B., Zibunas C., Suh S., Bardow A. (2021). Science.

[cit31] Neves A., Godina R., Azevedo S. G., Matias J. C. O. (2020). J. Cleaner Prod..

[cit32] Anastas P. T. and Warner J., Green Chemistry: Theory and Practice, Oxford University Press, 1998

[cit33] Handbook on Life Cycle Assessment, ed. H. de Bruijn, R. van Duin, M. A. J. Huijbregts, J. B. Guinee, M. Gorree, R. Heijungs, G. Huppes, R. Kleijn, A. de Koning, L. van Oers, A. Wegener Sleeswijk, S. Suh and H. A. and U. de Haes, Springer Netherlands, Dordrecht, 2002, vol. 7

[cit34] Huber G. W., Iborra S., Corma A. (2006). Chem. Rev..

[cit35] Tautorat P., Lalin B., Schmidt T. S., Steffen B. (2023). J. Cleaner Prod..

[cit36] Leitner W. (2024). Philos. Trans. R. Soc., A.

[cit37] Saygin D., Gielen D. (2021). Energies.

[cit38] Bordet A., Leitner W. (2023). Angew. Chem., Int. Ed..

[cit39] Paths of Innovation: Technological Change in 20th-Century America, ed. D. C. Mowery and N. Rosenberg, Cambridge University Press, Cambridge, 1998, pp. 71–102

[cit40] Rosenberg N. , Inside the Black Box: Technology and Economics, Cambridge University Press, Cambridge, 1983

[cit41] Kline S. J. and Rosenberg N., Stud. Sci. Innov. Process, 2009, pp. 173–203

[cit42] Dosi G. (1982). Resour. Policy.

[cit43] Balconi M., Brusoni S., Orsenigo L. (2010). Resour. Policy.

[cit44] Kline S. J. and Rosenberg N., in The Positive Sum Strategy, National Academies Press, Washington DC., 1986

[cit45] Lucas E., Martín A. J., Mitchell S., Nabera A., Santos L. F., Pérez-Ramírez J., Guillén-Gosálbez G. (2024). Green Chem..

[cit46] Galán-Martín Á., Tulus V., Díaz I., Pozo C., Pérez-Ramírez J., Guillén-Gosálbez G. (2021). One Earth.

[cit47] Artz J., Müller T. E., Thenert K., Kleinekorte J., Meys R., Sternberg A., Bardow A., Leitner W. (2018). Chem. Rev..

[cit48] Malhotra A., Schmidt T. S. (2020). Joule.

[cit49] Schmidt T. S., Battke B., Grosspietsch D., Hoffmann V. H. (2016). Resour. Policy.

[cit50] Nemet G. F. (2009). Resour. Policy.

[cit51] Directorate-General for Mobility and Transport, *European Commission*, 2023, https://transport.ec.europa.eu/transport-modes/air/environment/refueleu-aviation_en

[cit52] Braun M., Grimme W., Oesingmann K. (2024). J. Air Transp. Manage..

[cit53] Neuwirth M., Fleiter T., Hofmann R. (2024). Energy Convers. Manage..

[cit54] Neuwirth M., Fleiter T., Manz P., Hofmann R. (2022). Energy Convers. Manage..

[cit55] Kononova O., He T., Huo H., Trewartha A., Olivetti E. A., Ceder G. (2021). iScience.

[cit56] Callaghan M. W., Minx J. C., Forster P. M. (2020). Nat. Clim. Change.

[cit57] Lu J., Nemet G. F. (2020). Environ. Res. Lett..

[cit58] Blei D. M., Ng A. Y., Jordan M. I. (2003). J. Mach. Learn. Res..

[cit59] Griffiths T. L., Steyvers M. (2004). Proc. Natl. Acad. Sci. U. S. A..

[cit60] Auld G., Mallett A., Burlica B., Nolan-Poupart F., Slater R. (2014). Globe. Environ. Change.

[cit61] Hansen H. F., Rieper O. (2009). Evaluation.

[cit62] Winterton N. (2021). Clean Technol. Environ. Policy.

[cit63] Sheldon R. A. (2017). Green Chem..

[cit64] Martín A. J., Pérez-Ramírez J. (2019). Joule.

[cit65] DECHEMA Gesellschaft für Chemische Technik und Biotechnologie E.V. , Chemical Industry Roadmap for Energy and Resource Efficiency, DECHEMA, Frankfurt am Main, Germany, 2013

[cit66] Olah G. A. (2005). Angew. Chem., Int. Ed..

[cit67] Lopez G., Keiner D., Fasihi M., Koiranen T., Breyer C. (2023). Energy Environ. Sci..

[cit68] Tian P., Wei Y., Ye M., Liu Z. (2015). ACS Catal..

[cit69] Valera-Medina A., Amer-Hatem F., Azad A. K., Dedoussi I. C., De Joannon M., Fernandes R. X., Glarborg P., Hashemi H., He X., Mashruk S., McGowan J., Mounaim-Rouselle C., Ortiz-Prado A., Ortiz-Valera A., Rossetti I., Shu B., Yehia M., Xiao H., Costa M. (2021). Energy Fuels.

[cit70] Vollmer I., Jenks M. J. F., Roelands M. C. P., White R. J., van Harmelen T., de Wild P., van der Laan G. P., Meirer F., Keurentjes J. T. F., Weckhuysen B. M. (2020). Angew. Chem., Int. Ed..

[cit71] Bauer F., Nielsen T. D., Nilsson L. J., Palm E., Ericsson K., Fråne A., Cullen J. (2022). One Earth.

[cit72] Nitopi S., Bertheussen E., Scott S. B., Liu X., Engstfeld A. K., Horch S., Seger B., Stephens I. E. L., Chan K., Hahn C., Nørskov J. K., Jaramillo T. F., Chorkendorff I. (2019). Chem. Rev..

[cit73] Mateo D., Sousa A., Zakharzhevskii M., Gascon J. (2024). Green Chem..

[cit74] Martín A. J., Shinagawa T., Pérez-Ramírez J. (2019). Chem.

[cit75] Xiao Z., Li P., Zhang H., Zhang S., Tan X., Ye F., Gu J., Zou J.-J., Wang D. (2024). Fuel.

[cit76] Liu H. (2014). Chin. J. Catal..

[cit77] Humphreys J., Lan R., Tao S. (2021). Adv. Energy Sustainability Res..

[cit78] Skúlason E., Bligaard T., Gudmundsdóttir S., Studt F., Rossmeisl J., Abild-Pedersen F., Vegge T., Jónsson H., Nørskov J. K. (2011). Phys. Chem. Chem. Phys..

[cit79] Qin Q., Heil T., Antonietti M., Oschatz M. (2018). Small Methods.

[cit80] Geng Z., Liu Y., Kong X., Li P., Li K., Liu Z., Du J., Shu M., Si R., Zeng J. (2018). Adv. Mater..

[cit81] Andersen S. Z., Čolić V., Yang S., Schwalbe J. A., Nielander A. C., McEnaney J. M., Enemark-Rasmussen K., Baker J. G., Singh A. R., Rohr B. A., Statt M. J., Blair S. J., Mezzavilla S., Kibsgaard J., Vesborg P. C. K., Cargnello M., Bent S. F., Jaramillo T. F., Stephens I. E. L., Nørskov J. K., Chorkendorff I. (2019). Nature.

[cit82] Licht S., Cui B., Wang B., Li F. F., Lau J., Liu S. (2014). Science.

[cit83] Karfa P., Madhuri R., Sharma P. K., Tiwari A. (2017). Nano Energy.

[cit84] Chen W., Yang X., Chen Z., Ou Z., Hu J., Xu Y., Li Y., Ren X., Ye S., Qiu J., Liu J., Zhang Q. (2023). Adv. Funct. Mater..

[cit85] Zhao J., Chen Z. (2017). J.
Am. Chem. Soc..

[cit86] Shi R., Zhao Y., Waterhouse G. I. N., Zhang S., Zhang T. (2019). ACS Catal..

[cit87] Fujishima A., Honda K. (1972). Nature.

[cit88] Mehta P., Barboun P., Herrera F. A., Kim J., Rumbach P., Go D. B., Hicks J. C., Schneider W. F. (2018). Nat. Catal..

[cit89] Bogaerts A., Tu X., Whitehead J. C., Centi G., Lefferts L., Guaitella O., Azzolina-Jury F., Kim H. H., Murphy A. B., Schneider W. F., Nozaki T., Hicks J. C., Rousseau A., Thevenet F., Khacef A., Carreon M. (2020). J. Phys. D: Appl. Phys..

[cit90] Notter D., Elias Abi-Ramia Silva T., Gálvez M. E., Bulfin B., Steinfeld A. (2024). Mater. Horiz..

[cit91] Smith C., Hill A. K., Torrente-Murciano L. (2020). Energy Environ. Sci..

[cit92] Mayer P., Ramirez A., Pezzella G., Winter B., Sarathy S. M., Gascon J., Bardow A. (2023). iScience.

[cit93] Li Y., Lan S., Ryberg M., Pérez-Ramírez J., Wang X. (2021). iScience.

[cit94] D'Angelo S. C., Mache J., Guillén-Gosálbez G. (2023). ACS Sustainable Chem. Eng..

[cit95] D'Angelo S. C., Cobo S., Tulus V., Nabera A., Martín A. J., Pérez-Ramírez J., Guillén-Gosálbez G. (2021). ACS Sustainable Chem. Eng..

[cit96] D'Angelo S. C., Martín A. J., Cobo S., Ordóñez D. F., Guillén-Gosálbez G., Pérez-Ramírez J. (2023). Energy Environ. Sci..

[cit97] Mohamed A. M. O., Economou I. G., Bicer Y. (2024). Curr. Opin. Green Sustainable Chem..

[cit98] Mitchell S., Martín A. J., Pérez-Ramírez J. (2024). Nat. Chem. Eng..

[cit99] Ding S., Hülsey M. J., Pérez-Ramírez J., Yan N. (2019). Joule.

[cit100] Martín A. J., Shinagawa T., Pérez-Ramírez J. (2019). Chem.

[cit101] Ghavam S., Vahdati M., Wilson I. A. G., Styring P. (2021). Front. Energy Res..

[cit102] Ahmed H. S., Yahya Z., Khan W. A., Faraz A. (2024). Clean Energy.

[cit103] Mingolla S., Rosa L. (2025). Nat. Food.

[cit104] Ojelade O. A., Zaman S. F., Ni B.-J. (2023). J. Environ. Manage..

[cit105] Baltrusaitis J. (2017). ACS Sustainable Chem. Eng..

[cit106] Wang M., Khan M. A., Mohsin I., Wicks J., Ip A. H., Sumon K. Z., Dinh C.-T., Sargent E. H., Gates I. D., Kibria M. G. (2021). Energy Environ. Sci..

[cit107] Istrate R., Nabera A., Pérez-Ramírez J., Guillén-Gosálbez G. (2024). One Earth.

[cit108] Kyriakou V., Garagounis I., Vasileiou E., Vourros A., Stoukides M. (2017). Catal. Today.

[cit109] Sabatier P. and Senderens J.-B., Nouvelles synthèses du méthane, Académie des Sciences, Paris, 1902, vol. 134, p. 514

[cit110] Klier K., Chatikavanij V., Herman R. G., Simmons G. W. (1982). J. Catal..

[cit111] Struis R. P. W. J., Stucki S. (2001). Appl. Catal., A.

[cit112] Brunetti A., Pomilla F. R., Marcì G., Garcia-Lopez E. I., Fontananova E., Palmisano L., Barbieri G. (2019). Appl. Catal., B.

[cit113] Li T., Shoinkhorova T., Gascon J., Ruiz-Martinez J. (2021). ACS Catal..

[cit114] Kong S., Lv X., Wang X., Liu Z., Li Z., Jia B., Sun D., Yang C., Liu L., Guan A., Wang J., Zheng G., Huang F. (2022). Nat. Catal..

[cit115] Sun Z., Ma T., Tao H., Fan Q., Han B. (2017). Chem.

[cit116] Kuhl K. P., Hatsukade T., Cave E. R., Abram D. N., Kibsgaard J., Jaramillo T. F. (2014). J. Am. Chem. Soc..

[cit117] Huo Y., Zhang J., Dai K., Li Q., Lv J., Zhu G., Liang C. (2019). Appl. Catal., B.

[cit118] Wang Y., Liu X., Han X., Godin R., Chen J., Zhou W., Jiang C., Thompson J. F., Mustafa K. B., Shevlin S. A., Durrant J. R., Guo Z., Tang J. (2020). Nat. Commun..

[cit119] Yan T., Wang L., Liang Y., Makaremi M., Wood T. E., Dai Y., Huang B., Ali F. M., Dong Y., Ozin G. A. (2019). Nat. Commun..

[cit120] Ganji P., Chowdari R. K., Likozar B. (2023). Energy Fuels.

[cit121] Martin O., Pérez-Ramírez J. (2013). Catal. Sci. Technol..

[cit122] Ash-Kurlander U., Martin O., Fontana L. D., Patil V. R., Bernegger M., Mondelli C., Pérez-Ramírez J., Steinfeld A. (2016). Energy Technol..

[cit123] Frei M. S., Mondelli C., García-Muelas R., Kley K. S., Puértolas B., López N., Safonova O. V., Stewart J. A., Curulla Ferré D., Pérez-Ramírez J. (2019). Nat. Commun..

[cit124] Araújo T. P., Shah A., Mondelli C., Stewart J. A., Curulla Ferré D., Pérez-Ramírez J. (2021). Appl. Catal., B.

[cit125] Tsoukalou A., Bushkov N. S., Docherty S. R., Mance D., Serykh A. I., Abdala P. M., Copéret C., Fedorov A., Müller C. R. (2022). J. Phys. Chem. C.

[cit126] Zou T., Pinheiro Araújo T., Agrachev M., Jin X., Krumeich F., Jeschke G., Mitchell S., Pérez-Ramírez J. (2024). J. Catal..

[cit127] Pinheiro Araújo T., Giannakakis G., Morales-Vidal J., Agrachev M., Ruiz-Bernal Z., Preikschas P., Zou T., Krumeich F., Willi P. O., Stark W. J., Grass R. N., Jeschke G., Mitchell S., López N., Pérez-Ramírez J. (2024). Nat. Commun..

[cit128] Sushkevich V. L., Van Bokhoven J. A. (2019). ACS Catal..

[cit129] Ravi M., Ranocchiari M., van Bokhoven J. A. (2017). Angew. Chem., Int. Ed..

[cit130] Newton M. A., Knorpp A. J., Pinar A. B., Sushkevich V. L., Palagin D., Van Bokhoven J. A. (2018). J. Am. Chem. Soc..

[cit131] Sushkevich V. L., Palagin D., Ranocchiari M., Van Bokhoven J. A. (2017). Science.

[cit132] Sushkevich V. L., Palagin D., van Bokhoven J. A. (2018). Angew. Chem., Int. Ed..

[cit133] Cui Z., Meng S., Yi Y., Jafarzadeh A., Li S., Neyts E. C., Hao Y., Li L., Zhang X., Wang X., Bogaerts A. (2022). ACS Catal..

[cit134] Mei D., Duan G., Fu J., Liu S., Zhou R., Zhou R., Fang Z., Cullen P. J., (Ken) Ostrikov K. (2021). J. CO2 Util..

[cit135] Khadzhiev S. N., Kolesnichenko N. V., Ezhova N. N. (2016). Pet. Chem..

[cit136] Kotowicz J., Węcel D., Kwilinski A., Brzęczek M. (2022). Appl. Energy.

[cit137] Gao L., Jin H., Liu Z., Zheng D. (2004). Energy.

[cit138] Samaroo N., Koylass N., Guo M., Ward K. (2020). Green Chem..

[cit139] González-Garay A., Frei M. S., Al-Qahtani A., Mondelli C., Guillén-Gosálbez G., Pérez-Ramírez J. (2019). Energy Environ. Sci..

[cit140] Kondratenko E. V., Mul G., Baltrusaitis J., Larrazábal G. O., Pérez-Ramírez J. (2013). Energy Environ. Sci..

[cit141] Abanades J. C., Rubin E. S., Mazzotti M., Herzog H.
J. (2017). Energy Environ. Sci..

[cit142] Nabera A., Istrate I. R., Martín A. J., Pérez-Ramírez J., Guillén-Gosálbez G. (2023). Green Chem..

[cit143] Saad D. M., Terlouw T., Sacchi R., Bauer C. (2024). Environ. Sci. Technol..

[cit144] Gabrielli P., Goericke H., Rosa L. (2024). Ind. Eng. Chem. Res..

[cit145] Iribarren D., Calvo-Serrano R., Martín-Gamboa M., Galán-Martín Á., Guillén-Gosálbez G. (2022). Sci. Total Environ..

[cit146] Al-Qahtani A., González-Garay A., Bernardi A., Galán-Martín Á., Pozo C., Mac Dowell N., Chachuat B., Guillén-Gosálbez G. (2020). Appl. Energy.

[cit147] Methanol Institute, Renewable Methanol, https://www.methanol.org/renewable/, (accessed 29 March 2025)

[cit148] Fernández-González J., Rumayor M., Laso J., Domínguez-Ramos A., Irabien A. (2024). Sustainable Energy Fuels.

[cit149] Agyekum E. B., Okonkwo P. C., Rashid F. L. (2025). Carbon Res..

[cit150] Jiao J., Ma Y., Han X., Ergu A., Zhang C., Chen P., Liu W., Luo Q., Shi Z., Xu H., Chen C., Li Y., Lu T. (2025). Nat. Commun..

[cit151] Elwalily A., Verkama E., Mantei F., Kaliyeva A., Pounder A., Sauer J., Nestler F. (2025). Sustainable Energy Fuels.

[cit152] Martín A. J., Mondelli C., Jaydev S. D., Pérez-Ramírez J. (2021). Chem.

[cit153] Keller G. E., Bhasin M. M. (1982). J. Catal..

[cit154] Liu J., Yue J., Lv M., Wang F., Cui Y., Zhang Z., Xu G. (2022). Carbon Resour. Convers..

[cit155] Gerceker D., Motagamwala A. H., Rivera-Dones K. R., Miller J. B., Huber G. W., Mavrikakis M., Dumesic J. A. (2017). ACS Catal..

[cit156] Huang K., Miller J. B., Huber G. W., Dumesic J. A., Maravelias C. T. (2018). Joule.

[cit157] Zhou Y., Martín A. J., Dattila F., Xi S., López N., Pérez-Ramírez J., Yeo B. S. (2022). Nat. Catal..

[cit158] Das S., Pérez-Ramírez J., Gong J., Dewangan N., Hidajat K., Gates B. C., Kawi S. (2020). Chem. Soc. Rev..

[cit159] Veenstra F. L. P., Cibaka T., Martín A. J., Weigand D., Kirchhoff J., Smirnov V., Merdzhanova T., Pérez-Ramírez J. (2024). ChemSusChem.

[cit160] Larrazábal G. O., Martín A. J., Krumeich F., Hauert R., Pérez-Ramírez J. (2017). ChemSusChem.

[cit161] Hori Y., Kikuchi K., Suzuki S. (1985). Chem. Lett..

[cit162] Sánchez O. G., Birdja Y. Y., Bulut M., Vaes J., Breugelmans T., Pant D. (2019). Curr. Opin. Green Sustainable Chem..

[cit163] Rodríguez-Vallejo D. F., Guillén-Gosálbez G., Chachuat B. (2020). ACS Sustainable Chem. Eng..

[cit164] Ronda-Lloret M., Rothenberg G., Shiju N. R. (2019). ChemSusChem.

[cit165] Pawelec B., Guil-López R., Mota N., Fierro J., Navarro Yerga R. (2021). Materials.

[cit166] Li Z. (2025). Appl. Comput. Eng..

[cit167] Salah C., Istrate R., Bjørn A., Tulus V., Pérez-Ramírez J., Guillén-Gosálbez G. (2024). ACS Sustainable Chem. Eng..

[cit168] Tang S., Nozaki K. (2022). Acc. Chem. Res..

[cit169] Han D., Sun L., Chen L., Yang S., Li T., Xie X., Xu M., Tang W., Zhao B., Si H., Hua D. (2024). J. Fuel Chem. Technol..

[cit170] Ke L., Wu Q., Zhou N., Xiong J., Yang Q., Zhang L., Wang Y., Dai L., Zou R., Liu Y., Ruan R., Wang Y. (2022). Renewable Sustainable Energy Rev..

[cit171] Ma Z., Custodis V., Van Bokhoven J. A. (2014). Catal. Sci. Technol..

[cit172] Jampa S., Puente-Urbina A., Ma Z., Wongkasemjit S., Luterbacher J. S., Van Bokhoven J. A. (2019). ACS Sustainable Chem. Eng..

[cit173] Kabir G., Hameed B. H. (2017). Renewable Sustainable Energy Rev..

[cit174] Vispute T. P., Zhang H., Sanna A., Xiao R., Huber G. W. (2010). Science.

[cit175] Cheng Y. T., Jae J., Shi J., Fan W., Huber G. W. (2012). Angew. Chem., Int. Ed..

[cit176] Kleeb A. C., Edalat M. H., Gamper M., Haugstetter J., Giger L., Neuenschwander M., Kast P., Hilvert D. (2007). Proc. Natl. Acad. Sci. U. S. A..

[cit177] Koma D., Yamanaka H., Moriyoshi K., Ohmoto T., Sakai K. (2012). Appl. Environ. Microbiol..

[cit178] Bongaerts J., Krämer M., Müller U., Raeven L., Wubbolts M. (2001). Metab. Eng..

[cit179] Wu F., Cao P., Song G., Chen W., Wang Q. (2018). J. Chem. Technol. Biotechnol..

[cit180] Zhang J., Sun J., Wang Y. (2020). Green Chem..

[cit181] Sun J., Karim A. M., Zhang H., Kovarik L., Li X. S., Hensley A. J., McEwen J. S., Wang Y. (2013). J. Catal..

[cit182] Ji N., Ri P., Diao X., Rong Y., Kim C. (2023). Catal. Sci. Technol..

[cit183] Yan J., Li Z., Zhang Y., Liu R., Zhou L., Fu P. (2023). Fuel Process. Technol..

[cit184] Kim S., Kwon E. E., Kim Y. T., Jung S., Kim H. J., Huber G. W., Lee J. (2019). Green Chem..

[cit185] Singh O., Joshi B., Tiwari R., Goyal R., Luque R., Samanta C., Sarkar B. (2025). Chem. Eng. J..

[cit186] Nayak R. R., Gupta N. K. (2025). Green Chem..

[cit187] Han D., Sun L., Chen L., Yang S., Li T., Xie X., Xu M., Tang W., Zhao B., Si H., Hua D. (2024). J. Fuel Chem. Technol..

[cit188] Li J., Guan D., Xia S., Fan Y., Zhao K., Zhao Z., Zheng A. (2024). Energy Convers. Manage..

[cit189] Zoller F. A., Zimmerling E., Boutellier R. (2014). Higher Educ..

[cit190] Azoulay P. and Greenblatt W. H., Does Peer Review Penalize Scientific Risk Taking? Evidence from NIH Grant Renewals, MIT and Boston Children's Hospital/Harvard Medical School, Cambridge, MA and Boston, MA, 2025

[cit191] Carson R. T. , Graff Zivin J. S. and Shrader J. G., Choose Your Moments: Peer Review and Scientific Risk Taking, 2023, vol. 31409

[cit192] Li J., Sun Q., Yang F., Wang C., Feng K., Xin Y., Wang W., Li D. (2025). Commun. Earth Environ..

[cit193] Hu Z., Yan L., Yuan J., Etienne X. (2025). Food Policy.

[cit194] E. I. du Pont de Nemours and Company , US2130523A, 1938

[cit195] del Río González P. (2008). Ecol. Econ..

[cit196] Schmidt T. S. (2021). iScience.

[cit197] Millinger M., Hedenus F., Zeyen E., Neumann F., Reichenberg L., Berndes G. (2025). Nat. Energy.

[cit198] Huber G. W., Corma A., Corma A., Huber G. W. (2007). Angew. Chem., Int. Ed..

[cit199] Torrente-Murciano L., Smith C. (2023). Nat. Synth..

[cit200] Sietsma A. J., Theokritoff E., Biesbroek R., Canosa I. V., Thomas A., Callaghan M., Minx J. C., Ford J. D. (2024). One Earth.

[cit201] Callaghan M., Banisch L., Doebbeling-Hildebrandt N., Edmondson D., Flachsland C., Lamb W. F., Levi S., Müller-Hansen F., Posada E., Vasudevan S., Minx J. C. (2025). npj Clim. Action.

[cit202] Lück S., Callaghan M., Borchers M., Cowie A., Fuss S., Gidden M., Hartmann J., Kammann C., Keller D. P., Kraxner F., Lamb W. F., Mac Dowell N., Müller-Hansen F., Nemet G. F., Probst B. S., Renforth P., Repke T., Rickels W., Schulte I., Smith P., Smith S. M., Thrän D., Troxler T. G., Sick V., van der Spek M., Minx J. C. (2025). Nat. Commun..

[cit203] Peiseler L., Jun Y. L., Schmid N., Waidelich P., Malhotra A., Schmidt T. S. (2024). Environ. Res. Lett..

