# Supplementary material for: A global analysis of the rise, reign, and retreat of topics in research toward sustainable platform chemicals
Source: Green Chem. 2025 Oct 2;27(41):12985–3003. doi: 10.1039/d5gc02863a (PMC12490200; doi:10.1039/d5gc02863a)
Supplement: GC-027-D5GC02863A-s001 [file GC-027-D5GC02863A-s001.pdf]

Supplementary Information (SI) for Green Chemistry.
This journal is © The Royal Society of Chemistry 2025

Electronic Supplementary Information

A global analysis of the rise, reign, and retreat of topics in research toward sustainable platform chemicals

Paul Tautorat[a,b,d], Benedetta Tremolada[a], Antonio J. Martín[c,d], Lucas F. Santos[c,d], Gonzalo Guillén-Gosálbez[c,d], Javier Pérez-Ramírez[c,d], Bjarne Steffen*[a,d]

[a] *Climate Finance and Policy Group, Department of Humanities, Social and Political Sciences, ETH Zürich, Clausiusstrasse 37, 8092 Zurich, Switzerland.*
[b] *Energy and Technology Policy Group, Department of Humanities, Social and Political Sciences, ETH Zürich, Clausiusstrasse 37, 8092 Zurich, Switzerland.*
[c] *Institute for Chemical and Bioengineering, Department of Chemistry and Applied Biosciences, ETH Zürich, Vladimir Prelog Weg 1, Zürich 8093, Switzerland.*
[d] *NCCR Catalysis, Switzerland.*
**E-mail: bjarne.steffen@gess.ethz.ch*

Table of contents

1 Abbreviations and terminologies 2
2 Description of methodology 3
2.1 Stage 1: Data collection 3
2.2 Stage 2: Topic modeling 4
2.3 Stage 3: Topic analysis 5
3 Details on data collection 7
3.1 Query assembly 7
3.2 Final queries 9
3.3 Completeness test 9
3.4 Relevance test 12
4 Details on topic modeling 14
4.1 Pre-processing 14
4.2 Topic modeling 15
4.3 Hyperparameters tuning 15
5 Details on topic analysis 16
5.1 Initial topic analysis through generative AI 16
5.2 Topic refinement through human expertise 16
5.3 Post-processing through human expertise 20
6 Supplementary information on categories 25
7 Supplementary information on topics 27

1 Abbreviations and terminologies

Table S1 Abbreviations and terminologies used in the ESI.

Term	Abbreviation	Description
Ammonia	AM	Platform chemical analysed in this study.
Application Programming Interface	API	Used by Scopus to access bibliographic data, enabling efficient and automated downloading of document information.
Aromatics	AR	Platform chemical analysed in this study.
Bag of Words	BoW	An NLP technique to convert text into a structured numerical format by counting word frequency within documents, while ignoring grammar and word order.
Bigrams	–	Pairs of consecutive word tokens used in natural language processing to capture common word combinations.
Carbon Capture and Storage	CCS	A technology for capturing and storing CO_2, not included in our scope, as it belongs to the broader energy system.
Coherence	–	A metric used in topic modelling to evaluate the interpretability of a topic, based on the semantic consistency of its words.
Direct Air Capture	DAC	A method of capturing CO_2 directly from the atmosphere, excluded from scope for reasons similar to CCS.
Dominant Topic	DT	The topic with the highest probability assigned to a document during topic modelling, representing the topic that best captures the document's content.
Dominant Topic Contribution	DTC	A metric representing the proportion of a document associated with its dominant topic; used to filter documents strongly aligned with a single topic.
Hyperparameters	–	Parameters, as K, α, and β, that influence the performance of LDA and can be set and tuned to optimize the model's results.
–	K	Number of topics specified in the LDA model.
–	α (alpha)	Dirichlet prior parameter for the document-topic distribution in LDA.
–	β (beta)	Dirichlet prior parameter for the topic-word distribution in LDA.
–	max_df	Maximum document frequency threshold used to remove very common terms from the BoW representation (word tokens) during pre-processing.
–	min_df	Minimum document frequency threshold used to remove very rare terms from the BoW representation during pre-processing.
Latent Dirichlet Allocation	LDA	A generative probabilistic topic modelling algorithm used to uncover latent themes across the document corpus.
Methanol	ME	Platform chemical analysed in this study.
Natural Language Processing	NLP	A field of computer science focused on the interaction between computers and human language.
Olefins	OL	Platform chemical analysed in this study.
Perplexity	–	A metric used to evaluate how well a topic model predicts word distributions across documents; lower values indicate better model fit to the training set.
Platform Chemical	–	Chemicals like ammonia, aromatics, methanol, and olefins that serve as starting materials for producing a wide variety of downstream products.
Query	–	Search strings crafted for Scopus AI to retrieve relevant document corpora on specific chemicals or technologies.
Random Seed	–	Value set to ensure reproducibility of results in stochastic processes such as topic modelling.
Tokens	–	The total set of unigrams and bigrams extracted from the corpus during text preprocessing.
Topic	–	In NLP, a group of words frequently co-occurring in documents; in this study, also refers to the set of documents associated with the same dominant topic.
Unigrams	–	Single words treated as individual units in text analysis.

2 Description of methodology

We analysed research trends using a machine learning technique, specifically applying a topic model to a collection of academic literature as done before by other researchers.[1–4] To organize the analysis for the four platform chemicals and enhance reproducibility, we designed a three-stage workflow: Stage 1 involves collecting data for the four platform chemicals (depicted by the blue section in **Fig. S1**); Stage 2 applies the topic model to identify latent topics within the literature corpus (depicted by the green section in **Fig. S1**); and Stage 3 examines the distribution of the identified topics to assess research trends (depicted in grey in **Fig. S1**). While the workflow generally follows a sequential order, each stage can be iteratively revisited, allowing for dynamic, cyclical refinement. This structure enables better control, with expert judgment guiding each iteration to improve the dataset, model, and topic interpretation. Although Tautorat *et al.*[1] originally developed the workflow, we introduce a novel improvement by integrating probabilistic topic modelling, generative AI (genAI), and expert insights.

Table S2 The three stages of the developed workflow.

Stage	Input	Approach	Output
Stage 1 (blue)	Scopus database	Developing, tuning, and testing the search query	Datasets containing literature
Stage 2 (green)	Datasets containing literature	Processing raw data and tuning/training the topic model	Datasets containing topic distributions
Stage 3 (grey)	Datasets containing topic distributions	Labelling, grouping, and describing the topics	Analysis of research trends

This semi-automated method enabled a comprehensive analysis of over 90,000 documents. However, due to the specialized nature of the subject matter, each platform chemical was analysed individually, resulting in four datasets with varying document counts: 19,930 for ammonia, 16,156 for methanol, 31,582 for olefins, and 24,352 for aromatics. Analysing the platform chemicals separately not only clarified the distinct research trends specific to each group but also reduced pre-processing and training time. This approach allowed for multiple iterations at each stage, refining the final results.

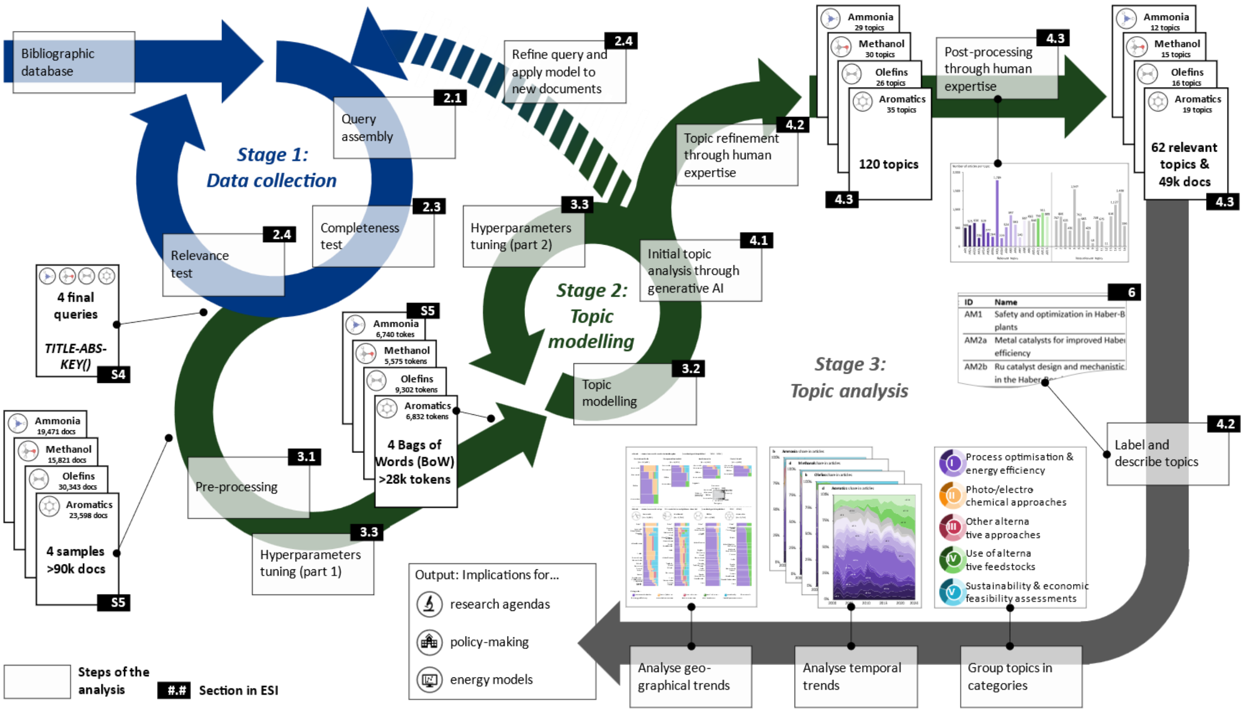

Fig. S1 Workflow followed for the analysis. The figure is an improvement of previous work by Tautorat *et al.*[1]

2.1 Stage 1: Data collection

First, we created one sample per platform chemical using the Scopus database by developing, tuning, and testing a search query (Stage 1). Scopus, a curated database by Elsevier,[5] was chosen as the reference bibliographic source for its reliability, comprehensive coverage, and relevance to chemical engineering research. It offers a good balance of accessibility and up-to-date scientific literature in the field. Scopus provides access to documents through a user-friendly application programming interface (API), allowing for efficient and automated downloading of bibliographic information for each document.

We found that the absence of full-text documents, which Scopus does not provide, did not significantly impact the analysis. Since the focus was on the overall context of the documents rather than specific technical details (*e.g.*, methodologies, experimental setups, or results), the abstracts alone were sufficient. From an operational standpoint, the text in the title, abstract, and keywords—provided by Scopus—was adequate to ensure that the topic model's performance was not compromised, given the large volume of literature analysed (from *ca.* 20,000 to 30,000 documents per dataset).[6] This also reduced text processing time. Alternative databases, such as Web of Science and Google Scholar, were not selected because they either lacked the breadth of coverage (as shown by Visser *et al.*[5]) or did not offer API-based access, limiting the reproducibility of query results.[7] OpenAlex, which includes working papers, was not used as chemical engineers rarely publish in this format.

For Stage 1, we implemented an API-based framework in Python to access the Scopus database directly, creating a targeted dataset through a structured search query. The query was designed to filter documents automatically based on specific research criteria. Its structure is explained in Section 3.1. The query was assembled to systematically expand its coverage, ensuring a comprehensive dataset while avoiding unrelated documents that could introduce noise into the analysis. Given that the quality and interpretability of the topic model results heavily depend on the dataset, we carefully refined the query through multiple rounds of testing at different stages of the process:

1. Tests at the sample level: The documents collected through the query were evaluated to assess the quality of the sample. To balance the sample's completeness and relevance, we developed two tests (details on the two tests are provided in Sections 3.3 and 3.4):
   - *Completeness test:* This test assessed how well different queries capture all relevant documents, examining whether the dataset includes all documents of interest for a well-founded analysis and interpretation.
   - *Relevance test:* This test evaluated the pertinence of the documents to the targeted subject, minimizing the inclusion of irrelevant documents.
2. Tests at the model results level: After analysing the topic modelling results, the query was refined to improve the quality and coherence of the topics. Specifically, the distribution of topics within the dataset was examined to:
   - *Exclude unrelated areas:* Given the broad scope of the terms related to platform chemical production in the query, some areas, while related to the broader field, were not pertinent to our analysis. We found it challenging to exclude these areas without compromising the dataset's completeness. The query was expanded to include these areas based on the topics obtained, assuming the model could distinguish unrelated documents into separate topics. The assumption was that as long as the model could effectively group irrelevant documents into distinct topics, the quality of the analysis would not be impacted (details on the exclusion are provided in Section 5.3).
   - *Include related areas*: The topic distribution was used to assess whether adding documents through query expansion would enhance the dataset's completeness without significantly affecting its relevance. By applying the pre-trained model to the expanded dataset, we evaluated how newly included documents were distributed across topics, particularly between relevant and non-relevant topics.

The tests, which mostly consisted of manually judging each document in the test samples, helped to understand which kind of documents the query was, or was not, able to include in the dataset. Based on this, we created the targeted datasets based on specific domain knowledge by manually assembling the query. At each iteration, experts carefully assessed and refined the structure of the query, including the addition or exclusion of terms. As a result, we did not establish a fixed threshold for these tests, as their primary goal was to evaluate the quality of the dataset in the Stage 1 loop. To prevent biased topics, we cleaned the dataset by both manually and automatically removing duplicates and irrelevant documents. Further details on the dataset post-processing are provided in Section 4.1.

2.2 Stage 2: Topic modelling

Second, we inferred document-level topic distributions across the dataset by developing, tuning, and applying a topic model (Stage 2). Topic modelling refers to a set of unsupervised machine learning methods used to uncover latent structures in text data, allowing for the systematic exploration of large bibliographic datasets. For this study, we employed Latent Dirichlet Allocation (LDA),[8,9] a widely used and well-established algorithm, with several implementations available in popular open-source libraries. Given a text-based representation of documents and model training as input, LDA generates topic-word and document-topic distributions as output. LDA performance was assessed using both intrinsic evaluation metrics and expert judgment. This stage was structured into three main phases: (i) Dataset pre-processing to create a suitable text representation; (ii) hyperparameter tuning and iterative topic refinement to improve results; (iii) dataset post-processing to further refine the analysis.

Dataset pre-processing

Prior to applying LDA, we pre-processed the dataset, ensuring that noise and inconsistencies did not negatively impact model performance. Further details on the dataset pre-processing are provided in Section 4.1. First, we manually and automatically removed duplicates and irrelevant documents to prevent biased topics. Second, we standardized the raw text within each document as follows. Titles, abstracts, and keywords were cleaned by removing irrelevant or excessively short words and phrases. A Bag of Words (BoW) model was then constructed to convert text into a structured numerical format suitable for topic modelling. In this step, words were

tokenized (*i.e.*, reduced to individual units of text) and counted based on their frequency within each document. Given the technical nature of the dataset, both individual words and word pairs were included, as specific multi-word terms often hold distinct meanings in chemical engineering and are more informative than single words, contributing to topic differentiation. Additionally, terms appearing either too frequently or too rarely in the BoW model were filtered out.

Topic modelling

We tuned LDA hyperparameters by evaluating model performance using intrinsic metrics. The final model was then trained on the entire dataset (details on model training are provided in Section 4.2, and hyperparameters tuning is described in Section 4.3).

First, we identified the hyperparameters that most significantly influenced model performance and defined appropriate value ranges for each. We then conducted a random search within these ranges to determine the final hyperparameters configuration. Among these, the number of topics—considered the most impactful parameter for topic interpretability—was treated differently. During the random search, this parameter remained variable: for each hyperparameters configuration tested, we trained multiple LDA models using different topic numbers, then averaged their performance to ensure robust tuning.

The number of topics was thus tuned separately in the subsequent step. This approach was necessary to ensure that the resulting topics would be semantically meaningful and suitable for identification as platform chemical production routes. Since LDA identifies topics as latent structures without considering their semantic meaning, careful evaluation of topic content was essential in selecting a reasonable value for this hyperparameter. While its initial range was defined alongside the other hyperparameters, its exact value was refined iteratively. First, an initial number of topics was chosen based on metrics scores. Then, it was adjusted through a three-step process: (i) automatic labelling and expert assessment of topic quality and relevance, (ii) refinement of the number of topics by increasing or decreasing it so that ill-defined topics would naturally be merged or split, guided by the previous expert evaluation; and (iii) re-running the LDA model with the updated number of topics, followed by re-evaluation of the results.

LDA produces probabilistic topic distributions rather than categorical classifications. To evaluate the topics, we assigned each document to the most representative topic, defined as its *dominant topic.* Specifically, the dominant topic of each document is the topic with the highest probability in that document. This allowed us to treat topics as groups of documents associated with the same dominant topic, allowing for both automated and expert-driven evaluations based on the content of the associated documents. Determining a reasonable number of topics required multiple iterations. To accelerate this process, we integrated generative AI into the workflow. Under expert supervision, generative AI was used to rapidly scan and summarize model outputs for configuration with different numbers of topics. This iterative process progressively improved the quality of the results, with each iteration reducing reliance on generative AI for topic assessment and increasing expert evaluation.

The process above ensured a systematic and rigorous approach to hyperparameters optimization. Once all hyperparameters were tuned, we trained the LDA model on the entire corpus and associated each document with its dominant topic. Details on the LDA setup, training procedures, and post-processing of results are presented in Section 4.2.

Dataset post-processing

At the final stage, documents within each topic were randomly evaluated to validate the topics' content. Critical documents incorrectly included in the initial sample were filtered out. Topics deemed non-relevant by experts were identified and excluded from the dataset. This process resulted in a dataset of 62 topics and 46,685 documents (12 topics with 9,316 documents for ammonia, 15 topics with 9,276 documents for methanol, 16 topics with 16,133 documents for olefins, and 19 topics with 11,960 documents for aromatics). Since the focus was on the direction of innovation in research, we further excluded documents published before the year 2000. After this refinement, the 62 final topics accounted for 39,103 documents, which were then analysed as research trends (12 topics with 8,102 documents for ammonia, 15 topics with 7,662 documents for methanol, 16 topics with 13,865 documents for olefins, and 19 topics with 9,474 documents for aromatics).

2.3 Stage 3: Topic analysis

Third, we categorised the identified topics and analysed them for temporal and geographical trends (Stage 3). To structure this analysis, we adapted the decarbonization framework proposed by Tautorat *et al.*[1] to reflect the specificities of the chemical industry (see **Fig. S2**). This framework is not intended as a central outcome of our study, but rather as a conceptual tool to guide the categorization process. It also provides context for the exclusion of research on Carbon Capture and Storage (CCS), Direct Air Capture (DAC), and hydrogen (H_2) production, which we classify as part of the broader energy system that supplies inputs to platform chemical production. Given the granularity of the topics and the aim to identify high-level trends, we derived five categories to group similar topics. The categories are based on the adapted framework and applicable to all four platform chemicals, allowing comparisons across the four platform chemicals: (I) Process optimisation and energy efficiency (primarily for thermochemical approaches); (II) Photo-/electrochemical approaches (as opposed to thermochemical); (III) Other alternative approaches; (IV) Use of alternative feedstocks; and (V) Sustainability and economic feasibility assessments.

We then analysed temporal trends in research activity from 2000 onward, focusing on the number of topics assigned to each category. Additionally, we examined the overall growth in research per platform chemical (Fig. 2, main text), as well sas compound annual

growth rates (CAGR) before and after the Paris Agreement—specifically for the periods 2000–2015 and 2016–2023 (Figs. 3 and 4, main text). To explore geographical patterns, each document was assigned to the country of the first author's affiliation. We compared the distribution of research across categories in four major world regions—East Asia and Pacific, Europe and Central Asia, North America, and the Rest of the World—following the World Bank's regional classification (Fig. 5, main text). Finally, for each platform chemical, we examined national-level contributions by analysing research outputs from the 20 most-publishing countries (2000–2024), ranked by the proportion of studies associated with category I ("Process optimisation and energy efficiency").

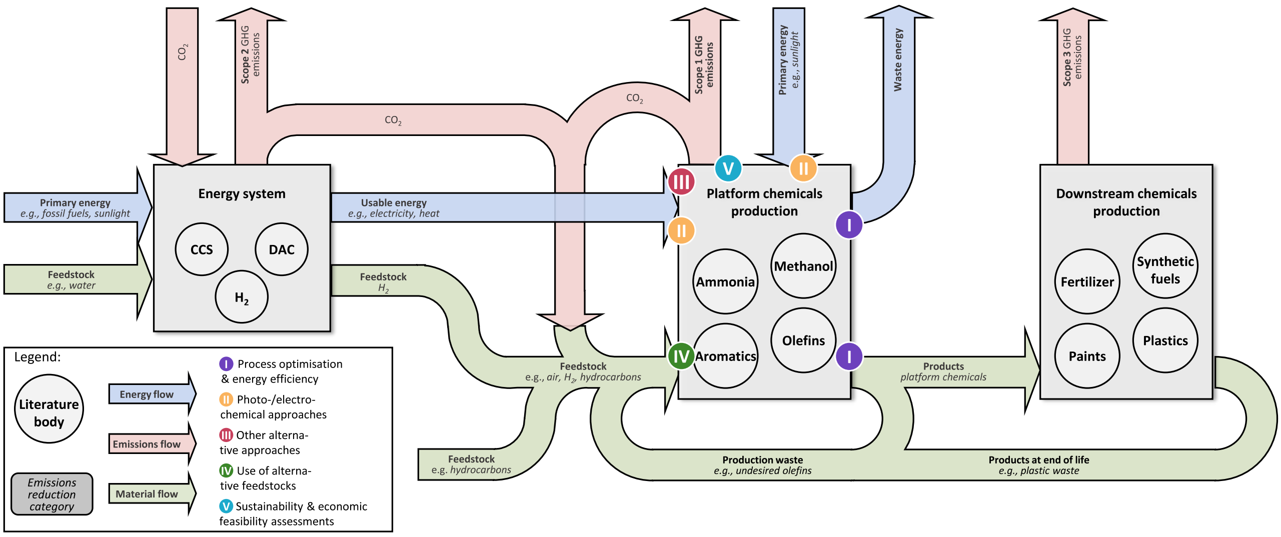

Fig. S2 Contextualization of platform chemicals production.

3 Details on data collection

3.1 Query assembly

The Elsevier APIs were accessed using the Python package *pybliometrics.*[10] This package facilitates the selection and retrieval of documents from Scopus based on a structured research string, referred to as a query. The query incorporates various elements and operators to filter documents according to specific research criteria. Selection parameters included language (**LANGUAGE**), publication year (**PUBYEAR**), document type (**DOCTYPE**), subject area (**SUBJAREA**), and content, *i.e.*, a combination of multiple words found in a document's title, abstract, or Scopus keywords (**TITLE-ABS-KEY**). We applied the same query structure for all four chemicals:

PUBYEAR AND LANGUAGE AND DOCTYPE AND SUBJAREA AND TITLE-ABS-KEY ((CHEMICAL TERMS W/X PRODUCTION TERMS) AND NOT EXCLUSION TERMS), where:

- The **AND** operator ensures both criteria are met,
- the **OR** operator allows one of the criteria to be met,
- the **AND NOT** operator excludes a specific criterion, and
- the **W/X** operator ensures terms are found within a specified proximity. For details on the syntax of Scopus queries, please refer to **Table S4**.

LANGUAGE: We excluded non-English documents. This decision was made to balance broader document coverage with dataset consistency, as applying topic modelling to multilingual datasets presents significant challenges from an NLP perspective. While this choice may impact the analysis of the geographical distribution of research—potentially underrepresenting some countries—we found that including non-English research would increase the number of documents by less than 10% for each dataset. This discrepancy is particularly pronounced in earlier decades, which were excluded from the analysis.

DOCTYPE: Among the diverse document types available in Scopus, we selected those most common in the chemical field, which together represent more than 95% of all document types for each query.

TITLE-ABS-KEY: In building the query, we carefully selected **CHEMICAL TERMS** to capture the majority of relevant studies on each chemical. While ammonia and methanol are more clearly defined, olefins and aromatics are often treated as collective groups of chemical compounds. For olefins, we focused primarily on ethylene and propylene, and for aromatics, on BTX (benzene, toluene, and xylene), given their significance as primary platform chemicals. Isolating individual substances in the literature proved challenging due to the inherent complexity of chemical processes and the diversity of compounds involved. Therefore, the queries reflect our chosen prioritization. Given the broad scope of these platform chemicals, we deliberately excluded certain precursors and processes related to one or more of the studied chemical compounds when they fell outside the specific focus on chemical production. For example, hydrogen was not explicitly included in the ammonia-related query, as we assumed the query would capture studies on H_2 together with to other areas like the Haber-Bosch process. Similarly, research on carbon capture was not directly included in the methanol query.

Although the primary objective was to explore decarbonization pathways, we designed the queries with broader coverage, meaning additional filters based on keywords like *sustainability*, *net zero emissions*, and *energy* were not explicitly included, resulting in larger samples. An evaluation of the results showed that many relevant documents addressing decarbonization pathways do not directly mention these terms (*e.g.*, fundamental laboratory research that does not explicitly discuss sustainability implications). The resulting datasets, which are 3-11 times larger, enhance the comprehensiveness of the analysis and improve topic model performance, as larger datasets optimize model training, even when only abstracts are used.[6]

Table S3 Details on the structure of the queries. Note: Search terms repeated across the four groups are greyed out for methanol, olefins, and aromatics to improve readability.

Selection parameter	Rationale	Ammonia	Methanol	Olefins	Aromatics
PUBYEAR	To capture up-to-date research, we included all documents available in Scopus up to the date of download.	< 2025	< 2025	< 2025	< 2025
LANGUAGE	Since English dominates the chemical engineering field, using English-language documents made both the analysis and topic modelling more feasible. While the share of non-English research has likely declined over the study period, we do not expect this to significantly impact the analysis of research direction, although it may slightly overestimate the increase in research intensity.	English	English	English	English
DOCTYPE	Articles (ar), conference papers (cp), and reviews (re) constitute the majority of relevant literature in Scopus. The "articles" category in Scopus also includes *Trade Journals*, which were manually removed later, as they contain industry news rather than research.	ar OR<br>cp OR<br>re	ar OR<br>cp OR<br>re	ar OR<br>cp OR<br>re	ar OR<br>cp OR<br>re
SUBJAREA	Among the not mutually exclusive subject areas provided by Scopus, we focused on those most relevant to the field (ceng, chem, ener, engi, mate, mult). For ammonia, the first platform chemical processed, additional areas were included (envi, soci, econ, phys, comp). After a detailed analysis of the added value, however, we did not include them for methanol, olefins, and aromatics.	ceng OR<br>chem OR<br>ener OR<br>engi OR<br>mate OR<br>mult OR<br>envi OR<br>soci OR<br>econ OR<br>phys OR<br>comp	ceng OR<br>chem OR<br>ener OR<br>engi OR<br>mate OR<br>mult	ceng OR<br>chem OR<br>ener OR<br>engi OR<br>mate OR<br>mult	ceng OR<br>chem OR<br>ener OR<br>engi OR<br>mate OR<br>mult
TITLE-ABS-KEY	To capture documents containing specific word combinations in the title, abstract, and Scopus keywords, we included documents where the CHEMICAL and PRODUCTION term appeared within three words (W/3), striking a balance between relevance and noise.	(*CHEMICAL TERMS* W/3 *PRODUCTION TERMS*) AND NOT *EXCLUSION TERMS*	(*CHEMICAL TERMS* W/3 *PRODUCTION TERMS*) AND NOT *EXCLUSION TERMS*	(*CHEMICAL TERMS* W/3 *PRODUCTION TERMS*) AND NOT *EXCLUSION TERMS*	(*CHEMICAL TERMS* W/3 *PRODUCTION TERMS*) AND NOT *EXCLUSION TERMS*
CHEMICAL TERMS	To identify documents related to the chemical of interest, we selected terms directly associated with the chemicals or processes predominantly linked to them. For ammonia, we intentionally did not include the term "hydrogen" to avoid an overwhelming focus on hydrogen research, which could shift attention away from ammonia. This should be considered when interpreting the results for ammonia, as sustainable hydrogen production is, after all, crucial for ammonia production.	ammonia OR<br>NH3 OR<br>"haber bosch"	methanol OR<br>CH3OH OR<br>MeOH OR<br>"methyl alcohol"	olefin* OR<br>alkene* OR<br>ethylene OR<br>ethene OR<br>C2H4 OR<br>propylene OR<br>propene OR<br>C3H6 OR<br>"steam cracking" OR<br>"Fischer Tropsch"	aromatic* OR<br>BTX OR arene* OR<br>benzene OR<br>benzol OR<br>cyclohexatriene OR<br>C6H6 OR<br>toluene OR<br>methylbenzene OR<br>toluol OR<br>phenylmethane OR<br>C7H8 OR<br>xylene* OR<br>dimethylbenzene OR<br>xylol OR<br>C8H10 OR<br>ethylbenzene
PRODUCTION TERMS	The research must focus on the industrial production of these chemicals to align with the aim of this study.	process OR<br>synthes* OR<br>produc* OR<br>industr* OR<br>manufact*	process OR<br>synthes* OR<br>produc* OR<br>industr* OR<br>manufact*	process OR<br>synthes* OR<br>produc* OR<br>industr* OR<br>manufact* OR<br>"alcohol dehydration" OR<br>"ethanol dehydration" OR<br>"propanol dehydration" OR<br>"methanol to olefin*"	process OR<br>synthes* OR<br>produc* OR<br>industr* OR<br>manufact* OR<br>distill* OR<br>separat* OR<br>"steam cracking" OR<br>"catalytic reform*"
EXCLUSIONS TERMS	The **AND NOT** operator was used to filter out documents that, although meeting the query's requirements, focused on unrelated topics. Terms to exclude were carefully selected to ensure a clear definition without compromising the sample's overall completeness.	"waste water" OR<br>wastewater OR<br>sewage OR<br>"treatment plant" OR<br>"water treatment" OR<br>"air conditioning" OR<br>chiller*	gasoline OR<br>biodiesel OR<br>diesel OR<br>methanolysis OR<br><br>(("dimethyl ether" OR<br>"ether" OR<br>"carbon tetrachloride" OR<br>DME OR<br>"dimethyl carbonate" OR<br>olefin* OR<br>hydrocarbon* OR<br>"formic acid" OR<br>formaldehyde* OR<br>formalin OR<br>"pichia pastoris" OR<br>lignin OR<br>acid OR<br>solvent)<br><br>W/1<br><br>(synthes* OR<br>reform* OR<br>produc* OR<br>conver* OR<br>extract*))	aviation OR<br>cycli?ation OR<br>diesel OR<br>engine OR<br>epoxidat* OR<br>gasoline OR<br>hydroformylat* OR<br>kerosene OR<br>metathesis OR<br>polymeri* OR<br>ship* OR<br>"transport fuel" OR<br>truck* OR<br><br>((carbonate OR<br>glycol OR<br>esther* OR<br>ether* OR<br>ketone* OR<br>aldehyde* OR<br>aceta* OR<br>poly* OR<br>aromatic* OR<br>oxide* OR<br>chloride OR<br>amine OR<br>vinyl* OR<br>DME OR<br>silane* OR<br>wax*)<br><br>W/3<br><br>(synthes* OR<br>reform* OR<br>produc* OR<br>conversion OR<br>extract*))	engine OR<br>"waste water" OR<br>wastewater OR<br>sewage OR<br>"treatment plant" OR<br>"water treatment" OR<br>poly OR<br><br>(plastic<br><br>W/3<br><br>(synthes OR<br>produc* OR<br>material*))

3.2 Final queries

Table S4 Final queries for the four platform chemicals.

Chemical	Full query	Date of download (yyyy-mm-dd)	Documents downloaded
Ammonia	TITLE-ABS-KEY((((ammonia OR nh3 OR "haber bosch") W/3 (process OR synthes* OR produc* OR industr* OR manufact*)) AND NOT ("waste water" OR wastewater OR sewage OR "treatment plant" OR "water treatment" OR "air conditioning" OR chiller*)) AND (a* OR a* OR a*)) AND SUBJAREA (ceng OR chem OR ener OR engi OR mate OR mult OR envi OR soci OR econ OR phys OR comp) AND DOCTYPE (ar OR cp OR re) AND LANGUAGE (english) AND PUBYEAR < 2025	2024-10-12	19,930
Methanol	TITLE-ABS-KEY((((methanol OR CH3OH OR MeOH OR "methyl alcohol") W/3 (process OR synthes* OR produc* OR industr* OR manufact*)) AND NOT (gasoline OR biodiesel OR diesel OR methanolysis OR ("dimethyl ether" OR "ether" OR "carbon tetrachloride" OR DME OR "dimethyl carbonate" OR olefin* OR hydrocarbon* OR "formic acid" OR formaldehyde* OR formalin OR "pichia pastoris" OR lignin OR acid OR solvent) W/1 (synthes* OR reform* OR produc* OR conver* OR extract*))) AND (a* OR a* OR a*)) AND SUBJAREA (ceng OR chem OR ener OR engi OR mate OR mult OR envi) AND DOCTYPE (ar OR cp OR re) AND LANGUAGE (english) AND PUBYEAR < 2025	2024-09-08	16,156
Olefins	TITLE-ABS-KEY((((olefin* OR alkene* OR ethylene OR ethene OR C2H4 OR propylene OR propene OR C3H6 OR "steam cracking" OR "Fischer Tropsch") W/3 (process OR synthes* OR produc* OR industr* OR manufact* OR "alcohol dehydration" OR "ethanol dehydration" OR "propanol dehydration" OR "methanol to olefin*")) AND NOT (epoxidat* OR hydroformylat* OR polymeri* OR cycli?ation OR metathesis OR gasoline OR diesel OR "transport fuel" OR kerosene OR aviation OR ship* OR truck* OR engine OR (carbonate OR glycol OR esther* OR ether* OR ketone* OR aldehyde* OR aceta* OR poly* OR aromatic* OR oxide* OR chloride OR amine OR vinyl* OR DME OR silane* OR wax*) W/3 (synthes* OR reform* OR produc* OR conversion OR extract*))) AND (a* OR a* OR a*)) AND SUBJAREA (ceng OR chem OR ener OR engi OR mate OR mult OR envi) AND DOCTYPE (ar OR cp OR re) AND LANGUAGE (english) AND PUBYEAR < 2025	2024-09-03	31,582
Aromatics	TITLE-ABS-KEY((((aromatic* OR BTX OR arene* OR benzene OR benzol OR cyclohexatriene OR C6H6 OR toluene OR methylbenzene OR toluol OR phenylmethane OR C7H8 OR xylene* OR dimethylbenzene OR xylol OR C8H10 OR ethylbenzene) W/3 (process OR synthes* OR produc* OR industr* OR manufact* OR distill* OR separat* OR "steam cracking" OR "catalytic reform*")) AND NOT (engine OR "waste water" OR wastewater OR sewage OR "treatment plant" OR "water treatment" OR poly OR (plastic W/3 (synthes OR produc* OR material*)))) AND (a* OR a* OR a*)) AND SUBJAREA (ceng OR chem OR ener OR engi OR mate OR mult OR envi) AND DOCTYPE (ar OR cp OR re) AND LANGUAGE (english) AND PUBYEAR < 2025	2024-09-05	24,352
Reference sample	PUBYEAR < 2025 AND DOCTYPE (ar OR cp OR re) AND LANGUAGE (english)	2025-02-10	54,403,194

Table S5 Stepwise filtering of the four datasets.

Selection parameter	Comment	Ammonia	Methanol	Olefins	Aromatics
Scopus total	This number varies for the four platform chemicals, as datasets were downloaded at slightly different points in time, and Scopus continuously updates and reindexes the database.	91,801,943	91,357,131	91,271,111	91,321,706
PUBYEAR		91,78,1243	91,349,719	91,264,750	91,314,871
LANGUAGE		81,265,531	80,868,912	80,789,191	80,836,107
DOCTYPE		73,148,019	72,814,313	72,745,248	72,788,683
SUBJAREA		41,311,357	28,398,166	28,365,514	28,387,180
TITLE-ABS-KEY	This includes CHEMICAL, PRODUCTION, and EXCLUSION TERMS (see **Table S3**).	19,930	16,156	31,582	24,352

The terms used in the **TITLE-ABS-KEY** operator define the content of the documents included in the dataset, thereby influencing the query's coverage of the field. During query tuning, these terms were carefully selected based on the following guiding question: *Does adding a new term expand the query's coverage and improve the sample's completeness, while maintaining or enhancing its relevance?*

3.3 Completeness test

To evaluate the performance of different queries in terms of dataset completeness, we assessed whether all relevant documents were included. The testing procedure involved: (i) compiling a list of publications by selecting relevant references from different, manually chosen review articles; (ii) verifying if these references were included in the dataset; (iii) analysing missing references to identify the reasons for exclusion, such as query filters; and (iv) refining the query to capture similar relevant content.

For the final completeness test, we selected four unseen review articles (previously not studied or used for testing) through a systematic procedure. We searched for review articles on the respective chemical in Scopus, filtering for those published after 2019 and ranking them by citation count, using the following query format:

TITLE-ABS (chemical AND (production OR synthesis)) OR AUTHKEY (chemical AND (production OR synthesis)) AND PUBYEAR > 2019 AND SUBJAREA (ceng OR chem OR ener) AND DOCTYPE (re) (see individual queries for each chemical below). We then screened the most cited results and selected the first review article that met these three criteria:

1. *Scope should not be too broad:* The review should focus on the production of the respective chemical (*e.g.*, reviews on the "ammonia" or "methanol economy" were excluded as they were too broad and cited too many irrelevant documents).

2. *Scope should not be too narrow:* The review should cover the entire production process of the chemical (*e.g.*, a document on ammonia should not focus exclusively on hydrogen as a precursor, and a review on olefins should not be limited to the Fischer-Tropsch process).
3. *Pathway scope should not be too narrow:* The review should address a wide range of pathways for the respective chemical (*e.g.*, documents focusing exclusively on ammonia electrification or catalyst roles were considered too narrow).

The completeness scores for the four datasets are summarized in the tables below (see **Tables S6**-**S9**). Among the references not originally included in the sample (referred to as 'missing'), those that were inaccessible via Scopus ('Available in Scopus') or evaluated as irrelevant to the analysis were excluded. The final score was calculated as the percentage of references included ('Included') relative to the total number of references that should have been included ('Included' + 'Should Have Been Included').

Selection of review articles

Table S6 Queries used to search for review articles in Scopus for the four platform chemicals. The search was conducted on March 3, 2025.

Chemical	Full query
Ammonia	TITLE-ABS (ammonia AND (production OR synthesis)) OR AUTHKEY (ammonia AND (production OR synthesis)) AND PUBYEAR > 2019 AND SUBJAREA (ceng OR chem OR ener) AND DOCTYPE (re).
Methanol	TITLE-ABS (methanol AND (production OR synthesis)) OR AUTHKEY (methanol AND (production OR synthesis)) AND PUBYEAR > 2019 AND SUBJAREA (ceng OR chem OR ener) AND DOCTYPE (re)
Olefins	TITLE-ABS (olefins AND (production OR synthesis)) OR AUTHKEY (olefins AND (production OR synthesis)) AND PUBYEAR > 2019 AND SUBJAREA (ceng OR chem OR ener) AND DOCTYPE (re)
Aromatics	TITLE-ABS ((aromatic OR xylene OR toluene OR benzene) AND (production OR synthesis)) OR AUTHKEY ((aromatic OR xylene OR toluene OR benzene) AND (production OR synthesis)) AND PUBYEAR > 2019 AND SUBJAREA (ceng OR chem OR ener) AND DOCTYPE (re)

Table S7 Ammonia: Choice of review article for completeness test.

No	Title	DOI	Citations	Reason not to use
1	A Roadmap to the Ammonia Economy	10.1016/j.joule.2020.04.004	1094	not specific enough
2	Recent Advances and Challenges of Electrocatalytic N2Reduction to Ammonia	10.1021/acs.chemrev.9b00659	898	too specific
3	Recent development of hydrogen and fuel cell technologies: A review	10.1016/j.egyr.2021.08.003	624	not ammonia-focused (hydrogen)
4	Review on ammonia as a potential fuel: From synthesis to economics	10.1021/acs.energyfuels.0c03685	621	too specific
5	A review of ammonia as a compression ignition engine fuel	10.1016/j.ijhydene.2019.12.209	598	too specific
6	Oxygen Evolution Reaction in Alkaline Environment: Material Challenges and Solutions	10.1002/adfm.202110036	401	too specific
7	The future of hydrogen: Challenges on production, storage and applications	10.1016/j.enconman.2022.116326	385	not ammonia-focused (hydrogen)
8	Strategies to suppress hydrogen evolution for highly selective electrocatalytic nitrogen reduction: Challenges and perspectives	10.1039/d0ee03596c	377	not ammonia-focused (hydrogen)
9	Recent advances in nanostructured heterogeneous catalysts for N-cycle electrocatalysis	10.26599/NRE.2022.9120010	366	too specific
10	Ammonia as an effective hydrogen carrier and a clean fuel for solid oxide fuel cells	10.1016/j.enconman.2020.113729	339	too specific
11	Development and Recent Progress on Ammonia Synthesis Catalysts for Haber–Bosch Process	10.1002/aesr.202000043	324	too specific
**12**	**Sustainable Ammonia Production Processes**	**10.3389/fenrg.2021.580808**	**322**	***Chosen for completeness test***

Table S8 Methanol: Choice of review article for completeness test.

No	Title	DOI	Citations	Reason not to use
1	State of the Art and Prospects in Metal-Organic Framework (MOF)-Based and MOF-Derived Nanocatalysis	10.1021/acs.chemrev.9b00223	1869	too specific
2	Recent Advances in Carbon Dioxide Hydrogenation to Methanol via Heterogeneous Catalysis	10.1021/acs.chemrev.9b00723	1062	too specific
3	State of the art and perspectives in heterogeneous catalysis of CO_2 hydrogenation to methanol	10.1039/c9cs00614a	784	too specific
4	Recent development of hydrogen and fuel cell technologies: A review	10.1016/j.egyr.2021.08.003	624	not methanol-focused
5	Core-shell structured catalysts for thermocatalytic, photocatalytic, and electrocatalytic conversion of CO_2	10.1039/c9cs00713j	598	too specific
**6**	**Power-to-liquid via synthesis of methanol, DME or Fischer–Tropsch-fuels: a review**	**10.1039/d0ee01187h**	**453**	***Chosen for completeness test***

Table S9 Olefins: Choice of review article for completeness test.

No	Title	DOI	Citations	Reason not to use
1	Electrocatalytic reduction of CO_2 and CO to multi-carbon compounds over Cu-based catalysts	10.1039/d1cs00535a	414	too specific
2	Recent progress in heterogeneous metal and metal oxide catalysts for direct dehydrogenation of ethane and propane	10.1039/d0cs01260b	265	too specific
3	Carbon-based catalysts for Fischer-Tropsch synthesis	10.1039/d0cs00905a	256	too specific
4	Oxide-Zeolite-Based Composite Catalyst Concept That Enables Syngas Chemistry beyond Fischer-Tropsch Synthesis	10.1021/acs.chemrev.0c01012	250	too specific
5	Towards the development of the emerging process of CO_2 heterogenous hydrogenation into high-value unsaturated heavy hydrocarbons	10.1039/d1cs00260k	236	too specific
6	Current status and perspectives in oxidative, non-oxidative and CO_2-mediated dehydrogenation of propane and isobutane over metal oxide catalysts	10.1039/d0cs01140a	228	too specific
7	Pyrolysis technology for plastic waste recycling: A state-of-the-art review	10.1016/j.pecs.2022.101021	226	too specific
8	Atropisomers beyond the C–C axial chirality: Advances in catalytic asymmetric synthesis	10.1016/j.chempr.2022.04.011	224	too specific
9	Nitrogen-Centered Radicals in Functionalization of sp2 Systems: Generation, Reactivity, and Applications in Synthesis	10.1021/acs.chemrev.1c00831	224	too specific
10	Waste tyre valorization by catalytic pyrolysis – A review	10.1016/j.rser.2020.109932	210	too specific
11	Recent advances in carbon dioxide hydrogenation to produce olefins and aromatics	10.1016/j.chempr.2021.02.024	193	too specific
12	Polyolefin thermoplastic elastomers from polymerization catalysis: Advantages, pitfalls and future challenges	10.1016/j.progpolym-sci.2020.101342	175	too specific
13	Propane to olefins tandem catalysis: A selective route towards light olefins production	10.1039/d1cs00357g	167	too specific
14	Recent advances in nickel mediated copolymerization of olefin with polar monomers	10.1016/j.ccr.2021.213802	158	too specific
15	Nickel Catalyzed Olefin Oligomerization and Dimerization	10.1021/acs.chemrev.0c00076	156	too specific
16	Catalytic Enantio- And Regioselective Addition of Nucleophiles in the Intermolecular Hydrofunctionalization of 1,3-Dienes	10.1021/acscatal.9b04712	151	too specific
17	Fatty Acids and their Derivatives as Renewable Platform Molecules for the Chemical Industry	10.1002/anie.202100778	151	too specific
18	Rational Design of Bioinspired Catalysts for Selective Oxidations	10.1021/acscatal.0c02073	150	too specific
19	Aromatics Production via Methanol-Mediated Transformation Routes	10.1021/acscatal.1c01422	142	Not olefins-specific
20	Recent advances in nickel-catalyzed reductive hydroalkylation and hydroarylation of electronically unbiased alkenes	10.1007/s11426-020-9838-x	136	too specific
21	Recent advances in transition metal-catalyzed olefinic C-H functionalization	10.1039/d0qo01159b	124	too specific
22	An overview of Fischer-Tropsch Synthesis: XtL processes, catalysts and reactors	10.1016/j.apcata.2020.117740	119	too specific
23	Transition-metal-catalyzed C–H allylation reactions	10.1016/j.chempr.2020.10.020	116	too specific
24	Halogen-mediated electrochemical organic synthesis	10.1039/d0ob01008a	112	too specific
25	Development of direct conversion of syngas to unsaturated hydrocarbons based on Fischer-Tropsch route	10.1016/j.chempr.2021.08.019	103	too specific
**26**	**Light olefin synthesis from a diversity of renewable and fossil feedstocks: state-of-the-art and outlook**	**10.1039/d1cs01036k**	**100**	***Chosen for completeness test***

Table S10 Aromatics: Choice of review article for completeness test. Note: For aromatics, sorting the results by number of citations did not yield sensible results. Instead, we sorted by relevance.

No	Title	DOI	Citations	Reason not to use
1	A review of advances in production and separation of xylene isomers	10.1016/j.cep.2021.108603	54	Too specific
**2**	**Production of gasolines and monocyclic aromatic hydrocarbons: From fossil raw materials to green processes**	**10.3390/en14134061**	**47**	***Chosen for completeness test***

Table S11 Results of the completeness test.

Chemical	DOI review article	No. Ref	Included	Missing	Available in Scopus	Should have been included	Score of final tests
Ammonia	10.3389/fenrg.2021.580808	102	27	75	27/ 75	0/ 27	100%
Ethanol	10.1039/d0ee01187h	435	71	364	153/364	19/153	92%
Olefins	10.1039/d1cs01036k	464	218	246	233/246	182/233	59%
Aromatics	10.3390/en14134061	242	16	226	136/226	22/136	85%

3.4 Relevance test

The relevance of each dataset was evaluated by: (i) compiling a random sample of documents from the dataset; (ii) assessing their pertinence to the study's aim; and (iii) refining the query to exclude, or avoid including, irrelevant documents. To ensure comprehensive coverage of the field of interest, the query was designed broadly, enhancing the sample's completeness and improving model performance. However, this broad scope led to some loss of focus, as irrelevant documents from unrelated areas were inevitably included. To mitigate this, the relevance test was designed to be dependent on the topic model results, assuming the model could separate irrelevant documents into distinct, non-relevant topics. As long as the model groups non-relevant documents into separate topics, the quality of the analysis remains unaffected, and these documents are excluded from the final dataset. The relevance test is thus conducted only on the relevant dataset.

For the evaluation process, a random 5% sample of documents from the relevant dataset was selected. The evaluation involved two steps. (i) The relevance of each document was automatically assessed using the GPT-4-0125-preview model with a binary 'yes' or 'no' label assigned, leveraging its semantic understanding to assess the relevance of each document in the tested samples. For this, we developed a prompt specifically for each chemical to account for the unique characteristics of each dataset (see **Table S12** for details on GPT prompts).

Table S12 GPT prompts for the relevance test in Stage 1. Note: The placeholder [chemical] was substituted with ammonia, methanol, olefins, or aromatics according to the specific chemical being analysed.

Chemicals	GPT prompt
Common to all chemicals	You are an expert in industrial [chemical] production and topic modelling. You are applying Latent Dirichlet Allocation (LDA) to analyse trends in academic research on [chemical] production. Using the Scopus API, you have retrieved a sample of papers related to industrial [chemical] production based on a search query. Your response must be "yes" or "no" (return yes or no all lowercase, without punctuation or additional text).
Ammonia	You are now provided with the abstract of one paper from the sample. Your task is to assess whether the paper is relevant to the study of ammonia production/synthesis (*e.g.*, papers related to ammonia synthesis/production via current/alternative/innovative/future/lab-scale/fundamental/chemical/engineering routes/technologies/methods etc.). Include also as relevant everything that is somehow related to ammonia production (*e.g.*, all the studies related to ammonia plants, or environmental/techno/economic feasibility of ammonia production.).<br><br>Examples of non-relevant papers include studies on the utilization or application of ammonia in industrial settings, research where ammonia is an intermediate rather than the targeted product, or research on non-industrial production methods such as biological or environmental processes.
Methanol	You are now provided with the abstract of one paper from the sample. Your task is to assess whether the paper is relevant to the study of methanol production/synthesis (*e.g.*, papers related to methanol synthesis/production via current/alternative/innovative/future/lab-scale/fundamental/chemical/engineering routes/technologies/methods etc.). Include also as relevant everything that is somehow related to methanol production (*e.g.*, all the studies related to methanol plants, separation of methanol when it is the desired target (*e.g.*, in distillation processes), or environmental/techno/economic feasibility of methanol production.).<br><br>Examples of non-relevant papers include studies on the utilization or application of methanol in industrial settings, research where methanol is an intermediate rather than the targeted product (*e.g.*, Methanol-to-Olefins, Methanol-to-Aromatics), or research on non-industrial production methods such as biological or environmental processes.
Olefins	You are now provided with the abstract of one paper from the sample. Your task is to assess whether the paper is relevant to the study of olefins production/synthesis (*e.g.*, papers related to olefins synthesis/production via current/alternative/innovative/future/lab-scale/fundamental/chemical/engineering routes/technologies/methods etc.). Include also as relevant everything that is somehow related to olefins production (*e.g.*, all the studies related to olefins plants, separation of olefins when they are the desired targeted product (*e.g.*, in distillation/separation processes), or environmental/techno/economic feasibility of olefins production.).<br><br>Papers on Fischer-Tropsch are all relevant. Examples of non-relevant papers include studies on the utilization or application of olefins in industrial settings, research where olefins are an intermediate rather than the targeted product, or research on non-industrial production methods such as biological or environmental processes.
Aromatics	You are now provided with the abstract of one paper from the sample. Your task is to assess whether the paper is relevant to the study of aromatics compounds production/synthesis (*e.g.*, papers related to aromatics synthesis or production via current/alternative/innovative/future/lab-scale/fundamental/chemical/engineering routes/technologies/methods etc.). Include also as relevant everything that is somehow related to aromatics production (*e.g.*, all the studies related to aromatics plants, separation of aromatics when they are the desired targeted product (*e.g.*, in distillation/separation processes), or environmental/techno/economic feasibility of aromatics production.).<br><br>Examples of non-relevant papers include studies on the utilization or application of aromatics in industrial or other settings, research where aromatics are an intermediate rather than the targeted product, or research on non-industrial production methods such as biological or environmental processes.

(ii) To verify if GPT's assessments accurately reflected the true distribution of relevance in the dataset, a random 10% subsample of the evaluated documents was manually reviewed to compare GPT's judgments with manual labels to confirm that GPT's relevance predictions were reliable.

Table S13 Relevance test scores for each chemical.

Relevance	Details	Ammonia	Methanol	Olefins	Aromatics
Sample for relevance	5% of the relevant dataset	466	464	807	598
Relevance score	By GPT	74%	68%	84%	61%
Agreement with GPT	Manual-GPT; based on 10% (of the 5%)	96%	96%	91%	92%

4 Details on topic modelling

4.1 Pre-processing

To clean and standardize the dataset for reliable topic modelling, we combined document-level filtering with text-level cleaning before creating the textual representations.

Dataset cleaning

We found that most of the noise in the dataset originated from duplicate documents and missing abstracts. To address this, we applied both automatic and manual filtering methods, as shown in **Table S14**. Since both conference papers and review documents were included in the query, redundancy was likely introduced. This duplication could artificially increase the weight of specific terms in the bag-of-words representation, potentially skewing topic distribution and hindering the identification of distinct themes.[11] Despite the cleaning process, some duplicates—such as documents published in multiple journals or different versions of the same work—are expected to remain, contributing to the overrepresentation of certain topics. However, a substantial amount of repetition is needed for duplication to significantly impact model inference.[11] Additionally, documents without abstracts or containing only graphical abstracts were filtered out, as we found that titles alone did not provide enough information for the analysis, and their number was negligible relative to the total dataset.

Table S14 Details on the cleaning process.

		No. publications after removal of targeted documents			
**Cleaning**	**Removal of targeted documents**	**Ammonia**	**Methanol**	**Olefins**	**Aromatics**
Before cleaning		19,930	16,156	31,582	24,352
Automatic	same "title" and " author_ids"	19,857	16,079	31,137	24,265
	same "title" and "author_afids"	19,847	16,069	31,137	24,251
	same beginning of abstract [200 characters incl. spaces]	19,531	15,860	30,525	23,846
	same end of abstract [200 characters incl. spaces]	19,478	15,831	30,368	23,606
	same "title" and "creator"	19,473	15,824	30,353	23,600
Manual	same title but different abstract	19,472	15,822	30,351	23,599
Automatic	no abstract	19,471	15,821	30,343	23,598

Bag of Words

To process the raw text in the dataset, we used the Python package *SpaCy*[12] due to its optimized performance[13] and user-friendly structure, specifically the lightweight English language model (en_core_web_sm) for efficiency. Text normalization was performed in several steps: Cleaning: Irrelevant words and phrases were removed. We applied SpaCy's default stop word list but retained specific terms relevant to the analysis (*e.g.*, "well," "more," "increase," "decrease"). Dataset-specific elements such as HTML tags, copyright notices, empty values, numeric data, and publishing-specific expressions (*e.g.*, "my/our/this paper") were also removed. Short words (one or two characters) were eliminated to avoid uninformative tokens. Standardization: The text was standardized by converting all text to lowercase, replacing accented characters (*e.g.*, é to e), and applying lemmatization[14] to reduce words to their base forms (*e.g.*, "concentrating" to "concentrate"). Concatenation: A unique text for each document was created by combining the cleaned and standardized individual texts from the title, abstract, and keywords into a single string. Since the dataset included both specific lab research and broader review papers, key information could be found in any of the three sections, so we assumed equal weight for all. BoW Model Creation: Once the text was normalized, we created the Bag of Words (BoW)[15] model using *CountVectorizer* (CV) from the Python package *Scikit-learn.*[16] We tokenized the text, converting words into features and creating a document-term matrix where each row represented a document and each column a unique token. The matrix values represented token frequency within each document.

In the BoW model, not all tokens were useful, so we decided which to keep. CV allowed us to define the *ngram_range* parameter to specify the word combinations included in the model. We included both unigrams and bigrams (ngram_range = (1,2)), as technical vocabulary often includes meaningful multi-word terms like "nitrogen reduction" or "nitrogen interaction," which could lead to different topics. Including both unigrams and bigrams resulted in a vocabulary size of approximately 1 million tokens per platform chemical, leading to prolonged preprocessing times. The vocabulary included terms that appeared either too frequently (*e.g.*, "ammonia") or too rarely (*e.g.*, highly technical terms), which were not particularly useful for topic identification. To reduce model overfitting and computational complexity, we applied minimum (min_df) and maximum (max_df) document frequency thresholds to filter out rare or overly common words. Over 90% of the total tokens in the vocabulary were bigrams. Since bigrams were much less frequent than unigrams, applying the same threshold effectively eliminated many irrelevant bigrams, significantly reducing the vocabulary size. The specific threshold value was tuned alongside other model hyperparameters.

4.2 Topic modelling

The resulting document-term matrix was fed into an LDA model, which analysed word co-occurrence patterns to infer underlying topics. We used the LDA implementation from *Scikit-learn,*[16] which applies the online variational Bayes algorithm to approximate the posterior distributions of topics and words. We set the hyperparameters for full batch learning, with 100 iterations (sufficient for our model's convergence) and a fixed seed to ensure reproducibility. The prior parameters α and β, as well as the number of topics (K), were determined during hyperparameter tuning. Once set, the model was trained on the entire dataset, yielding: (i) A document-topic distribution matrix, where each row represents a document, each column represents a topic, and values indicate the probability of a document belonging to a given topic. (ii) A word-topic distribution, showing which words are most representative of each topic. To evaluate topics as clusters of documents rather than probability distributions, we assigned each document to its dominant topic (the topic contributing most to its generation) and defined the *dominant topic contribution* (DTC) as the extent to which the dominant topic contributes to that document. The dataset was thus divided into K mutually exclusive groups of documents, where K is the number of topics.

4.3 Hyperparameters tuning

The tuning process involved iteratively testing different hyperparameter configurations and evaluating model performance using intrinsic metrics. We used perplexity, a standard measure of predictive performance, as defined in the original LDA paper by Blei *et al.*.[8] Perplexity measures model fit by averaging word likelihoods across documents. Lower perplexity on a test set indicates better generalization and reflects how well the model predicts unseen data. However, Chang *et al.*[17] showed that lower perplexity does not always correspond to more interpretable topics. We observed that as the number of topics increases, perplexity initially decreases but then stabilizes, while topic quality, as judged by humans, stops improving at this point. To address this, we complemented perplexity with a topic coherence metric, as proposed by Mimno *et al.,*[18] which evaluates each topic based on the degree of co-occurrence among its most probable words across the corpus, with higher coherence scores reflecting more semantically interpretable topics.

Hyperparameters range selection

An initial range for hyperparameter values was identified based on the perplexity and coherence metrics. To do so, we evaluated each hyperparameter individually by running the model with different values while keeping others fixed. This allowed us to assess how variations in each hyperparameter impacted model performance and identify their appropriate value ranges. The hyperparameters found important to be tuned were: α and β (parameters of the Dirichlet distributions); min_df and max_df (document frequency thresholds); K (number of topics). We set a lower threshold for K at 10 based on perplexity, and an upper limit of 50 to ensure manageable results for manual evaluation. α and β control topic sparsity in documents and word sparsity in topics, respectively.[19] We found that different initializations had minimal impact when K was not large,[14] so the range for both parameters was centred around the default value of 1/K. Vocabulary size was also tuned. Lowering max_df slightly increased perplexity but did not impact coherence, as frequent words were not meaningful for topic differentiation. Lowering min_df increased perplexity greatly, so we chose this threshold to exclude rare words as min_df > 20.

Hyperparameters optimization

To systematically identify a good combination of α, β, min_df, and K, we performed a random search[20] within the previously selected ranges using the open-source library *tm_toolkit*[21] for parallel computation. Since K was the most critical parameter and required manual tuning, it was determined in the second step of a two-step approach. First, random combinations of values for α, β, and min_df were selected and evaluated based on the average performance of several models, each with the same combination of α, β, and min_df but different K (within its range of 10 to 50). The metrics' values for these models were averaged for each combination of α, β, and min_df, and stability was assessed via error propagation. This process was repeated for approximately 200 random α, β, and min_df combinations, with the final set chosen based on model performance and stability. Second, once the final values for α, β, and min_df were determined, K was manually tuned. 50 models were then run, each with the K values ranging from 1 to 50. Each of these 50 models was also run 50 times to ensure performance stability. The most suitable K was selected based on: (i) the highest average value and stability in performance metrics; (ii) expert evaluation using GPT-assisted topic naming, similarity matrices, and top-word analysis (see Section 5.2); (iii) Sankey diagrams to visualize topic merging and splitting across different K values (an example is shown in **Fig. S3**). Ultimately, as 50 models were run for each K values, we selected the one with the highest performance metrics for the final K using a fixed random seed.

Table S15 Choice of hyperparameters and resulting tokens for each platform chemical.

Chemical	min_df	max_df	α	β	K range	K final	Random Seed	Tokens in BoW	Unigrams in BoW	Bigrams in BoW
Ammonia	42	1.0	0.067	0.133	18-32	29	8,045	6,740	3,488	3,252
Methanol	36	1.0	0.028	0.022	25-34	30	5,994	5,575	3,267	2,595
Olefins	39	1.0	0.070	0.315	22-28	26	2,735	9,302	4,476	5,027
Aromatics	43	1.0	0.041	0.260	27-39	35	4,565	6,832	3,716	3,057

5 Details on topic analysis

5.1 Initial topic analysis through generative AI

The GPT-4-0125-preview model was incorporated into the analysis to perform various tasks. In Stage 2, as part of the loop to refine the number of topics, GPT was used to interpret the topic modelling results by analysing the content of each topic and generating descriptive labels to facilitate expert evaluation. For each topic, GPT assessed its relevance based on the abstracts of the five documents with the highest Dominant Topic Contribution (DTC), generating an appropriate title. These documents were selected because they were the most strongly associated with that topic, making them the most representative. After the loop, GPT was further utilized to generate concise descriptions of the final relevant topics, based on the abstracts of the 15 documents with the highest DTC.

Table S16 GPT prompts for topics analysis tasks. Note: the placeholder [chemical] was substituted with ammonia, methanol, olefins, or aromatics according to the specific chemical being analysed. The placeholder [topic X] was substituted with the number of the specific topic being analysed.

Task	GPT Prompt
Title	You are an expert in Latent Dirichlet Allocation (LDA) topic modelling and have applied LDA to a collection of academic publications about [chemical]. The results have identified several topics, with [topic X] being one of them. The text above contains five academic abstracts that are highly representative of [topic X]. While these abstracts exemplify the content of [topic X], there are hundreds of publications within this topic. Your task is to generate a title for [topic X]. This title should reflect the main theme of these abstracts, capturing their common elements while remaining broad and general enough to cover all publications within the [topic X]. It should be a concise and comprehensive summary of the recurring content without focusing on specific details. Your response must be just the title. Make it as concise as possible and avoid filler words/phrases like “advancements in” or “advances in”.
Topic relevance	You are an expert in Latent Dirichlet Allocation (LDA) topic modelling and have applied LDA to a collection of academic publications about [chemical]. The results have identified several topics, with [topic X] being one of them. The text above contains five academic abstracts that are highly representative of [topic X]. Based on these five abstracts, your task is to assess the relevance of [topic X]. Specifically, determine if this topic is relevant now or in the future for the industrial production of [chemical] as a final product. In other words, does [topic X] focus on research related to current or future production routes of [chemical] in industrial settings, rather than on research such as [chemical] production in other settings? The project is focused on the direction of innovation of research in platform chemicals ([chemical]). Your response must be: ‘yes’ or ‘no’ (with all lowercase letters).
Description	You are an expert in topic modelling and chemicals industrial production and have applied Latent Dirichlet Allocation (LDA) to a collection of academic publications about [chemical] production. The model has identified several topics, with [topic X] being one of them. You are provided with the abstracts of 15 articles that belong to this topic and are ranked highest by the LDA model for this topic. Based on these abstracts, your task is to describe [topic X] content. Specifically, you have to respond with ONE sentence no longer than 150 characters where you explain on what the topic focuses on/explore/dive in. The description should provide a high-level overview of the topic focus. Your description should have the structure of this example: [topic X] on "Safety and optimization in [chemical] plants". The description should be like: "Safety and efficiency in [chemical] production through risk management." Start directly with the description, not writing "[topic X] " and avoiding "This topic explores/focuses on/investigates/covers, etc." and "Advancements in/Enhancements in etc.", as in the example. Be concise, technical and avoid trivial adjectives as "efficient/innovative etc." or "for ammonia synthesis".

5.2 Topic refinement through human expertise

To ensure that each topic can be interpreted as a specific (characteristic of a) production process for platform chemicals, each iteration of the loop in Stage 2 was supervised by domain-specific experts from the author team. This is crucial because while LDA is effective at identifying hidden themes, it does not capture deeper semantic relationships. The experts' involvement increased as the topic model results improved. Initially, the manual evaluation process included the following steps: First, examining the most relevant words.[22] Second, evaluating topic titles and relevance: Based on the abstracts of the five documents with the highest Dominant Topic Contribution (DTC), generative AI assessed topic relevance and generated a title. As the results improved, additional evaluation steps were introduced. For each topic, the experts also manually assessed:

(i) *A set of random documents:* To select documents for evaluation, the DTC range from 0.3 to 0.8 was divided into 10 equal bins, each representing a range of DTC values. One document from each bin was randomly selected, ensuring an unbiased evaluation of internal cohesion by avoiding frequency-based bias. This approach provided a balanced overview of each topic’s content. It also allowed us to examine how the content of documents associated with the same topic, but with different topic contributions, varies. Specifically, we assessed whether documents with lower DTC still relate closely to the core theme of the topic or diverge significantly.

(ii) *Dominant topic contribution distributions:* Well-defined topics were expected to consist of documents with consistently high DTC values. A DTC distribution skewed toward lower values or a broad distribution indicated a less coherent topic.

(iii) *Similarity matrix:* Since each document was generated by one or more topics, we examined the overlap between topics to determine if multiple topics were contributing to the same cluster of documents. This helped identify cases where the model failed to distinguish between quality topics (see **Fig. S4**-**Fig. S7**). To construct the similarity matrix, for each dominant topic, we selected its group of documents. For each group of documents, we then calculated the average contribution of every other topic. The resulting matrix has topics on both axes, with each row representing a dominant topic and the columns showing the average contribution of the other topics across all documents associated with the dominant topic.

Based on these evaluations, topics were classified as well-defined or ill-defined. Topics were adjusted by merging or splitting as needed. Experts used Sankey diagrams, created with the Python package *Plotly,*[23] to visualize how documents were distributed across topics between runs (see AM9 in **Fig. S3**). This ensured that changes in the number of topics improved topic quality. For example, documents on plasma-assisted ammonia synthesis were initially grouped into an irrelevant topic. Increasing the number of topics allowed for better separation of relevant plasma documents from unrelated ones, resulting in a new plasma topic. This approach also

revealed that some topics remained stable regardless of the number of topics, highlighting the robustness of certain themes across the dataset. Tracking how topics split during runs provided valuable insights into their content.

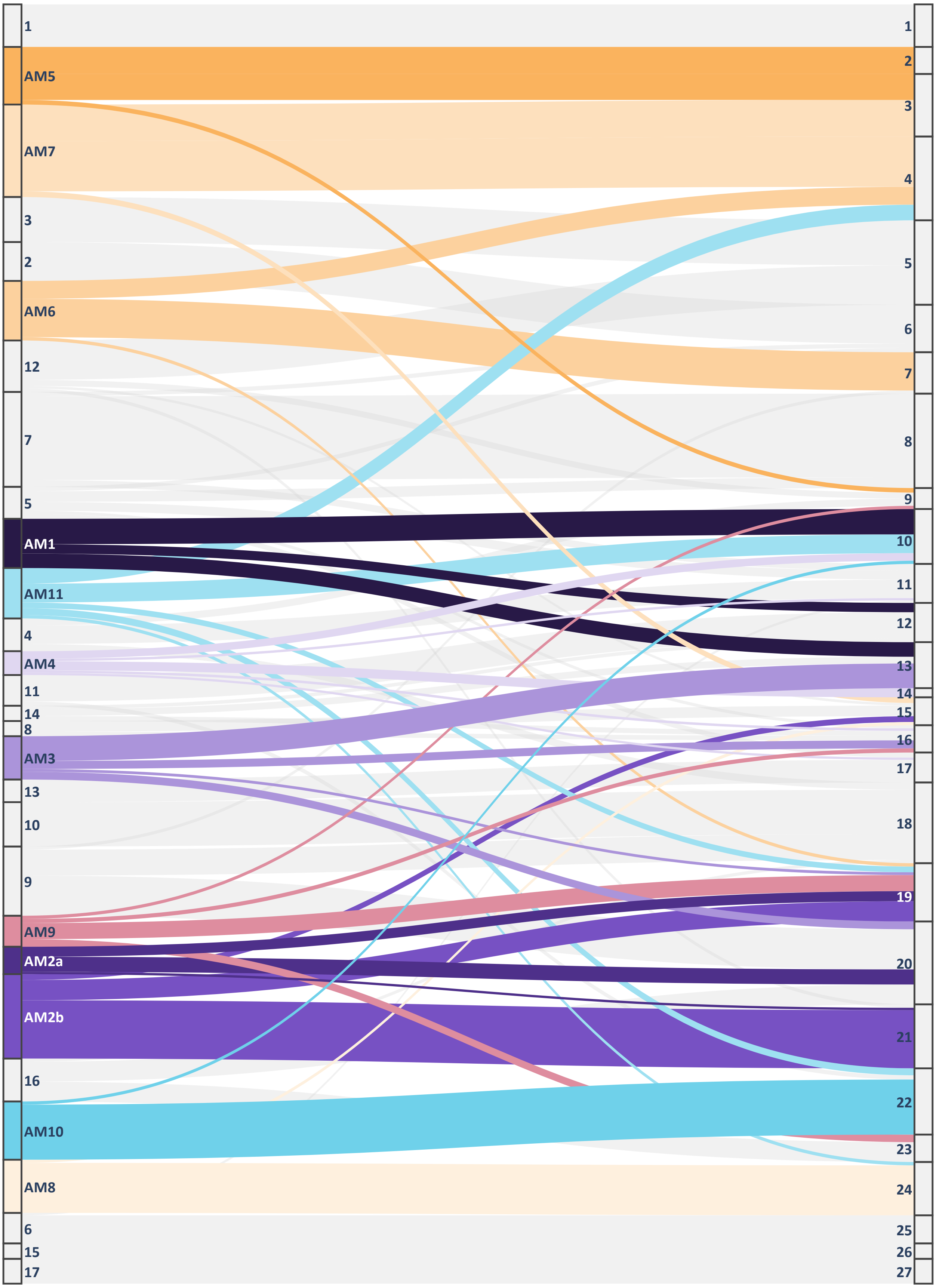

Fig. S3 Sankey diagram allowing for manual tuning of the number of topics. Exemplary diagram for ammonia of topics from the final run with 29 topics (left; AM1-AM11 and 17 non-relevant topics) linked to a previous run with 27 topics (right).

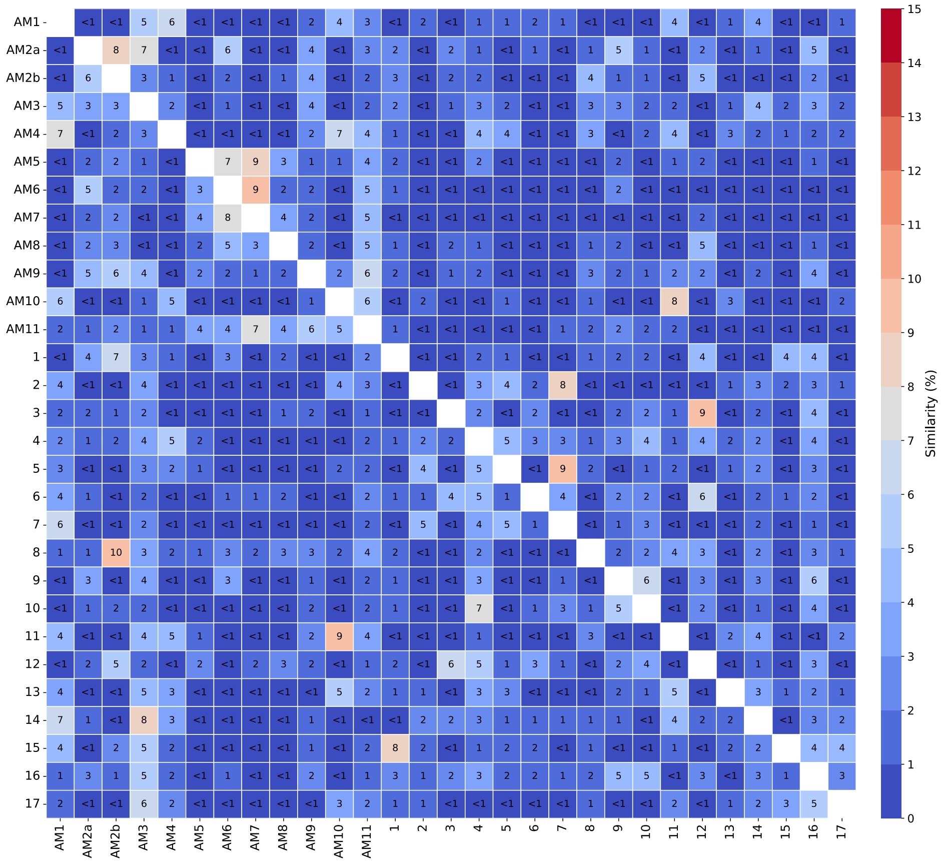

Fig. S4 Ammonia: Similarity matrix.

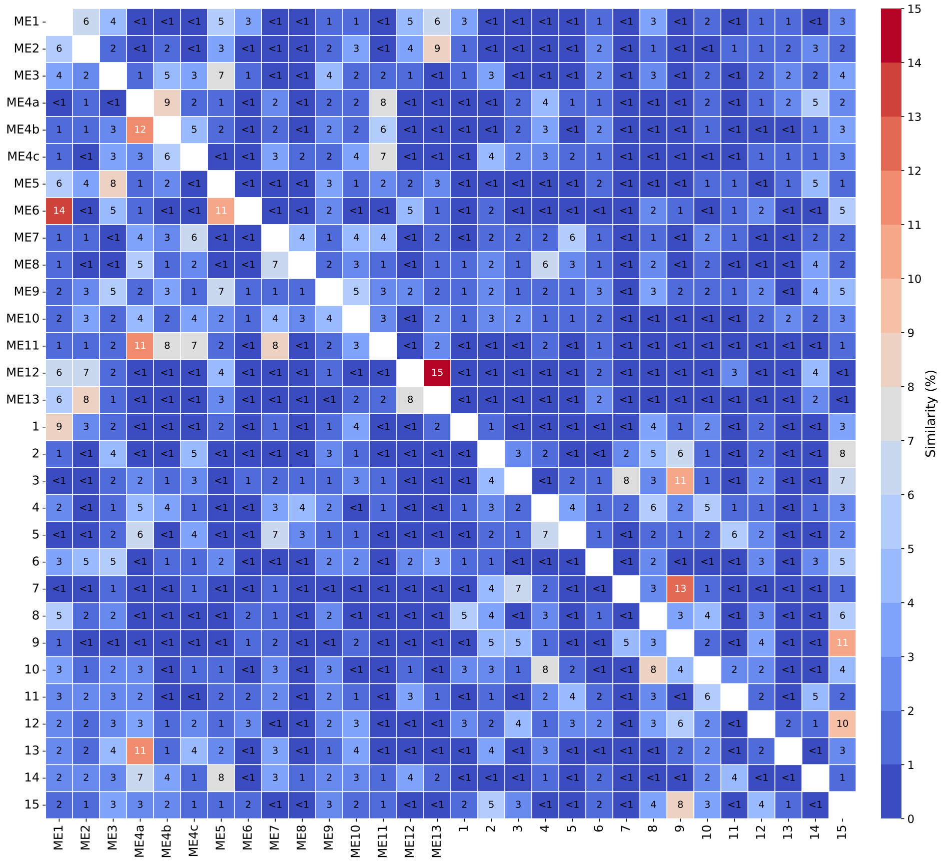

Fig. S5 Methanol: Similarity matrix.

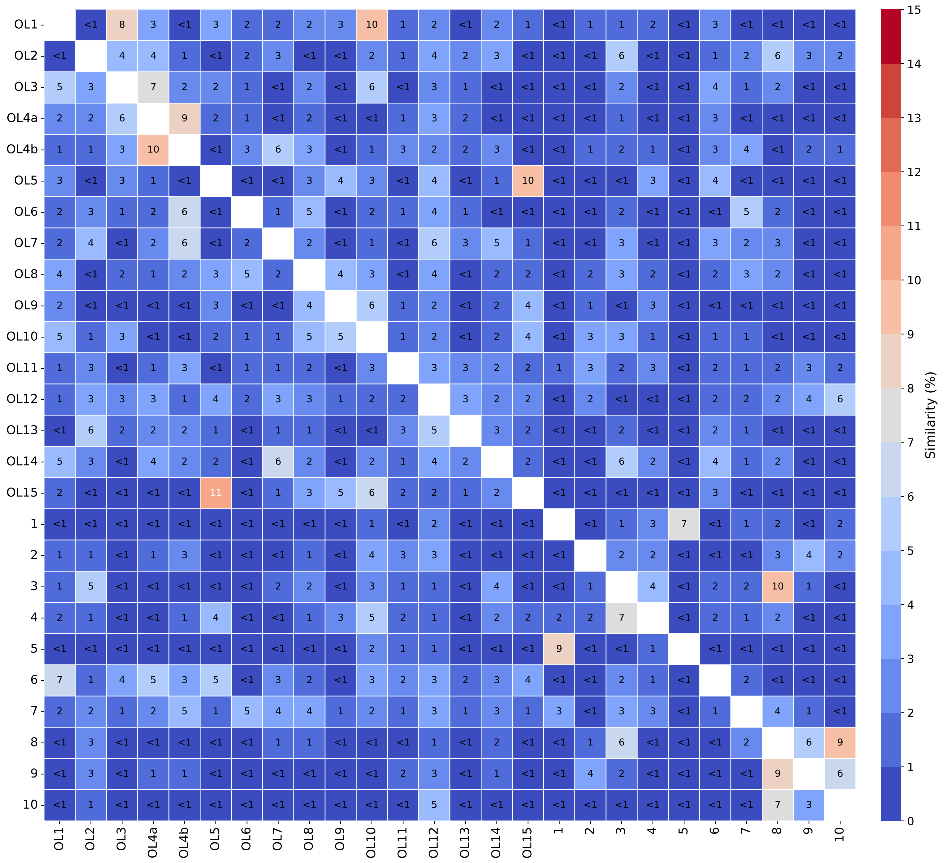

Fig. S6 Olefins: Similarity matrix.

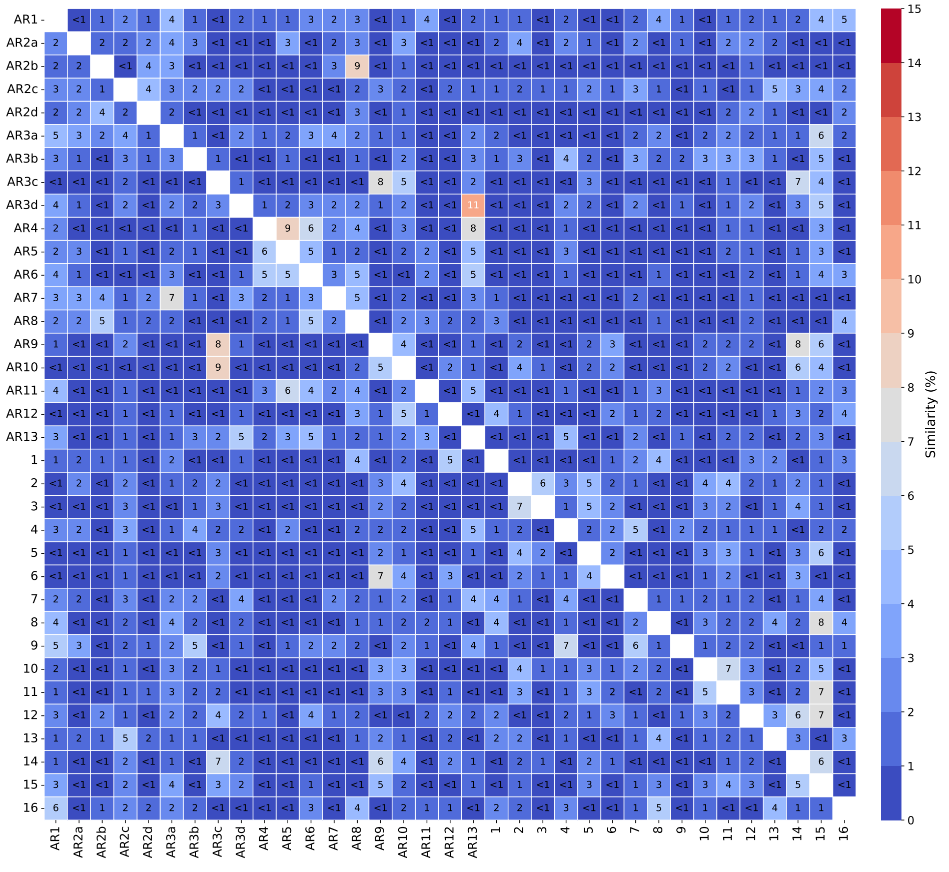

Fig. S7 Aromatics: Similarity matrix.

5.3 Post-processing through human expertise

We post-processed the final dataset by filtering out documents that were wrongly included and non-relevant topics. s, to address wrongly included documents: (i) During the examination of the final set of topics, we found that one topic for olefins, OL10, contained documents that did not align with our query, but were included because they were automatically tagged with "Fischer-Tropsch" as an index keyword by Scopus. To correct this, we re-downloaded the dataset using **TITLE-ABS OR AUTHKEY** instead of **TITLE-ABS-KEY** to avoid index keywords and filtered out documents missing from the new dataset. (ii) We also identified that some documents categorized as "Trade Journal" articles were labelled by Scopus as "articles". Since these were primarily irrelevant to our research, we removed them from each chemical dataset. (iii) Several documents in the olefins dataset focused on glycol ethylene and other unrelated compounds. These were filtered out using a post-exclusion terms approach. Second, regarding non-relevant topics: As mentioned in Stage 1, the search query was fine-tuned to exclude specific areas to maximize relevance (*e.g.*, secondary applications). However, due to the heterogeneity of terms across parallel sectors, a clear-cut separation was not always possible. For example, the term "catalyst" appears in both platform chemical synthesis documents and studies on unrelated compound reduction. To maintain completeness, we included these areas in the initial dataset and then used topic modelling to separate relevant topics from irrelevant ones. This approach preserved significant studies while enabling manual filtering of non-relevant topics. Topics not pertaining to any relevant areas were labelled as "non-relevant" and excluded from further analysis (see **Table S17** and **Fig. S8** for the example of Aromatics).

Table S17 Relevant and non-relevant topics for each chemical.

Chemical	Topics	Relevant topics	Dataset all topics	Dataset relevant topics	Dataset relevant topics from 2000 on
Ammonia	29	12	19,471	9,316	8,102
Methanol	30	15	15,821	9,276	7,662
Olefins	26	16	30,343	16,133	13,865
Aromatics	35	19	23,598	11,960	9,474

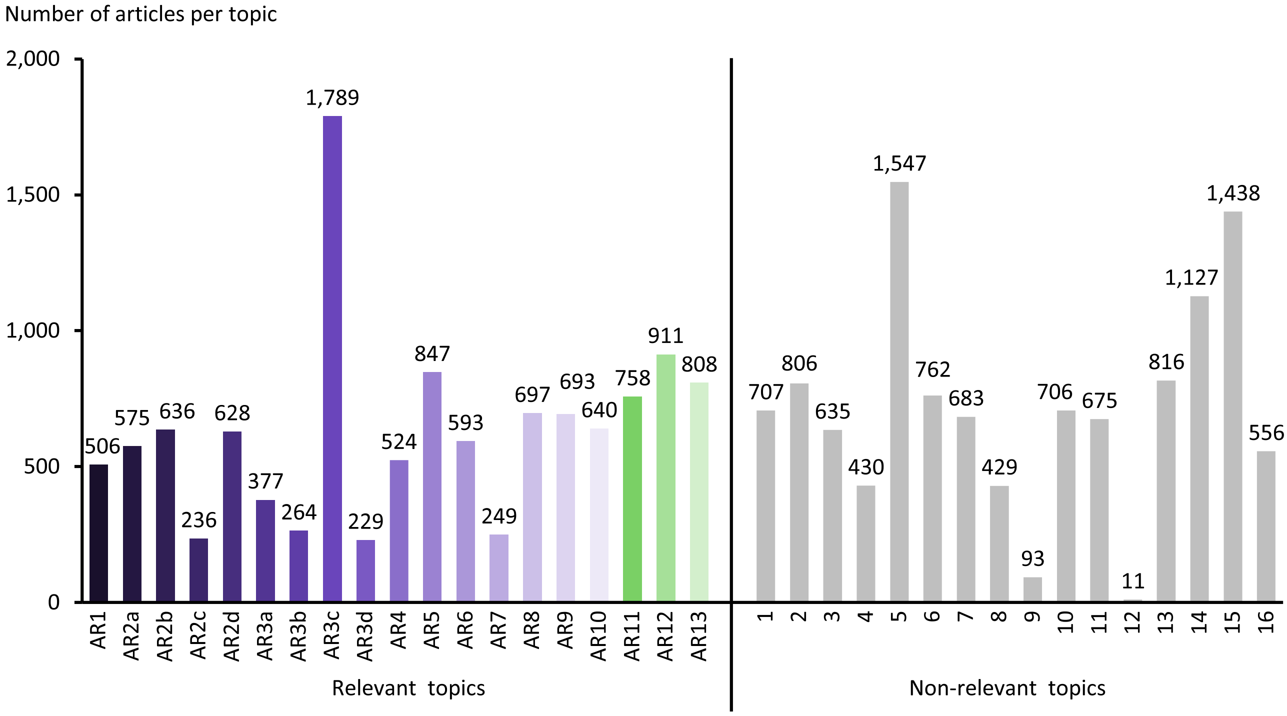

Fig. S8 Aromatics: Distribution of documents across relevant (AR1-AR13) and non-relevant topics (1-16).

Table S18 Ammonia: List of relevant and non-relevant topics and most relevant tokens per topic. Note: Topic names for the irrelevant topics (1-17) are genAI-based and not manually reviewed or refined.

ID	Name short	Most relevant tokens
AM1	HB: Safety & optimization	plant, plant growth, safety, control, ammonia plant, design, optimization, process, risk, growth promote
AM2a	HB: Metal catalysts	surface, adsorption, cluster, desorption, dissociation, activation, coverage, site, adsorb, 111
AM2b	HB: Ru-based catalysts	catalyst, support, iron, synthesis, ammonia synthesis, activity, ruthenium, catalyst ammonia, ammonia, promoter
AM3	HB: Process simulation & reactor design	model, kinetic, reactor, reaction, rate, experimental, simulation, equilibrium, datum, equation
AM4	Syngas production	gas, oil, gasification, steam, leach, methanol, natural, coal, natural gas, syngas
AM5	Catalysts for electrochemical synthesis from nitrates	nitrate, no3, nitrate reduction, reduction, nitrite, electrode, electrochemical, no3rr, cm, cathode
AM6	Computational studies of single-atom catalysts	atom, theory, functional, density functional, functional theory, single, density, calculation, single atom, dft
AM7	Catalysts for electrochemical synthesis from nitrogen	nrr, reduction, nitrogen reduction, electrocatalyst, reduction reaction, electrochemical, electrocatalytic, nitrogen, ambient, ambient condition
AM8	Catalysts for photochemical synthesis	photocatalytic, tio2, light, photocatalyst, fixation, vacancy, visible, visible light, nitrogen fixation, oxygen vacancy
AM9	Other approaches (*e.g.*, plasma-assisted)	plasma, pressure, synthesis, ammonia synthesis, discharge, ammonia, atmospheric pressure, atmospheric, temperature, high pressure
AM10	Environmental & economic feasibility	production, cost, energy, renewable, green, hydrogen, economic, ammonia, fuel, green ammonia
AM11	Reviews on alternative routes	review, haber, bosch, haber bosch, recent, bosch process, process, development, nitrogen, challenge
1	Enhancements in NH_3-SCR catalysts for NO_x reduction: mechanisms, poisoning resistance, and catalytic	oxidation, scr, nh3, zeolite, nh3 scr, catalyst, selective, oxide, catalytic, selective catalytic
2	Global and regional impacts of ammonia emissions from agricultural practices	emission, soil, ammonia emission, manure, nh3 emission, fertilizer, nh3, n2o, volatilization, pig
3	Ammonia plasma and NH_3-assisted processes in semiconductor fabrication	film, nitride, layer, deposition, thin, silicon, gan, thin film, tin, nitridation
4	Ammonia in industrial processes and material synthesis	ammonium, solution, acid, salt, aqueous, ion, chloride, sulfate, copper, carbonate
5	Ammonia management in anaerobic digestion processes	waste, removal, anaerobic, digestion, biogas, pretreatment, anaerobic digestion, recovery, treatment, effluent
6	Development and application of ammonia gas sensors	sensor, detection, ammonia gas, gas, sense, ppm, gas sensor, room, sensing, response
7	Ammonia reduction strategies in cell culture and microbial systems	growth, bacteria, cell, protein, culture, enzyme, isolate, strain, production, microbial
8	High-purity hydrogen production and purification via ammonia decomposition using membrane reactors	hydrogen, membrane, decomposition, ammonia decomposition, borane, hydrogen production, ammonia borane, bed, reactor, hydrolysis
9	Chemical hydrolysis and photolysis of amminecobalt(III) complexes	complex, nh3, ion, ligand, co, ii, iii, bond, hydrazine, state
10	Chemical reactions and derivatives of ammonia with heterocyclic and fluorinated compounds	amine, compound, acid, amino, liquid ammonia, derivative, methyl, amide, group, polymer
11	Ammonia as an energy storage medium in renewable energy systems	system, energy, power, fuel cell, cell, solar, exergy, heat, efficiency, cycle
12	Synthesis and characterization of nanomaterials via ammonia-assisted processes	ray, diffraction, microscopy, ray diffraction, synthesize, electron microscopy, morphology, spectroscopy, structure, zno
13	Aqueous ammonia for post-combustion CO_2 capture: pilot studies and process evaluation	co2, capture, dioxide, carbon dioxide, co2 capture, flue, flue gas, carbon, regeneration, solvent
14	Enhancing ammonia-water absorption processes through heat and mass transfer optimization	water, absorption, heat, ammonia water, mass, mass transfer, tube, liquid, transfer, vapor
15	Ammonia generation and utilization in NO_x emission reduction technologies for vehicles	urea, nox, exhaust, scr, diesel, nox reduction, diesel engine, ammonia urea, sapo, lean
16	Thermal decomposition and evolution of gaseous products in nitrogenous compounds	product, decomposition, pyrolysis, formation, thermal, nh3, phase, gas phase, hcn, gas
17	Enhancing combustion and emission characteristics of ammonia-based fuels in engines	combustion, flame, fuel, engine, ignition, ammonia, injection, blend, ammonia combustion, ratio

Table S19 Methanol: List of relevant and non-relevant topics and most relevant tokens per topic. Note: Topic names for the irrelevant topics (1-15) are genAI-based and not manually reviewed or refined.

ID	Name short	Most relevant tokens
ME1	Computational studies for safety & optimization	process, optimization, design, waste, industrial, wastewater, network, control, use, objective
ME2	Alternative fossil feedstocks	gas, natural, natural gas, coal, fuel, technology, syngas, synthesis gas, plant, oil
ME3	Kinetic modeling	model, kinetic, experimental, datum, rate, kinetic model, equation, parameter, experimental datum, diffusion
ME4a	Catalyst engineering	catalyst, activity, support, catalytic, prepare, surface area, surface, catalytic activity, high, ceo2
ME4b	Cu-Zn-based catalysts	zno, synthesis, catalyst, methanol synthesis, copper, al2o3, zinc, zno al2o3, oxide, zno catalyst
ME4c	Methanol surface interactions over catalysts	surface, adsorption, density functional, specie, theory, functional theory, dft, functional, mechanism, density
ME5	Novel reactor designs	reactor, bed, flow, bed reactor, feed, fix bed, fix, methanol, membrane, membrane reactor
ME6	Distillation	distillation, column, acetate, butyl, peroxide, separation, extractive, methyl acetate, ldh, distillation column
ME7	Electrocatalytic CO_2 conversion	metal, mof, framework, metal organic, organic framework, efficient, catalyst, cnt, organic, highly
ME8	Photocatalytic CO_2 conversion	tio2, photocatalytic, photocatalyst, light, reduction, visible, visible light, photo, c3n4, reduction co2
ME9	Other approaches (*e.g.*, plasma reactors)	phase, liquid, temperature, plasma, gas phase, pressure, liquid phase, slurry, gas, vapor
ME10	Direct methane-to-methanol	methane, oxidation, methane methanol, conversion, partial oxidation, partial, oxidation methane, review, ch4, catalysis
ME11	CO_2 hydrogenation	hydrogenation, co2, co2 hydrogenation, hydrogenation methanol, zro2, in2o3, methanol, hydrogenation co2, co2 conversion, synthesis co2
ME12	Industrial symbiosis & polygeneration	system, energy, solar, heat, power, efficiency, exergy, polygeneration, integrate, integration
ME13	Environmental & economic feasibility	co2, production, emission, economic, carbon, renewable, methanol, capture, energy, biomass
1	Methanol-induced heterologous protein expression in pichia pastoris	enzyme, glycerol, protein, methanol, strain, concentration, pastoris, culture, pulp, growth
2	Excited-state proton transfer dynamics in methanol solvent systems	ion, radical, transfer, molecule, state, proton, cation, molecular, photolysis, electron
3	Reactivity and coordination chemistry of methanol with metal complexes	co, iron, fe, complex, ru, iii, ruthenium, molybdenum, ome, metal
4	Nanoparticle synthesis and characterization in methanol solutions	ray, spectroscopy, microscopy, nanoparticle, electron microscopy, diffraction, ray diffraction, scan, electron, transmission
5	Electrocatalytic oxidation of methanol in alkaline media	oxidation, methanol oxidation, electrode, electrochemical, platinum, electrocatalytic, electrocatalyst, rgo, oxidation reaction, electro
6	Methanol combustion in dual-fuel and direct injection engines: performance and emission characteristics	carbon, engine, dioxide, carbon dioxide, monoxide, carbon monoxide, combustion, diesel, fuel, blend
7	Synthesis, characterization, and magnetic properties of multinuclear metal complexes	complex, ligand, ii, crystal, crystal structure, coordination, structure, magnetic, single crystal, schiff
8	Methanol extracts: antioxidant, antimicrobial, and analytical applications	water, solvent, extract, chromatography, extraction, water methanol, detection, aqueous, methanol water, mass spectrometry
9	Synthesis and reactivity of methanol-derived compounds	nmr, derivative, compound, ring, group, amine, cis, amino, phenyl, yl
10	Polymer synthesis and characterization with methanol applications	polymer, poly, polymerization, fiber, porous, mechanical, lignin, copolymer, material, composite
11	Direct methanol fuel cell performance and methanol crossover control	cell, fuel cell, membrane, fuel, methanol fuel, direct methanol, anode, dmfc, exchange membrane, nafion
12	Chemical transformations and reactions in methanol-based systems	acid, ether, ethylene, formic, formic acid, acetic, dimethyl, dimethyl ether, glycol, acetic acid
13	Zeolite catalysts in methanol conversion processes	zeolite, olefin, zsm, propylene, sapo, mto, 34, aromatic, sapo 34, methanol olefin
14	Hydrogen production through methanol reforming technologies	hydrogen, steam, reform, hydrogen production, reforming, methanol steam, steam reform, production, steam reforming, reformer
15	Methanol as a solvent and reactant in organic synthesis and metal extraction processes	alcohol, product, methyl, ethanol, reaction, ester, yield, methanol ethanol, methyl formate, biodiesel

Table S20 Olefins: List of relevant and non-relevant topics and most relevant tokens per topic. Note: Topic names for the irrelevant topics (1-10) are genAI-based and not manually reviewed or refined.

ID	Name short	Most relevant tokens
OL1	FT: Reactor designs	reactor, bed, fix, bed reactor, fix bed, flow, heat, bubble, fluidize, fluidize bed
OL2	FT: Computational studies	surface, calculation, theory, dft, density functional, barrier, functional theory, energy, mechanism, functional
OL3	FT: Reaction mechanisms	fischer, tropsch, fischer tropsch, hydrocarbon, synthesis, tropsch synthesis, chain, distribution, chain growth, product
OL4a	FT: Catalyst design	catalyst, cobalt, support, tropsch, fischer tropsch, fischer, iron, tropsch synthesis, synthesis, activity
OL4b	FT: Catalyst synthesis	ray, microscopy, xrd, diffraction, spectroscopy, electron microscopy, prepare, ray diffraction, surface area, mesoporous
OL5	FT: Feedstock	fuel, biomass, gas, liquid, gasification, natural gas, natural, coal, liquid fuel, syngas
OL6	MTO: Catalyst design	zeolite, methanol, zsm, sapo, 34, sapo 34, mto, methanol olefin, olefin, zsm zeolite
OL7	Dehydrogenation: Catalyst design	dehydrogenation, propane, propylene, oxidative dehydrogenation, oxide, oxidative, oxidation, catalyst, oxygen, propane dehydrogenation
OL8	Catalytic cracking & pyrolysis	olefin, cracking, light, light olefin, oil, crack, yield, catalytic cracking, pyrolysis, naphtha
OL9	Operational excellence	plant, furnace, paper, ethylene, paper present, petrochemical, abstract paper, producer, technology, ethylene plant
OL10	Computational studies	model, datum, simulation, optimization, modeling, parameter, network, process, use, method
OL11	Separation technologies	separation, glycol, ethylene glycol, mof, organic, organic framework, metal organic, film, membrane, framework
OL12	Olefins upgrading	review, catalysis, heterogeneous, metal, catalyst, development, recent, metathesis, catalytic, chemical
OL13	CO_2-based photo-/electrocatalysis	co2, electrochemical, electrode, reduction, copper, co2 reduction, co2 hydrogenation, electrocatalytic, graphene, photocatalytic
OL14	Methane-to-olefins innovations	ethane, ethylene, methane, acetylene, plasma, oxidative coupling, ethane ethylene, coupling methane, hydrogenation, ocm
OL15	Energy & environmental analysis	energy, process, economic, cost, emission, efficiency, production, environmental, consumption, energy efficiency
1	Ethylene regulation and plant growth enhancement by microbial interactions	gene, ethylene, protein, enzyme, acc, stress, expression, plant, biosynthesis, fatty
2	Thermal and mechanical properties of polyolefin copolymers	copolymer, polymerization, poly, polymer, ethylene, molecular weight, polyethylene, poly ethylene, copolymerization, weight
3	Chemical reaction dynamics and mechanisms in hydrocarbon systems	decomposition, reaction, pyrolysis, kinetic, temperature, product, rate, gas phase, c2h4, pressure
4	Characterizing the impact of operating conditions on emissions and detonation dynamics in combustion	flame, combustion, soot, air, emission, ignition, ozone, concentration, fuel, engine
5	Postharvest treatments to extend shelf life and improve quality of fruits	fruit, storage, ripen, mcp, quality, treatment, day, shelf, shelf life, harvest
6	Hydrogen production and co conversion in membrane reactors via water gas shift reaction	water, membrane, water gas, gas shift, carbon, shift, co2, gas, hydrogen, membrane reactor
7	Catalytic processes in organic compound transformation and environmental remediation	ethanol, acid, dehydration, ethene, hzsm, site, dechlorination, tce, alcohol, lewis
8	Chemical reactions and mechanisms of cyclopropenes and related systems	product, methyl, reaction, radical, form, cis, olefin, alkyl, elimination, trans
9	Synthesis and characterization of transition metal complexes with nitrogen and phosphorus ligands	complex, ligand, ii, bis, oligomerization, co, nmr, nickel, ethylene oligomerization, coordination
10	Innovative approaches and reactions for olefin synthesis	alkene, synthesis, reaction, alkyne, catalyze, coupling, olefination, catalyzed, palladium, olefin

Table S21 List of relevant and non-relevant topics and most relevant tokens per topic. Note: Topic names for the irrelevant topics (1-16) are genAI-based and not manually reviewed or refined.

ID	Name short	Most relevant tokens
AR1	Coal conversion	temperature, coal, pressure, tar, increase, high temperature, low temperature, decomposition, thermal, low
AR2a	Separation: MOFs, zeolites & membranes	xylene, adsorption, membrane, separation, mof, adsorbent, organic framework, framework, metal organic, xylene isomer
AR2b	Separation: Distillation	distillation, column, control, optimization, design, process, heat, extractive, extractive distillation, distillation column
AR2c	Separation: Solvents & surfactants	water, aqueous, acid, solution, sodium, surfactant, aqueous solution, salt, ion, medium
AR2d	Separation: Ionic liquids	liquid, ionic, solvent, ionic liquid, extraction, liquid liquid, separation, equilibrium, liquid equilibrium, coefficient
AR3a	Catalysed oxidation	kinetic, model, rate, constant, rate constant, datum, experimental, parameter, order, kinetic model
AR3b	Hydrocarbon dehydrogenation	surface, film, particle, size, layer, 111, monolayer, interface, coating, particle size
AR3c	Catalytic functionalization	synthesis, coupling, reaction, aryl, catalyze, palladium, efficient, mild, pot, catalyzed
AR3d	Catalytic dehydrogenation & oxidation	oxidation, styrene, oxygen, oxide, oxidize, ethylbenzene, peroxide, toluene oxidation, oxidation toluene, hydroxylation
AR4	MTA & alkane aromatization	methane, aromatization, zsm, hzsm, btx, propane, methanol, conversion, ethylene, co2
AR5	Zeolite catalysts for TDP & alkylation	zeolite, zsm, alkylation, pore, acid site, site, acidity, mesoporous, mcm, selectivity
AR6	Catalytic reformation & aromatization	catalyst, cracking, olefin, gasoline, alumina, reform, crack, octane, paraffin, reforming
AR7	Reactor design for naphtha reforming	reactor, bed, flow, bed reactor, fix, fix bed, continuous, mass transfer, flow rate, flow reactor
AR8	Integration in petrochemical processes	process, production, technology, plant, petrochemical, fuel, industry, cost, refinery, industrial
AR9	Heterocycles & functionalized aromatics	amine, aromatic, aromatic amine, derivative, compound, substitute, nitro, microwave, substitution, aldehyde
AR10	Fine chemicals & natural products	synthesis, synthetic, review, chemistry, application, strategy, aromatic, new, approach, recent
AR11	Catalytic pyrolysis of biomass	pyrolysis, lignin, biomass, bio, bio oil, catalytic pyrolysis, catalytic, fast pyrolysis, aromatic, oil
AR12	Microbial metabolic engineering	amino acid, enzyme, strain, amino, essential oil, protein, gene, plant, essential, metabolite
AR13	Hydrodeoxygenation	catalyst, hydrogenation, catalytic, support, activity, metal, catalytic activity, active, hydrogenolysis, hydrodeoxygenation
1	Health risks and environmental impact of volatile organic compounds	exposure, concentration, soil, air, risk, vocs, level, organic compound, volatile organic, health
2	Synthesis and behaviour of aromatic compounds with chiral and mesogenic properties	guest, crystal, structure, host, self, assembly, pillar, chain, interaction, supramolecular
3	Conformational dynamics and metal ion selectivity of calixarene derivatives	arene, calix, calix arene, tert, calixarene, tert butyl, rim, cone, butyl, crown
4	Synthesis and characterization of nanostructured alloys and compounds	spectroscopy, nanoparticle, microscopy, powder, electron microscopy, xrd, scan, sem, ray, diffraction
5	Synthesis and characterization of metal-organic complexes and clusters	complex, ligand, ii, co, bis, structure, ray, crystal, ru, metal
6	Synthesis and biological evaluation of aromatic derivatives as antimicrobial agents	activity, compound, antimicrobial, cell, schiff, inhibitor, dna, anti, antibacterial, derivative
7	Photocatalytic degradation of toluene in air	degradation, toluene, plasma, removal, photocatalytic, tio2, efficiency, electrochemical, discharge, electrode
8	Characterization and formation mechanisms of aromatic secondary organic aerosols	mass, spectrometry, mass spectrometry, hydrocarbon, flame, gas, gas chromatography, aromatic hydrocarbon, soot, aerosol
9	Synthesis and properties of carbon nanotubes and nanofibers	carbon, dioxide, activate carbon, nanotube, carbon dioxide, carbon nanotube, activate, cnt, carbon atom, monoxide
10	Photophysical dynamics and charge transfer in organic dyad systems	absorption, fluorescence, state, triplet, band, excited, charge, porphyrin, transfer, spectra
11	Aromaticity and stability in substituted aromatics: insights from theoretical chemistry	calculation, dft, theory, density, energy, functional, density functional, theoretical, functional theory, molecular
12	Synthesis and structural analysis of benzene derivatives	benzene, cyclohexane, phenol, benzene ring, benzene toluene, benzene derivative, substitute benzene, produce benzene, benzene cyclohexane, process benzene
13	Chromatographic techniques and cyclodextrin applications in aromatic compound analysis	chromatography, separation, phase, chromatographic, determination, stationary, method, column, capillary, stationary phase
14	Synthetic approaches in aromatic compound chemistry	acid, alcohol, ester, methyl, ketone, chloride, carboxylic, carboxylic acid, chiral, benzyl
15	Cycloadditions and radical reactions in aromatic compound synthesis	reaction, product, radical, formation, mechanism, form, intermediate, ring, pathway, addition
16	Characterization and enhancement of aromatic compounds in petroleum and rubber industries	oil, fraction, crude, resin, asphaltene, distillate, heavy, crude oil, molecular weight, petroleum

6 Supplementary information on categories

When studying the identified research trends over an even longer period of time, *i.e.*, from 1980 on, then it becomes even more clear that the remarkable transformation of research has only happened recently (see **Fig. S9**). It further underlines the robustness of the analysis presented in the main text.

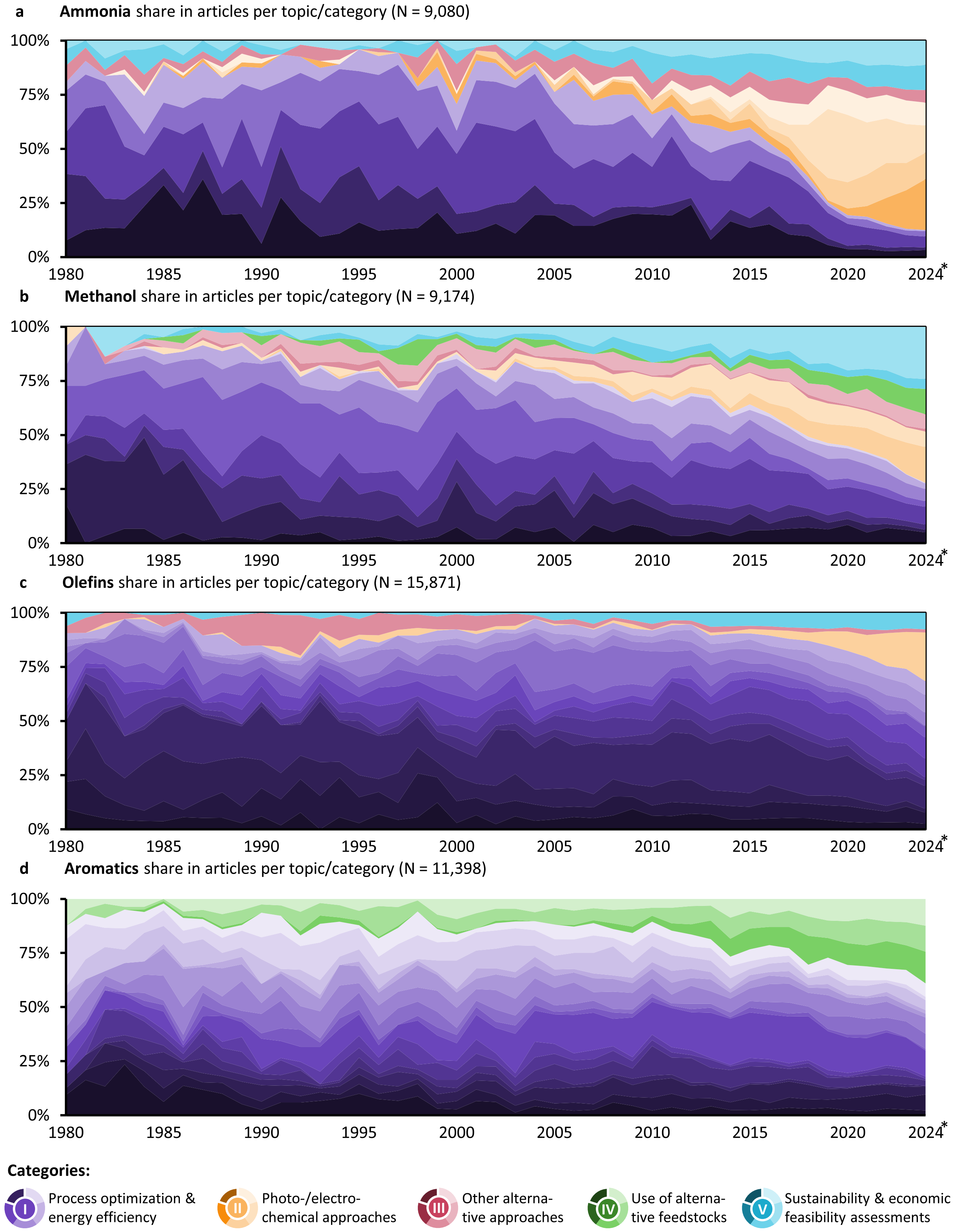

Fig. S9 High level research trends for the five categories per platform chemical (ammonia (a), methanol (b), olefins (c), aromatics (d)). *For 2024, articles are only considered until a mid-year cut-off date (see Table S4).

Table S22 Number of topics per category and platform chemical.

Chemical	Number of topics	Process optimization & energy efficiency	Photo-/electro-chemical approaches	Other alterna-tive approaches	Use of alterna-tive feedstocks	Sustainability & economic feasibility assessment
Ammonia	12	5	4	1	0	2
Methanol	15	8	2	2	1	2
Olefins	16	13	1	1	0	1
Aromatics	19	16	0	0	3	0
**Number of topics**	**62**	**42**	**7**	**4**	**4**	**5**

7 Supplementary information on topics

The following **Tables S23-S26** present details on the relevant topics that were identified per platform chemical beyond what is shown in the main text. In Figs. 3 and 4 of the main, only "ID" and "Name short" were indicated. The "Cat" column indicates the category to which each topic was assigned. The categories are: 1 Process optimization & energy efficiency; 2 Photo-/electrochemical approaches; 3 Other alternative approaches; 4 Use of alternative feedstocks; 5 Sustainability & economic feasibility assessment.

Table S23 Ammonia: Detailed information on topics. Abbreviations: HB = Haber-Bosch.

Cat	ID	Name	Name short	Description
1	AM1	Safety and optimization in Haber-Bosch plants	HB: Safety & optimization	Improving risk management and process optimization in Haber-Bosch plants
	AM2a	Metal catalysts for improved Haber-Bosch efficiency	HB: Metal catalysts	Exploring nitrogen's interaction with metal catalysts to enhance Haber-Bosch process efficiency
	AM2b	Ru catalyst design and mechanistic studies in the Haber-Bosch process	HB: Ru-based catalysts	Optimization of Ru-based catalysts and mechanisms to enhance ammonia synthesis via the Haber-Bosch process
	AM3	Process simulation and reactor design for Haber-Bosch	HB: Process simulation & reactor design	Modelling and optimization of ammonia synthesis in Haber-Bosch reactors, focusing on reactor design, thermodynamics, and catalyst effectiveness
	AM4	Syngas production technologies and processes preceding the Haber-Bosch process	Syngas production	Exploring methods for syngas generation from conventional and alternative feedstocks for ammonia synthesis
2	AM5	Catalyst design for electrochemical ammonia synthesis from nitrates	Catalysts for electrochemical synthesis from nitrates	Designing catalysts for converting nitrates to ammonia via electrochemical synthesis, enhancing yield and selectivity
	AM6	Computational modelling and catalyst design of single-atom catalysts for ammonia synthesis	Computational studies of single-atom catalysts	Exploring single-atom catalysts for efficient nitrogen reduction to ammonia via computational modelling and catalyst design
	AM7	Catalysts for electrochemical ammonia synthesis from nitrogen under ambient conditions	Catalysts for electrochemical synthesis from nitrogen	Designing non-precious metal electrocatalysts for nitrogen reduction to ammonia at ambient conditions
	AM8	Catalyst design for photochemical ammonia synthesis	Catalysts for photochemical synthesis	Enhancing photocatalytic nitrogen fixation to ammonia using novel catalyst designs and mechanisms
3	AM9	Alternative routes for ammonia synthesis (*e.g.*, plasma-assisted catalytic)	Other approaches (*e.g.*, plasma-assisted)	Exploring other approaches for sustainable ammonia synthesis (*e.g.*, low-temperature, concentrated solar power-driven, and plasma-assisted)
5	AM10	Environmental and economic feasibility of green ammonia technologies	Environmental & economic feasibility	Assessing green ammonia production methods' viability, environmental impact, and economic feasibility for sustainable energy and decarbonization
	AM11	Reviews on status of alternative routes for ammonia synthesis	Reviews on status of alternative routes	Summarizing the status for various sustainable ammonia synthesis methods beyond the Haber-Bosch process

Table S24 Methanol: Detailed information on topics.

Cat	ID	Name	Name short	Description
1	ME1	Optimization of processes and safety through computational methods	Computational studies for safety & optimization	Improving risk management and process optimization in methanol production facilities through computational methods
	ME2	Optimization of methanol production processes from alternative fossil feedstocks	Alternative fossil feedstocks	Exploring methanol production optimization from natural gas and other fossil feedstocks, focusing on technological and economic advancements
	ME3	Optimization of processes and reactors through kinetic modelling and simulation	Kinetic modelling	Modelling and simulation of methanol synthesis focusing on kinetic and thermodynamic parameters, diffusion models, and reactor dynamics
	ME4a	Optimization of methanol synthesis from syngas through catalyst engineering	Catalyst engineering	Enhancing methanol synthesis from syngas through catalyst design and optimization
	ME4b	Optimization of methanol synthesis from syngas over Cu-Zn-based catalysts	Cu-Zn-based catalysts	Optimizing Cu-Zn catalysts for enhanced methanol synthesis via syngas focusing on preparation methods, activation, and structural characteristics
	ME4c	Mechanistic simulations of methanol surface interactions over heterogeneous catalysts	Methanol surface interactions over catalysts	Exploring methanol surface interactions and reaction mechanisms on various metal and alloy surfaces via theoretical and computational approaches
	ME5	Novel reactor designs for methanol synthesis	Novel reactor designs	Exploring novel reactor designs for methanol synthesis
	ME6	Optimization of distillation process	Distillation	Enhancing methanol separation and purity via distillation techniques and control strategies
2	ME7	Electrocatalytic routes for methanol synthesis from CO_2	Electrocatalytic CO_2 conversion	Exploring catalytic materials for the electrocatalytic CO_2 reduction to methanol
	ME8	Photocatalytic routes for methanol synthesis from CO_2	Photocatalytic CO_2 conversion	Exploring catalytic materials for the photocatalytic CO_2 reduction to methanol
3	ME9	Other approaches (*e.g.*, plasma or slurry-phase reactors) for methanol synthesis	Other approaches (*e.g.*, plasma reactors)	Exploring methanol synthesis via alternative approaches (*e.g.*, slurry-phase reactors or plasma-assisted reactions) under varying conditions
	ME10	Methods for methanol synthesis via methane oxidation	Direct methane-to-methanol	Exploring direct methane-to-methanol conversion at low temperatures using various catalytic strategies
4	ME11	Catalysts for direct methanol synthesis via CO_2 hydrogenation	CO_2 hydrogenation	Enhancing direct CO_2 to methanol conversion via novel catalysts
5	ME12	Sustainable methanol production through industrial symbiosis and polygeneration	Industrial symbiosis & polygeneration	Studying methanol-power polygeneration systems aimed at simultaneously, including setups that integrate renewable energy sources like solar power
	ME13	Environmental and techno-economic assessments of sustainable methanol production	Environmental & economic feasibility	Integrating carbon capture and utilization (CCU) in methanol production as a strategy for reducing CO_2 emissions and improving sustainability

Table S25 Olefins: Detailed information on topics. Abbreviations: FT = Fischer-Tropsch, MTO = methanol-to-olefins.

Cat	ID	Name	Name short	Description
1	OL1	Reactor design for Fischer-Tropsch	FT: Reactor designs	Exploring the impact of reactor internals on hydrodynamics and heat transfer in Fischer-Tropsch bubble columns
	OL2	Computational modelling for Fischer-Tropsch	FT: Computational studies	Using computational methods to study CO and hydrogen interactions and reactions on metal surfaces in Fischer-Tropsch synthesis
	OL3	Reaction mechanisms and product formation in Fischer-Tropsch	FT: Reaction mechanisms	Exploring reaction mechanisms of Fischer-Tropsch synthesis, focusing on catalysts' impact on hydrocarbon product distribution and selectivity
	OL4a	Catalyst design for Fischer-Tropsch	FT: Catalyst design	Optimizing catalyst design for enhanced performance and selectivity of Fischer-Tropsch synthesis
	OL4b	Synthesis of catalysts for Fischer-Tropsch	FT: Catalyst synthesis	Exploring synthesis approaches for catalysts for Fischer-Tropsch synthesis
	OL5	Feedstock considerations for Fischer-Tropsch	FT: Feedstock	Studying the conversion of fossil and biogenic feedstocks into liquid fuels via Fischer-Tropsch synthesis and related processes
	OL6	Catalyst design for methanol-to-olefin conversion	MTO: Catalyst design	Optimizing catalysts for methanol-to-olefin conversion focusing on their synthesis, deactivation, and structural modifications
	OL7	Catalyst design for propane and ethane dehydrogenation	Dehydrogenation: Catalyst design	Exploring catalyst compositions for enhanced selectivity and stability in propane and ethane dehydrogenation to propylene and ethylene
	OL8	Optimization of catalytic cracking and pyrolysis	Catalytic cracking & pyrolysis	Studying catalytic cracking and pyrolysis for enhanced light olefins production from various hydrocarbon sources
	OL9	Optimization of ethylene production plants and processes	Operational excellence	Optimizing ethylene plant operations, including efficiency, safety, and reliability
	OL10	Computational modelling and simulation for olefins production optimization	Computational studies	Optimizing olefin production using data-driven computational models
	OL11	Novel separation technologies	Separation technologies	Exploring porous materials and membranes for efficient gas separation in petrochemical processes
	OL12	Catalytic processes for olefins upgrading	Olefins upgrading	Studying olefin metathesis and upgrading via catalytic processes for sustainable production of complex olefins
2	OL13	Catalyst design for CO_2-based photo- and electrocatalytic routes	CO_2-based photo-/electrocatalysis	Designing catalysts for converting CO_2 into olefins via photo- and electrocatalysis
3	OL14	Methane-to-olefins processes	Methane-to-olefins innovations	Exploring methane-to-olefins conversion using various catalytic and plasma processes
5	OL15	Energy and emissions analysis for olefins production ecosystems	Energy & environmental analysis	Increasing transparency on energy use and emissions of olefins production ecosystems

Table S26 Aromatics: Detailed information on topics for aromatics. Abbreviations: HDO = hydrodeoxygenation, MTA = methanol-to-aromatics, MOFs = Metal-organic frameworks, TPD = toluene disproportionation.

Cat	ID	Name	Name short	Description
1	AR1	Coal conversion to aromatic compounds	Coal conversion	Exploring aromatic compounds in coal and different coal conversion processes, focusing on pyrolysis, liquefaction, and gasification under various conditions
	AR2a	Separation of aromatic compounds	Separation: MOFs, zeolites & membranes	Studying separation techniques of aromatic compounds, focusing on metal-organic frameworks, zeolites, and membrane technologies
	AR2b	Distillation of aromatic compounds	Separation: Distillation	Exploring the design, control, and optimization of distillation technologies for energy-efficient separation of aromatic compounds
	AR2c	Solvents & surfactants for separation of aromatic compounds	Separation: Solvents & surfactants	Enhancing extraction and separation techniques for aromatic compounds (*e.g.*, distillation), focusing on solvents and surfactants
	AR2d	Ionic liquid as solvents for separation of aromatic compounds	Separation: Ionic liquids	Exploring selective recovery of aromatic compounds, focusing on different ionic liquids
	AR3a	Kinetics and mechanisms of catalysed oxidation of aromatic compounds	Catalysed oxidation	Exploring the kinetics and mechanisms of oxidation of organic compounds over various catalysts
	AR3b	Catalytic dehydrogenation of hydrocarbons	Hydrocarbon dehydrogenation	Exploring hydrocarbon adsorption and dehydrogenation on various surfaces in the context of aromatic compounds
	AR3c	Catalytic methodologies for functionalizing aromatic compounds	Catalytic functionalization	Exploring catalytic methodologies for functionalization of aromatic compounds
	AR3d	Catalytic dehydrogenation and oxidation for aromatic compounds	Catalytic dehydrogenation & oxidation	Exploring various catalytic processes for the oxidative dehydrogenation and dehydrogenation of aromatic compounds (*e.g.*, ethylbenzene)
	AR4	Methanol-to-aromatics and alkane aromatization	MTA & alkane aromatization	Enhancing methanol-to-aromatics and alkane (*e.g.*, methane) aromatization via different catalysts and reaction mechanisms
	AR5	Zeolite catalysts in toluene disproportionation and alkylation	Zeolite catalysts for TDP & alkylation	Enhancing p-xylene selectivity in toluene transformation (*e.g.*, alkylation and disproportionation) via zeolite catalyst optimization
	AR6	Catalytic reforming and aromatization	Catalytic reformation & aromatization	Enhancing catalytic processes for enhancing light hydrocarbons' conversion into aromatics, focusing on yield and selectivity
	AR7	Novel reactor design for naphtha reforming to aromatic compounds	Reactor design for naphtha reforming	Designing novel reactors for efficient naphtha reforming, enhancing aromatics production with optimized conditions
	AR8	Integration in petrochemical processes towards aromatics	Integration in petrochemical processes	Optimization of petrochemical processes for aromatics production and integration within refineries
	AR9	Approaches for constructing heterocycles and functionalized aromatics	Heterocycles & functionalized aromatics	Exploring chemical synthesis methods for various aromatic compounds, including heterocycles and functionalized aromatics
	AR10	Alternative approaches for aromatic natural products and fine chemicals	Fine chemicals & natural products	Exploring various approaches for synthesizing aromatic natural products and fine chemicals (from key aromatic compounds)
4	AR11	Catalytic pyrolysis of biomass and waste materials for synthesis of aromatic compounds	Catalytic pyrolysis of biomass	Enhancing aromatic compound production via catalytic co-pyrolysis of various biomass and waste materials
	AR12	Microbial metabolic engineering for aromatic production	Microbial metabolic engineering	Engineering microbes for aromatic compound production via metabolic pathway modification
	AR13	Catalytic hydrodeoxygenation processes for synthesis of aromatic compounds	Hydrodeoxygenation	Optimizing catalytic hydrodeoxygenation processes for efficient biomass conversion into aromatics

References

1 P. Tautorat, B. Lalin, T. S. Schmidt and B. Steffen, *J. Clean. Prod.*, 2023, **407**, 137055.

2 O. Kononova, T. He, H. Huo, A. Trewartha, E. A. Olivetti and G. Ceder, *iScience*, 2021, **24**, 102155.

3 M. W. Callaghan, J. C. Minx and P. M. Forster, *Nat. Clim. Chang.*, 2020, **10**, 118–123.

4 J. Lu and G. F. Nemet, *Environ. Res. Lett.*, 2020, **15**, 123003.

5 M. Visser, N. J. van Eck and L. Waltman, *Quant. Sci. Stud.*, 2021, **2**, 20–41.

6 S. Syed and M. Spruit, in *2017 IEEE International Conference on Data Science and Advanced Analytics (DSAA)*, 2017, pp. 165–174.

7 A. Martín-Martín, R. Costas, T. Van Leeuwen and E. Delgado López-Cózar, *J. Informetr.*, 2018, **12**, 819–841.

8 D. M. Blei, A. Y. Ng and M. I. Jordan, *J. Mach. Learn. Res.*, 2003, **3**, 993–1022.

9 I. Vayansky and S. A. P. Kumar, *Inf. Syst.*, 2020, **94**, 101582.

10 M. E. Rose and J. R. Kitchin, *SoftwareX*, 2019, **10**, 100263.

11 A. Schofield, M. Magnusson, L. Thompson and D. M. Mimno, *Pre-Processing for Latent Dirichlet Allocation*, 2017.

12 M. Honnibal, I. Montani, S. van Landeghem and A. Boyd, *spaCy: Industrial-strength Natural Language Processing in Python (Version 3.2.2)*, Zenodo, 2020.

13 K. Smelyakov, D. Karachevtsev, D. Kulemza, Y. Samoilenko, O. Patlan and A. Chupryna, *2020 IEEE Int. Conf. Probl. Infocommunications Sci. Technol. PIC S T 2020 - Proc.*, 2021, 187–191.

14 L. Hagen, *Inf. Process. Manag.*, 2018, **54**, 1292–1307.

15 Y. Zhang, R. Jin and Z. H. Zhou, *Int. J. Mach. Learn. Cybern.*, 2010, **1**, 43–52.

16 F. Pedregosa, G. Varoquaux, A. Gramfort, V. Michel, B. Thirion, O. Grisel, M. Blondel, P. Prettenhofer, R. Weiss, V. Dubourg, J. Vanderplas, A. Passos, D. Cournapeau, M. Brucher, M. Perrot and E. Duchesnay, *J. Mach. Learn. Res.*, 2011, **12**, 2825–2830.

17 J. Chang, S. Gerrish, C. Wang, J. Boyd-graber and D. Blei, in *Advances in Neural Information Processing Systems*, eds. Y. Bengio, D. Schuurmans, J. Lafferty, C. Williams and A. Culotta, Curran Associates, Inc., 2009, vol. 22.

18 D. Mimno, H. Wallach, E. Talley, M. Leenders and A. McCallum, in *Proceedings of the 2011 Conference on Empirical Methods in Natural Language Processing*, eds. R. Barzilay and M. Johnson, Association for Computational Linguistics, Edinburgh, Scotland, UK., 2011, pp. 262–272.

19 T. L. Griffiths and M. Steyvers, *Proc. Natl. Acad. Sci. U. S. A.*, 2004, **101**, 5228–5235.

20 J. Bergstra and Y. Bengio, *J. Mach. Learn. Res.*, 2012, **13**, 281–305.

21 M. Konrad, *WZBSocialScienceCenter/tmtoolkit: Version 0.11.0 (Version 0.11.0)*, Zenodo, 2022.

22 C. Sievert and K. Shirley, in *Proceedings of the Workshop on Interactive Language Learning, Visualization, and Interfaces*, eds. J. Chuang, S. Green, M. Hearst, J. Heer and P. Koehn, Association for Computational Linguistics, Baltimore, Maryland, USA, 2014, pp. 63–70.

23 Plotly Technologies Inc., *Plotly Technologies Inc.*, 2015, https://plot.ly.
